# A unicentric cross-sectional observational study on chronic intestinal inflammation in total colonic aganglionosis: beware of an underestimated condition

**DOI:** 10.1186/s13023-023-02958-1

**Published:** 2023-10-27

**Authors:** M Erculiani, F Poluzzi, G Mottadelli, E Felici, Novi ML, M Caraccia, A Grandi, S Casella, L Giacometti, G Montobbio, I Ceccherini, E Di Marco, C Bonaretti, R Biassoni, M Squillario, A Pietrantoni, V Villanacci, A Pini Prato

**Affiliations:** 1 Umberto Bosio Center for Digestive Diseases, AO SS Antonio e Biagio e Cesare Arrigo, Alessandria, Italy; 2 UOSD Laboratory of Genetics and Genomics of Rare Diseases, IRCCS Istituto Giannina Gaslini, University of Genoa, Genoa, Italy; 3 Central Laboratory, Giannina Gaslini Institute, Genoa, Italy; 4 Molecular Diagnostic, Giannina Gaslini Institute, Genoa, Italy; 5 IRCCS Ospedale Policlinico San Martino Genoa, Genoa, Italy; 6 Institute of Pathology, ASST-Spedali Civili, University of Brescia, Brescia, Italy; 7 Division of Pathology, Department of Health Sciences, University of Eastern Piedmont, Novara, Italy

**Keywords:** Hirschsprung, Crohn, Enterocolitis, Inflammatory Bowel Diseases, Endoscopy, Inflammation, Total colonic aganglionosis

## Abstract

**Background:**

Inflammatory Bowel Diseases (IBD) are known to occur in association with Hirschsprung disease (HSCR). Most of cases are represented by Crohn Disease (CD) occurring in patients with Total Colonic Aganglionosis (TCSA) with an estimated prevalence of around 2%. Based on these considerations and on a number of provisional data belonging to our Center for Digestive Diseases, we developed a unicentric cross-sectional observational study aimed at describing phenotype, genotype, pathology and metagenomics of all patients with TCSA and Crohn-like lesions.

**Results:**

Out of a series of 62 eligible TCSA patients, 48 fulfilled inclusion criteria and were enrolled in the study. Ten patients did not complete the study due to non-compliance or withdrawal of consent and were subsequently dropped out. A total of 38 patients completed the study. All patients were tested for chronic intestinal inflammation by a combination of fecal calprotectine (FC) or occult fecal blood (OFB) and underwent fecal metagenomics. Nineteen (50%) tested positive for FC, OFB, or both and subsequently underwent retrograde ileoscopy. Fourteen patients (36.8%) presented Crohn-like lesions, occurring after a median of 11.5 years after surgery (range 8 months − 21.5 years). No statistically significant differences regarding demographic, phenotype and genotype were observed comparing patients with and without lesions, except for need for blood transfusion that was more frequent in those with lesions. Faecal microbiome of patients with lesions (not that of caregivers) was less biodiverse and characterized by a reduction of *Bacteroidetes*, and an overabundance of *Proteobacteria*. FC tested negative in 3/14 patients with lesions (21%).

**Conclusions:**

Our study demonstrated an impressive 10-folds higher incidence of chronic inflammation in TCSA. Up to 50% of patients may develop IBD-like lesions postoperatively. Nonetheless, we failed in identifying specific risk factors to be used to implement prevention strategies. Based on the results of our study, we suggest screening all TCSA patients with retrograde ileoscopy regardless of FC/OFB values. The frequency of endoscopic assessments and the role of FC/OFB screening in prompting endoscopy is yet to be determined.

**Supplementary Information:**

The online version contains supplementary material available at 10.1186/s13023-023-02958-1.

## Introduction

Hirschsprung disease (HSCR) is a rare congenital anomaly of the Enteric Nervous System characterized by aganglionosis involving the rectum with variable proximal extension [1]. The disease may be complicated by a potentially fatal complication occurring in around 25% of patients called Hirschsprung Associated Enterocolitis (HAEC) that carries significant morbidity and mortality [2, 3]. HAEC pathophysiology is still poorly understood but potential factors include intestinal dysmotility, intestinal barrier dysfunction, impaired mucosal immunity and dysbiosis [4]. Since our first report in 2010 [5], several studies suggested a key-role of intestinal dysbiosis in the pathogenesis of HAEC [6–17].

A number of analogies link HAEC with inflammatory bowel diseases (IBD) both from a clinical and from a histological point of view. Of note, symptoms observed in patients with toxic megacolon occurring in acute severe attacks of Ulcerative Colitis (UC) are very similar to those observed in patients experiencing HAEC [18–22].

During the last two decades, some Authors addressed the association HSCR + IBD. The first case was reported by Kessler in 1999 [23] but more than half cases have been published only recently, since 2014 [24–35]. Even if less than 100 cases have been reported so far, Bernstein and colleagues suggested that over 2% of HSCR patients may develop IBD and that the likelihood of being diagnosed with IBD, mostly Crohn Disease (CD), is 12-fold higher compared to the general population [30]. Accordingly, Nakamura and co-workers in 2018 suggested that CD is the most frequently reported IBD predominantly involving total colonic aganglionosis (TCSA) [28].

Lacher and colleagues in 2010 ruled out the presence of typical IBD-predisposing genetic background (NOD2 variants) in HSCR patients [36]. On the other hand, Frykman et al. observed that serum biomarkers for IBD, such as anti-*Saccharomyces cerevisiae* mannan antibodies and outer membrane porin C, turned out to be elevated in HAEC patients [29]. The authors suggested that HAEC and CD might share gut microbial-host immune responses [29].

Based on our previous report of IBD-like lesions in patients with HSCR [37], on personal unpublished data and on above-mentioned literature, we implemented a *single-center observational cross-over study* aimed at providing a prospective epidemiological overview on clinical features, genetics, histology, endoscopy, and metagenomics of patients with IBD-like intestinal lesions developed after reconstructive surgery in TCSA.

## Results

A total of 62 patients with TCSA are being followed up in our Center. Fourteen of these patients have been excluded (7 refused to participate, 3 carried permanent ileostomy, 2 underwent pull-through less than 6 months before enrollment, 2 had significant linguistic barrier). A further 10 patients who accepted to participate were enrolled but subsequently dropped-out due to withdrawal of consent (1 patient), lack of fulfillment for Fecal Calprotectine (FC), Occult Fecal Blood (OFB) or both regardless of multiple reminders (6 patients), and refusal to perform retrograde ileoscopy despite positive CF, OFB or both (3 patients). A total of 38 TCSA patients therefore completed the study and represented the focus of this paper (61.3%) (Supplementary Fig. 1).

### Demography

Male to female ratio was 2.2:1. Median age at pull-through was 13 months (range 6 months to 11 years). Median age at enrollment was 11.5 years (range 18 months to 35 years). Median timespan between pull-through and enrollment was 9.5 years (range 8 months to 26.5 years). A familial history of HSCR was reported by 8 patients out of 37 with available data on this regard (1 patient was adopted and nothing was known regarding biological parents and relatives). A 27.6% of familial transmission was identified. None reported a family history for IBD.

### Phenotype

A total of 35 patients had complete data regarding HAEC status. Twenty-three (65.7%) had at least one episode of HAEC in their past clinical history. Nine (23.7%) suffered from associated anomalies or syndromes, including Shah-Waardenburg, Down, and Turner Syndromes, MEN2, CAKUT and congenital heart disease. The exact extent of aganglionosis involving the small bowel could be retrieved only in a minority of the patients (14/38 = 37%) and turned out to be between 5 and 100 cm. In fact, in most cases (20 patients) a general definition of terminal ileum involvement was reported without specific mention to exact measurement. In 4 patients this datum was completely missing. Twenty-four patients (66.7%) underwent an endorectal pull-through (either laparotomic, laparoscopic or robotic) always without a J-pouch, 11 (30.5%) a retrorectal (Duhamel, Lester-Martin or their modifications), 1 (2.8%) a perirectal (Swenson) without J-pouch. In 10 patients (27.8%) the procedure was a redo pull-through. In 2 patients no data regarding the pull-through were available.

### Genotype

Thirty-three patients (87%) completed molecular genetics as 5 did not consent to the DNA extraction. A total of 14 RET variants (42%) were identified, 5 being transmitted (2 maternal and 3 paternal), 5 being *de novo*. In 4 cases transmission could not be assessed.

### Growth parameters

Mean weight at enrollment was variable given the wide age-range at inclusion in the study. We therefore calculated Z-scores and BMI. Mean Z-scores for prepubertal patients was − 1,28±1,27 whereas BMI for postpubertal patients was 20,05±2,2.

### Questionnaire

Questionnaire was answered by all patients and/or caregivers (100%). Loose to liquid stools were described by 97.4%. Night-time stools by 71.1%. More than 5 bowel movements were reported by 65.8%. Abdominal pain was reported by 60.5%, dietary limitations by 57.9%, abdominal distension by 34.2% as was rectal bleeding. Limitation of daily activities was complained by 44.7%. Iron supplementation was needed by 26.3%. Blood transfusion was received by 7.9% of patients between 6 and 12 months after the pull-through. Two of these patients required stoma formation or bowel resection to deal with this unresponsive blood loss and subsequent anemia. Antibiotic or anti-inflammatory medications were administered to 13.2% and 5.3% of patients, respectively.

### FC/OFB screening, pathology and endoscopy

Nineteen patients (50%) tested positive to either FC (9/19), OFB (3/19) or both (7/19) and underwent retrograde ileoscopy with biopsies. In 14 (73.7%) inflammation was confirmed by pathology reports. Eleven were diagnosed with Crohn-like (CD-like), 3 with Eosinophilic Enteritis (EE). All 14 patients were categorized as Cases (36.8% prevalence − 95% CI 24–53%). In all Cases but one the Pathologist reported active inflammation. One showed chronic inflammation. In 5 patients, endoscopy and histology were discordant. Two patients with normal endoscopic appearance were diagnosed with EE, 1 with CD-like. These patients were categorized as Cases. Two with isolated ulcerations just above the anal canal turned out to have normal histology at biopsies sampled at the margins of the lesions and were categorized as Controls.

### Focus on patients with IBD-like lesions (n = 14)

This section will focus on 14 patients defined as Cases (Table 1) and on the comparison of most salient data with those of the 24 patients without lesions, defined as Controls (Table 2).

**Table 1:** Overall features of the 14 patients with chronic intestinal (IBD-like) inflammation (either EE or CD-like). A positive HAEC status means that the patient experienced previous episodes of enterocolitis. All but one patients (ID 1) had pathological evidence of acute inflammation or infiltrate

ID	Sex	Age [years]	Type of P-T	Pathology	Comorbidities	RET mut	Years from P-T	FC [µg/g]	OFB	SEMA-CD	PCDAI	HAEC status
1	F	1,5	ERPT	EE	None	No	0.6	16	Positive	0	5	Positive
2	F	2,4	ERPT	EE	None	Yes	1.4	1398	Negative	0	10	Negative
3	F	4.2	ERPT	CD-Like	None	N.A.	3.4	1072	Negative	2	25	Positive
4	F	5.6	ERPT	EE	None	Yes	4.5	74	Positive	2	10	Negative
5	M	6.4	ERPT	CD-Like	WS4	No	3.7	130	Negative	3	15	Negative
6	F	8.6	ERPT	CD-Like	None	No	7.3	1710	Positive	3	55	Negative
7	M	9.8	ERPT	CD-Like	CAKUT	No	8.3	1406	Positive	2	25	Positive
8	M	16.4	ERPT	CD-Like	None	No	15.5	590	Negative	2	5	Negative
9	M	17.1	Duhamel	CD-Like	None	No	16.2	1200	Positive	3	10	Positive
10	M	19.1	ERPT	CD-Like	None	Yes	17.0	823	Positive	3	17,5	Negative
11	M	22,1	Swenson	CD-Like	None	No	18.4	379	Positive	0	15	Positive
12	M	22.6	Duhamel	CD-Like	None	N.A.	21.7	83	Positive	2	20	Positive
13	M	23.8	ERPT	CD-Like	None	N.A.	14.7	366	Negative	2	15	Positive
14	M	24.1	Lester-Martin	CD-Like	None	No	21.8	318	Negative	3	20	Positive

**Legend** – *ID* = Identification; *M* = Male; *F* = Female; *P-T* = Pull-Through; *FC* = Fecal Calprotectine; *OFB* = Occult Fecal Blood; *SEMA-CD* = Simplified Endoscopic Mucosal Assessment for Crohn’s Disease; *PCDAI* = Pediatric Crohn Disease Activity Index; *HAEC* = Hirschsprung Associated Enterocolitis; *CD-Like* = Crohn-Like; *EE* = Eosinophilic Enteritis; *ERPT* = Endorectal Pull-Through; *CAKUT* = Congenital Anomalies of the Kidney and Urinary Tract; *WS4* = Waardenburg-Saha type 4

**Table 2:** Overall features of Cases and Controls who were compared to identify risk factors or early biomarkers of disease. FC was significantly higher in Cases compared to Controls active as a relatively reliable biological marker though with a number of false negative results. Amongst clinical data, only limitation of daily activity proved to be significantly more frequent in Cases, but the significance falls if correction for multiple testing is applied

Variable	Cases (n = 14)	Controls (n = 24)	*p-value*
**Gender – n (%)**			
Males Females	9 (64) 5 (36)	17 (71) 7 (29)	*p = 0.7281*
Type of Pull-through – n (%)*			
Retrorectal with pouch Endorectal or Perirectal	3 (21) 11 (79)	9 (37.5) 15 (62.5)	*p = 0.4722*
Redo – n (%)	3 (21)	7 (29)	
**Age at enrolment [years] – median (range)**	13.1 (1.5–24.2)	11.5 (1.7–35)	*p = 0.9044*
**Timespan from surgery [years] – median (range)**	11.5 (0.7–21.7)	9.5 (0.9–26.5)	*p = 0.8684*
**Syndromes or associated anomalies – n (%)**	2 (14)	6 (25)	*p = 0.6836*
Positive familial history for Hirschsprung – n (%)*	3 (21)	5 (22)	*p = 1.000*
RET mutations – n (%)*	3 (30)	11 (48)	*p = 0.1750*
Positive HAEC status – n (%)*	8 (57)	8 (36)	*p = 0.1870*
**Median (range) of FC [µg/g]**	485 (16-1710)	50 (9-1312)	***p = 0.0012***
**Positive OFB (%)**	8 (57%)	3 (12%)	***p = 0.0076***
**Questionnaire results – n (%)**			
**Abdominal pain**	7 (50)	16 (67)	*p = 0.4924*
**Distended abdomen**	4 (29)	9 (37.5)	*p = 0.7281*
**Rectal bleeding**	7 (50)	5 (21)	*p = 0.0812*
**Stools > 5**	9 (64)	16 (67)	*p = 1.0000*
**Liquid stools**	8 (57)	10 (42)	*p = 0.5030*
**Night-time stools**	11 (79)	16 (67)	*p = 0.4882*
**Limitation of daily activities**	8 (57)	9 (38)	*p = 0.3176*
**Dietary limitations**	10 (71)	12 (50)	*p = 0.3087*
**Iron supplementation**	5 (36)	5 (24)	*p = 0.4485*
**Blood transfusion**	**3 (21)**	**0 (0)**	***p = 0.0431***
**Antibiotic administration**	3 (21)	3 (13)	*p = 0.6497*
**Anti-inflammatory drugs**	1 (7)	1 (4)	*p = 1.0000*

**Legend**: *n* = number; *RET* = RE-arranged during Transfection or RET proto-oncogene; *HAEC* = Hirschsprung Associated Enterocolitis; *FC* = Fecal Calprotectine; *OFB* = Occult Fecal Blood; * = not all the patients had comprehensive data in these fields, so the denominator changes with subsequent effects on percentages and significance.

#### Comparison of demographics, phenotype, genotype, growth parameters, and Questionnaire

Statistical analysis failed in identifying significant differences regarding demographics, phenotype, genotype, and growth parameters comparing Cases and Controls. Of note, extent of aganglionosis proved to longer in Cases but the difference did not reach statistical significance (37 cm vs. 9.6 cm of mean ileal involvement in Cases and Controls, respectively - *p = 0.0943*). Similarly, the mean Z-score in prepubertal Cases turned out to be lower compared but the difference did not reach statistical significance (-1,90±0,99 vs. 0,79±1,29 of Z-scores in Cases and Controls, respectively – *p = 0.0805*). The only significance was identified when addressing the need for blood transfusions that was reported in the questionnaire more frequently by Cases (*p = 0.0431*) (Table 2).

#### FC/OFB screening results

FC tested positive in 11 (79%). OFB tested positive in 8 (57%). Both FC and OFB tested positive in 5 (36%). Median FC value of Cases was 485 µg/g (range16 to 1710 µg/g). FC tested negative in 3 patients with lesions (Table 1). Sensitivity, and specificity of FC in identifying lesions turned out to be 79% and 83%, respectively. Those of OFB were 57% and 87%, respectively.

#### Pathology reports

**Fig. 1:**
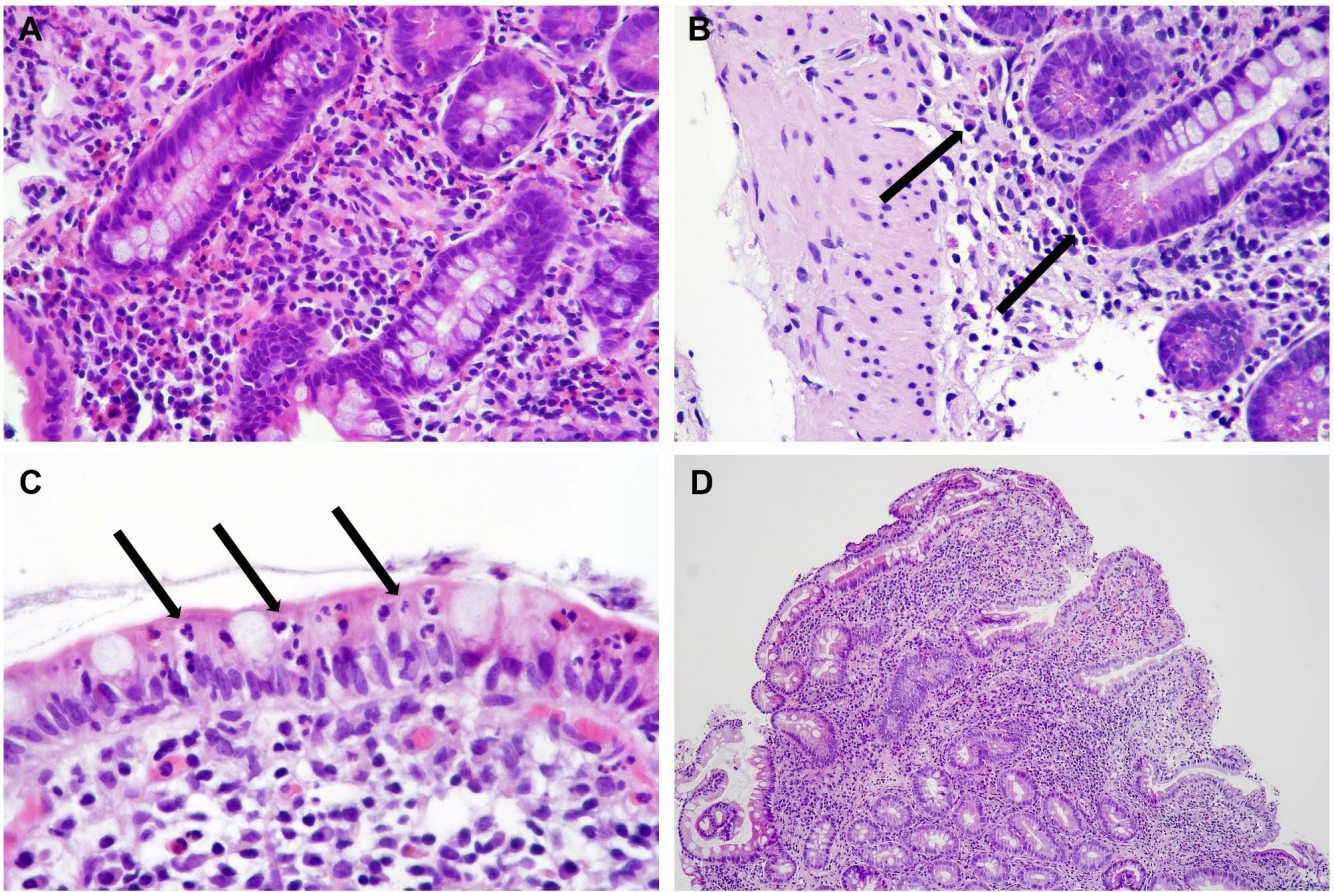
**A** to **D** belong to patients with either EE or CD-like lesions. **A** - increased eosinophilic infiltrate in lamina propria (original magnification: 40x). **B** - basal plasmacytosis (arrows; original magnification: 40x). **C** - intense neutrophilic infiltrate with aggressive neutrophils in the mucosal lining (arrows; original magnification: 60x). **D** - atrophy (original magnification: 10x)

We identified 11 patients with CD-Like and 3 with EE. Those with EE proved to be significantly younger compared to those with CD-Like (mean age at enrollment 38 ± 26 months vs. 190 ± 88 months, *p = 0.0142*). Signs of chronicity were predominantly represented by architectural distortion, basal plasmocytosis, pseudopyloric metaplasia and fibrosis whereas those of activity by erosion, ulceration, and aggressive neutrophilic and/or eosinophilic infiltrate in lamina propria. In 11 out of 14 Cases we could observe an eosinophilic infiltrate with more than 50 elements in a X40 magnification field. The lesions were consistently aggressive and never confined to the superficial lamina propria (Fig. 1 – A to D).

#### Endoscopic severity (SEMA-CD) and Disease activity (PCDAI)

**Fig. 2:**
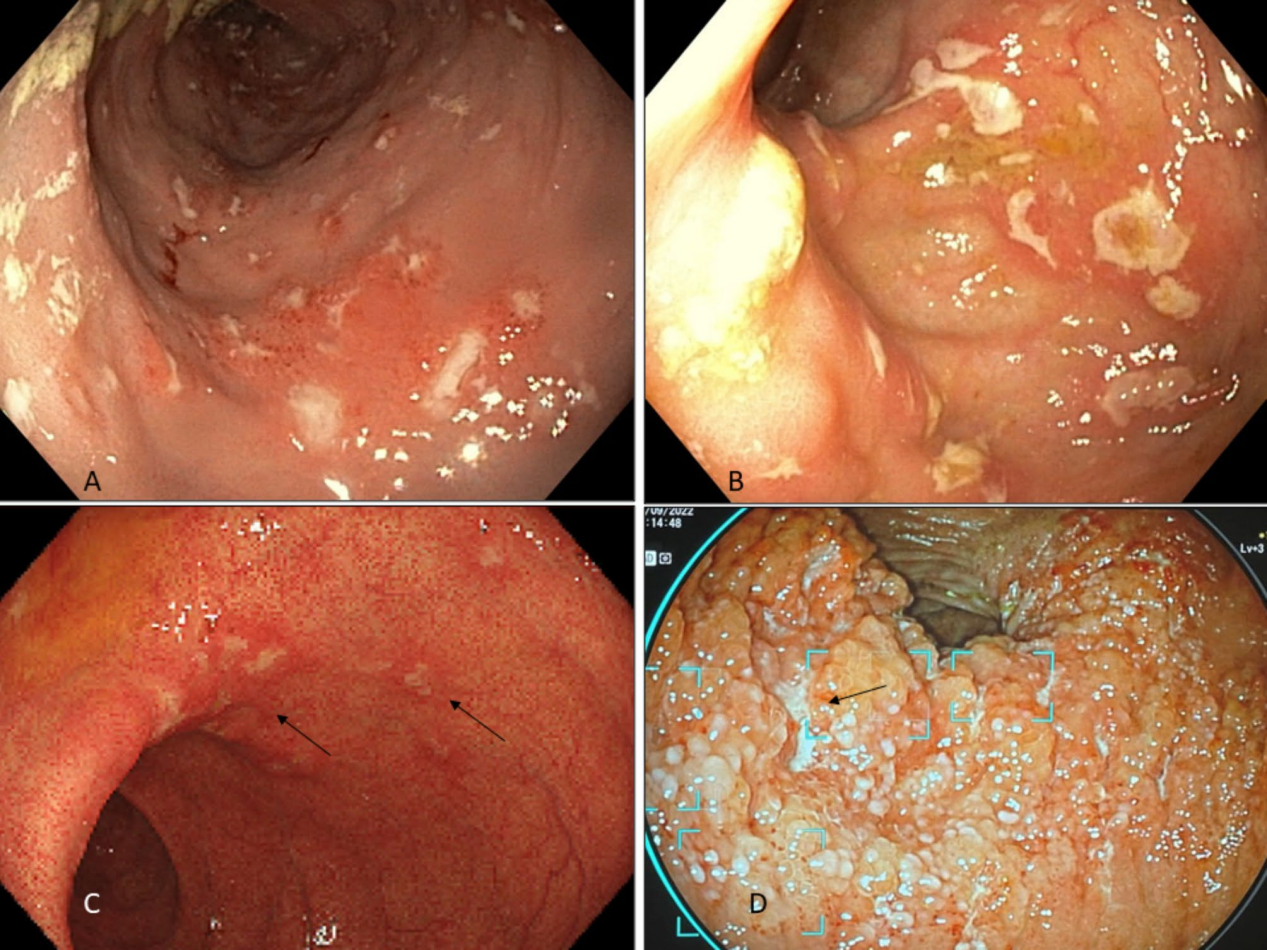
**A** to **D** are endoscopic views of patients with IBD-like lesions. **A** - large widespread aphtoid ulcerations; **B** - large aphtoid ulcerations; **C** - small aphtoid ulcerations (black arrows), oedema and hyperemia of the mucosa; **D** - pseudopolypoid lesions and ulcerations (black arrow)

According to Simplified Endoscopic Mucosal Assessment for Crohn’s Disease (SEMA-CD) we detected 3 patients with grade 0, 6 with grade 2, and 5 with grade 3 endoscopic severity score (Fig. 2 - A to D). Given the absence of severe non-negotiable strictures, we did not report any grade 4. All patients with endoscopically evident lesions (11 out of 14 who were defined as Cases based on pathology reports) showed a caudo-cranial gradient with most lesions gathered close to the sphincters and normalization of endoscopic appearance between 30 and 55 cm from the anal verge. With the Pediatric Crohn Disease Activity Index (PCDAI) index we reported 5 patients scoring 10 or less, 8 scoring between 10 and 30, 1 scoring 55. Median PCDAI score was 15 (range between 5 and 55) without statistically significant differences comparing Cases to Controls (*p = 0.0766*).

### Metagenomics

#### Composition – visual microbiome analysis

**Fig. 3:**
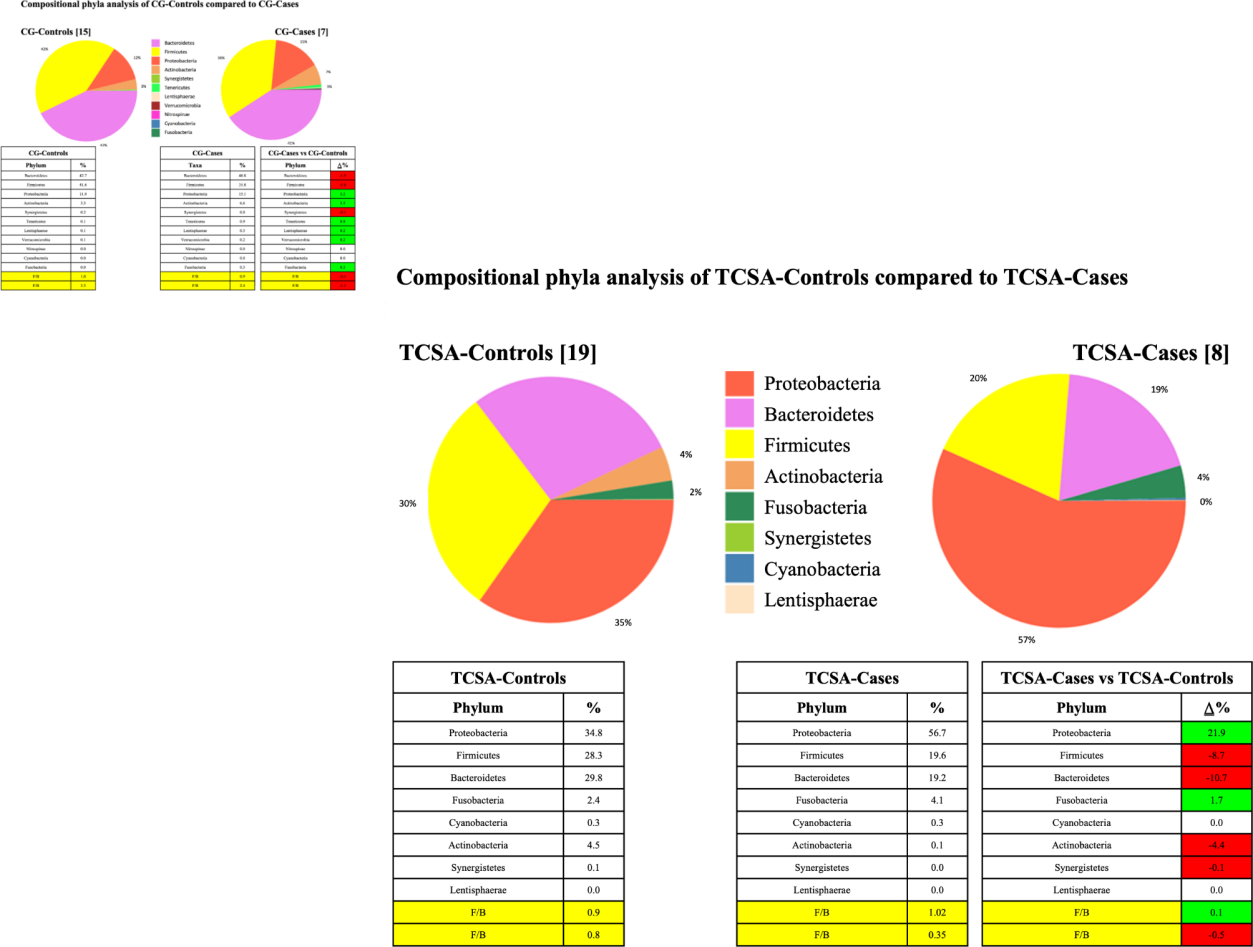
**Upper left quadrant** - Relative abundance of phyla distribution of the microbiome of Care Givers with numbers inside square brackets indicating the number of individuals in each group **Lower right quadrant** - Phyla analysis was performed on the TCSA patients microbiome grouped in Cases (with inflammation, TCSA-Cases) or Controls (without inflammation, TCSA-Controls). Numbers inside square brackets indicate the number of individuals in each group. Of note, some stools samples did not pass the internal quality control thus reducing the total number of patients in each group. In each panel, the list indicating the values of the differential abundance for each phylum between cases and controls is shown on the right. On the bottom, the right (Δ%) indicates the differential abundances between TCSA-Cases compared to TCSA-Controls green background, an increase in % abundance the red background, showed a decrease in % in TCSA-Cases.

*Bacteroidetes and Firmicutes* proved to be the most represented microbial group in the stools of caregivers. *Proteobacteria* were less abundant. The difference in abundance between the two groups of caregivers with was less than 6% for all the analyzed microbial phyla. When addressing TCSA patients with or without IBD-like lesions, the microbiome was characterized by a preponderance of *Proteobacteria* (mostly *Enterobacteriaceae*) in Cases, which proved to be nearly two-folds higher than that of Controls (50.4% vs. 26.9% median). In Cases we could observe a relevant decrease of relative abundance of *Firmicutes* and *Bacteroidetes* and nearly the disappearance of *Actinobacteria w*ith a *Firmicutes* to *Proteobacteria* (F/P) ratio which proved to be lower in Cases compared to Controls (Fig. 3**).** Patients with trisomy 21 were analyzed separately due to a potential bias related to immune dysregulations [38–40].

### Alpha and beta diversity indexes

**Fig. 4:**
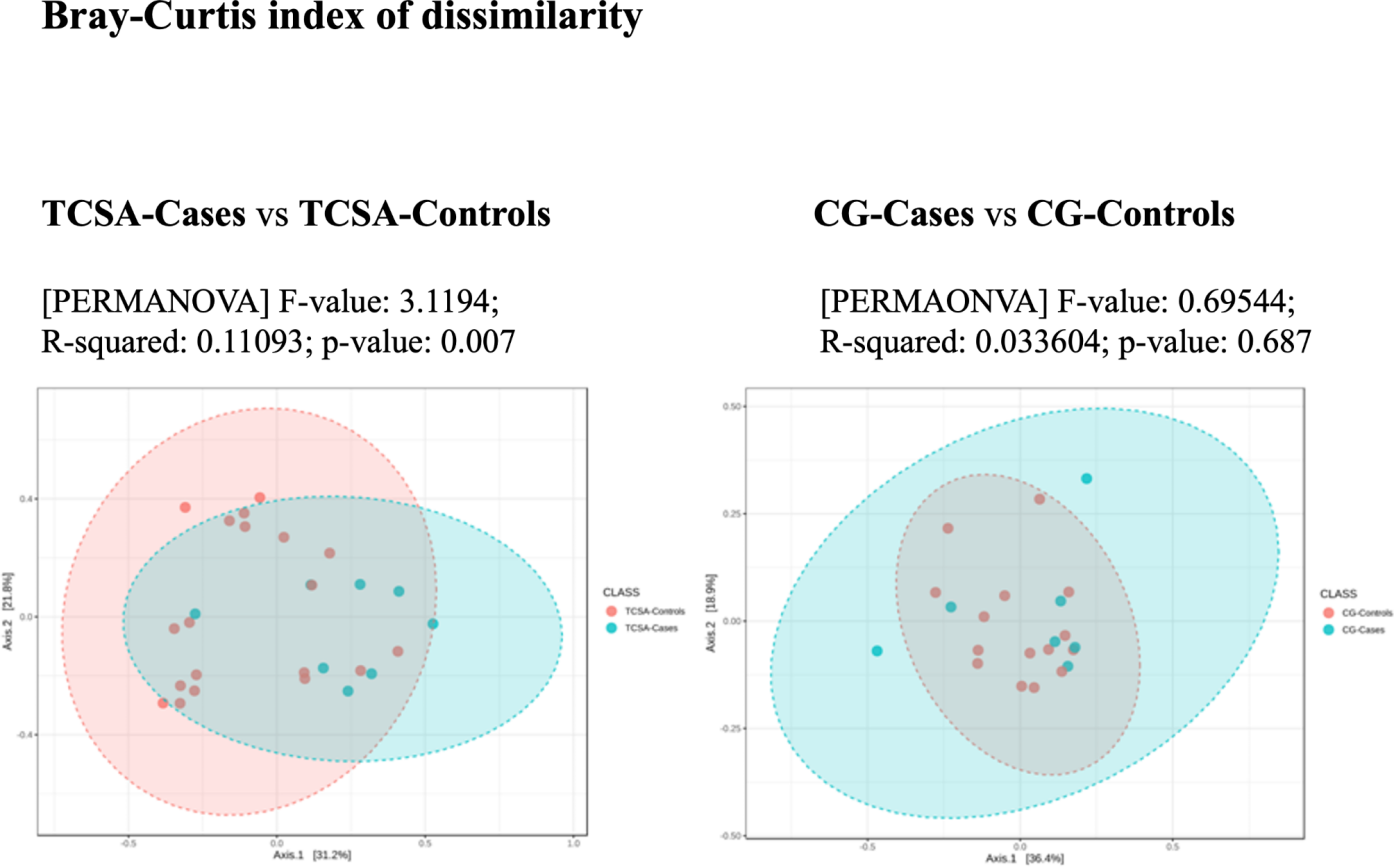
- Bray-Curtis index to compare Cases and Controls or Caregiver-Cases and Caregiver-Controls. The PerMANOVA F-value is the test statistic used in this analysis to determine if there is a significant difference in community composition between groups. A high F-value indicates a big difference between the groups being compared, while a low F-value suggests that the groups are similar. A higher R-squared value is a strong predictor of differences between the groups. In summary, a F-value of 3.1194, R-squared of 0.11093, and a p-value of 0.007 found for TCSA patients microbiomes (Cases versus Controls on the left-hand side) suggest that there is a significant difference between the groups being compared. The F-value of 0.69544, R-squared of 0.033604, and a p-value of 0.687 typical of Caregiver comparisons suggest that there is no significant difference between Caregiver-Cases and Caregiver-Controls. Numbers inside square brackets indicate the number of individuals in each group

We could not identify differences in alpha and beta diversity of microbiome belonging to Caregiver-Cases and Caregiver-Controls. The microbiome of Cases showed a lower value for all alpha diversity indexes (Chao1, Shannon, and Simpson) compared to Controls. The differences proved to be statistically significant for Shannon index (*p = 0.0158*). When coming to beta diversity, the microbiome (F-value: 3.1194; R-squared: 0.11093; *p-value = 0.007*) had compositions that turned out to be significantly different among the two populations (Fig. 4).

### Supervised analysis and relative abundance analysis

**Fig. 5:**
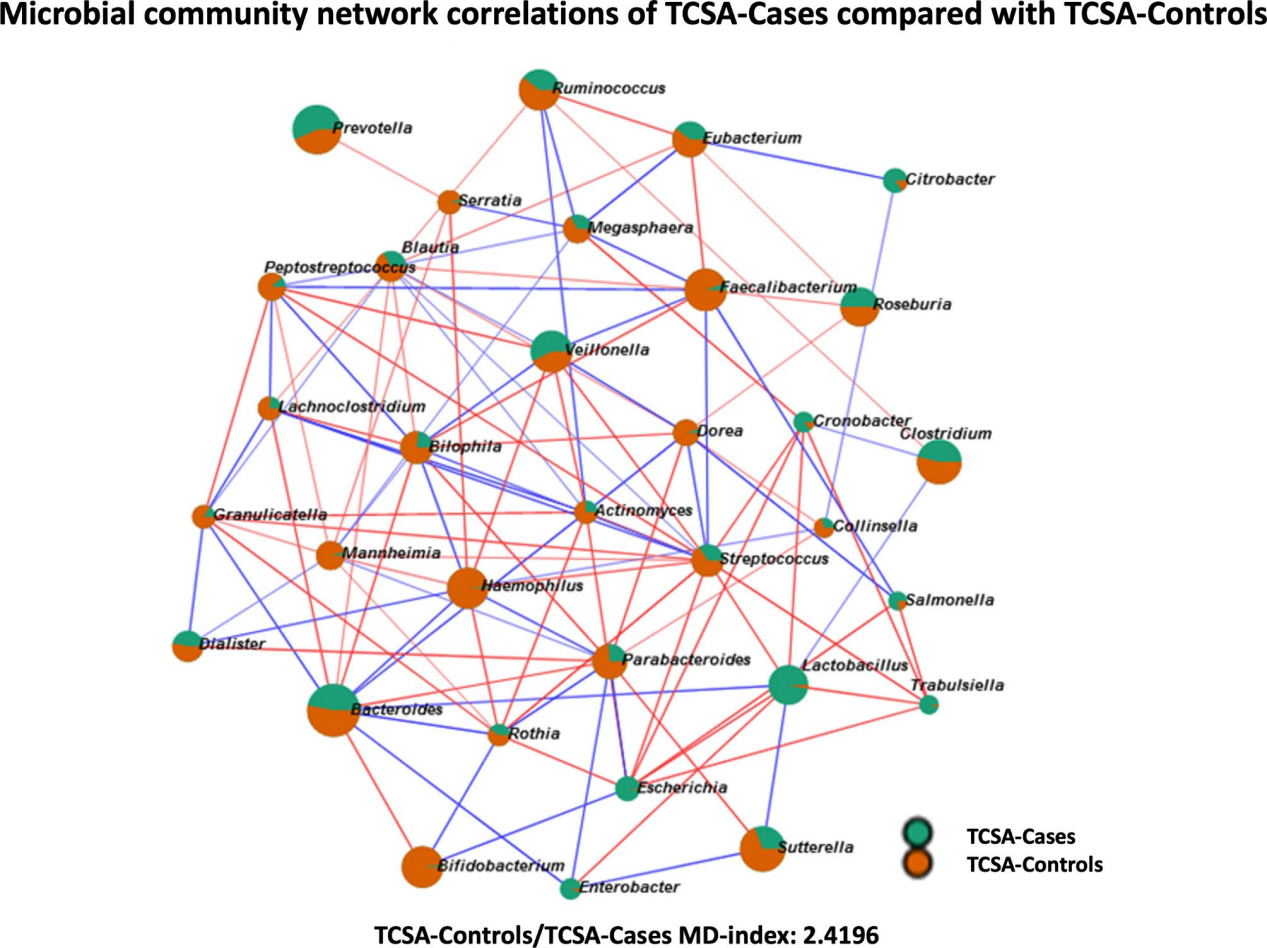
**-** Fecal microbiomes of Cases compared to Controls. Each node represents genera that were differently colored based on the preferential abundance in Cases (green) or Controls (orange). Red lines connecting nodes indicate a positive correlation between taxa, while blue lines denote a negative one. MD-index represents the dysbiosis index of Controls over Cases and indicates a marked dysbiosis with loss of genera abundances in TCSA. The network was computed for *p ≤ 0.05*, and the correlation threshold was set at 0.3

The *Gammaproteobacteria* descendants *Enterobacter*, *Escherichia coli*, and members of the *Firmicutes* phylum, like *Lactobacillus*, were found to be more abundant in Cases (Supplementary Table 2). The analysis showed that Cases are typically enriched by *Enterobacteriaceae*. Among them, *Escherichia co*li and *Trabulsiella odontotermitis* species are the more prominent microorganisms in Cases (Supplementary Table 3). These data were also confirmed by the microbial community network analysis that showed different genera of *Gammaproteobacteria* among the ones higher in Cases (Fig. 5). The comparison of Caregiver-Cases and Caregiver-Controls revealed differences in very few taxa (Supplementary Table 4), as was suggested by the beta diversity Bray-Curtis index analysis that indicates a close relationship among the taxa present in the two groups of Care Givers populations (Fig. 4).

### Multivariate clustering methodology (WGCNA)

Six variables correlated with Cases and i12 with Controls (Supplementary Tables 5 and 6). The index of chronic gut inflammation evaluated by FC values showed a positive correlation with the abundance of pro-inflammatory taxa such as *Enterobacteriaceae*, *Cronobacter*, *Escherichia*, *Shigella*, and *Escherichia coli*. These data demonstrated the usefulness of the multivariate clustering methodology used.

### PICRUSt2 analysis

The inferred metabolic pathways analysis using PICRUSt2 [41] in the KEGG database [42] showed statistical significance in Cases compared with Controls. Eight-Hundred-and-Thirty pathways were statistically associated with Cases. Among these pathways, 492 were also statistically confirmed by the LEfSe test (Supplementary Table 7a). Controls were associated to 11 ko items, seven of those found statistically significant with both DESeq2 and EdgeR and 3 with all four tests applied (Supplementary Table 7b). Cases were characterized by pathways related to Biofilm formation, virulence, and cellular adhesion. Type 1 pili were responsible for the attachment, invasion, and establishment of biofilms containing the FimH adhesin responsible for bacteria binding to the host through the mannose-containing receptors [43]. More Type IV pili were known to promote adhesion to human cells and tissues, which thus facilitates persistent infections [44]. In addition, Curli fibers involved in cell adhesion were known to stimulate host inflammatory response [45].

### Drop out patients

Patients who were dropped out had a male to female ratio of 2.33:1. In particular, those who did not agree to undergo retrograde ileoscopy were enrolled between 4 and 12 years after the pull-through and were scheduled for endoscopy due to the presence of increased FC in two (270 and 235 µg/l, respectively) and positive OFB in one (normal FC values).

## Discussion

To the best of our knowledge this is the first series of patients with TCSA who underwent such a comprehensive prospective assessment focusing on the impact of chronic inflammation in the long term. Even if previous reports already suggested a non-random association between IBDs and HSCR [23, 27, 30, 31, 36], our study demonstrated at least a 10-folds higher than expected incidence of IBD-like lesions. In fact, we demonstrated a prevalence of lesions ranging between 24% and 53% in patients with TCSA.

Based on the chosen methodology for selection of patients we could speculate that the real prevalence of IBD-like lesions in TCSA might be even higher. In fact, as we performed retrograde endoscopy only in patients who tested positive for FC or OFB or both, a number of patients with undiagnosed IBD-like lesions could have been excluded and did not undergo endoscopy. Based on this consideration we expect an underestimation of the true positive rate of TCSA patients carrying IBD-like lesions in our study.

Symptoms and features investigated by the questionnaire proved not to be specific as a relevant number of “healthy” TCSA individuals complained gastroenterological issues that did not significantly differ from those reported by patients with IBD-like lesions. Even so, a number of aspects were on the verge of significance (i.e. length of aganglionic small bowel involvement or poor growth status). Their potential role should be investigated in larger, multicentric studies in order to identify biological markers or risk factors for IBD-like lesions. Only need for blood transfusions proved to be significantly more frequent in Cases compared to Controls. Of note, statistical significance would be scaled back also for the need for blood transfusion when applying correction for multiple testing [46]. Anyway, as less than one into four Cases reported need for transfusion this aspect would have a weak role for an early clinical detection of IBD-like lesions. All these aspects limit the possibility to increase the predictability of chronic inflammation by matching clinical data with FC/OFB results.

Amongst all other possible risk factors for the development of chronic inflammation we could not identify any items significantly correlated with the presence of inflammation. No correlation was found between the adopted surgical procedure and the presence of inflammation. In particular, the presence of a pouch as that fashioned with the Duhamel procedure seemed not to increase the likelihood of inflammation nor did the reiterative surgical procedures (redoes), at least in our series.

Our study demonstrated that IBD-like lesions develop independently of age and time from surgery. The endoscopic appearance proved to be quite severe throughout ages ranging from scattered aphthous ulcerations to large ulcers with widespread ulcerations. These endoscopically evident lesions proved to have a caudo-cranial gradient with decreasing density of lesions moving away from the sphincters, suggesting a possible role of fecal stasis and bacterial overgrowth in triggering inflammation. This is confirmed by the metagenomic results of our study that demonstrated a reduced biodiversity along with an overabundance of P*roteobacteria* (*Enterobacteriaceae* in particular) and a reduction of *Firmicutes*, *Actinobacteria* and *Bacteroidetes* in Cases. The microbiota peculiarities observed in TCSA patients have been already reported by our group that demonstrated a composition resembling that of healthy subjects carrying an ileostomy due to different congenital or acquired issues [10]. Of note, it is known that *Proteobacteria* have a pro-inflammatory effect and that, conversely, *Bacteroidetes* may have a protective effect over inflammation [15]. As suggested by our group [15], the intestinal dysbiosis could also trigger a RET-dependent abnormal local immune response. In fact, we previously demonstrated an increased RET expression in circulating immune cells (PBMCs) of HSCR patients compared to healthy subjects [47]. We speculated that RET variants might predispose HSCR patients to HAEC, particularly in case of total colonic involvement as reported in TCSA [2, 4, 5, 10], by facilitating an uncontrolled inflammatory response towards a yet unknown pathogen. Of note, in our series the incidence of RET variants is similar in patients with TCSA with IBD-like lesions to that of TCSA without. This suggests that the above-mentioned immune dysregulation may not only depend on the presence of RET variants but also on other RET-related factors able to impair a correct immunomodulation [3].

We can hypothesize that microbiota imbalance due to bacterial overgrowth provide a slow and long-lasting effect in TCSA patients, leading, when left undisturbed, to chronic inflammation and IBD-like lesions, occurring in the medium- to long-term. In this context, environmental microbiota seems to play minimal to no effect in TCSA patients. This is suggested by the absence of significant differences when addressing the microbiota of caregivers of Cases and Controls. In fact, dysbiosis in patients occurs independently to what observed in caregivers. This aspect will be crucial when implementing prevention strategies aimed at reducing the progression towards inflammation.

It is intriguing the presence of a higher abundance of *Clostridium hiranonis* in Controls, but not in Cases. This microorganism could have a protective effect, since it is known to produce secondary bile acids that exert antibacterial activity against pathobionts such as *Escherichia coli* as shown in vitro. This effect has been related to the antibacterial activity of lithocholic acid in a canine animal model of chronic inflammatory enteropathy [48]. *Clostridium hiranonis* abundance and the level of lithocholic and deoxycholic acids, could potentially contribute to the control of imbalances in the intestinal microbial ecosystem and modulate intestinal inflammation [49, 50].

When coming to pathology our study discloses some of the most relevant aspects of IBD-like lesions observed in patients with TCSA. We could confirm that a severe inflammation involves the terminal ileum with a caudo-cranial gradient of lesions that tend to decrease moving proximally, away from the sphincters, with signs of crypt derangements like those observed in IBDs [51, 52]. The predominant eosinophilic infiltrate, intermingled with neutrophils, observed in patients with these lesions might represent a key aspect of the disease as well as a possible early biological biomarker of disease activity [53]. The caudo-cranial gradient suggests a role for fecal stasis occurring above the sphincters which acts chronically in these patients. The preponderance of EE in younger patients (only 1 into 4 patients younger than 6 years of age had a proper diagnosis of CD-like lesions detected at significantly higher age compared to EE) confirms this aspect. Based on these considerations, CD-like might represent the result of a long-lasting process. Eosinophils might colonize the lamina propria at the very beginning and aggressive neutrophilic infiltrate, basal plasmocytosis and eventually granulomas appear later when lesions become evident, resembling those observed in IBD [54]. If these aspects are confirmed, eosinophilic infiltrate could play a crucial diagnostic role, together with basal plasmocytosis, when addressing patients without obvious endoscopic appearance in order to develop early tailored prevention strategies and screening programmes.

This study has a number of limitations. (1) even if larger compared to previous reports, this series remains relatively small and with an excessively wide age-range of enrolled patients. This issue makes statistically analysis and interpretation difficult, particularly when addressing the subgroup of patients with IBD-like lesions; (2) in order to comply with local ethical committee requirements, we did not perform retrograde ileoscopy in all TCSA patients but only in those testing positive for FC and OFB. This selection bias is relevant as we cannot exclude the possibility that a number of those with negative FC/OFB screening could have mild to moderate endoscopic abnormalities or early biomarkers of inflammation thus increasing the number of false negatives. A prospective study including all TCSA patients to undergo retrograde ileoscopy is warranted to properly address this issue and subsequently implement definitive screening programs and prevention strategies; (3) the general and imprecise definition of HAEC status lacking number and severity of previous HAEC episodes. This limitation is mostly related to the weak diagnostic criteria [19, 55] and to the retrospective nature of the retrieval of this datum that makes it impossible to dig deeper on this regard.

Early diagnosis and development of prevention strategies represent key aspects for the management of such troublesome issues. Even if we selected patients for ileoscopy based on FC/OFB results, both FC and OFB proved not to be reliable enough to predict the presence of lesions. In fact, when addressing FC and OFB, we could demonstrate that both tests have a too low sensitivity to be used in clinical practice to implement screening programmes and prevention strategies. Given the high prevalence of lesions detected in our TCSA patients, we therefore suggest performing retrograde ileoscopy in all patients with TCSA regardless of the results of FC and OFB, particularly in case of need for blood transfusion in their past medical history. Capsule endoscopy or entero-MRI might play a role as complementary investigations or in case of poor compliance and non-suitability for conventional endoscopy. The frequency of endoscopic assessments and the role of FC/OFB screening in prompting endoscopy is yet to be determined.

## Conclusions

This study underlines that IBD-like lesions can occur in up to 50% of TCSA patients. As these lesions seem to be poorly predictable based on available screening tests, retrograde ileoscopic screening should be performed in all patients who underwent an ileo-anal pull-through for TCSA. The timing for the first endoscopic assessment is unclear but could reasonably range between 6 and 12 months postoperatively whereas that of subsequent endoscopic investigations is yet to be determined. Based on the results of the first investigation we could speculate that repeating endoscopy on a regular basis with yearly monitoring of FC/OFB and symptoms (rectal bleeding or need for transfusion above all) should help in early detecting most patients with lesions.

A future research plan should include endoscopic screening in all patients and broader questionnaires and severity scores to be implemented based on the results of our “preliminary experience”. All in all, our study provided useful data for a far larger, multicentric study addressing all TCSA patients to determine the exact prevalence of chronic inflammation in TCSA and to disclose the yet unsolved issues of IBD-like lesions in HSCR. The results could be even transferred to other HAEC or “conventional” IBD and the increased knowledge regarding this troublesome issue will possibly lead to specific patient-tailored treatments to serve the best for our patients.

## Materials and methods

From December 2020 to November 2022 (24 months), all patients affected by ultralong HSCR (TCSA), who were being followed-up at the Umberto Bosio Centre for Digestive Diseases, have been enrolled in a prospective cross-sectional observational study to address prevalence and salient features of patients with TCSA who experience IBD-Like lesions in postoperative period. The Ethical Committee approval was obtained with an EC code ASO.ChirPed.20.04. Project code was ULTRA-HSCR.

### Inclusion criteria

- Reliable diagnosis (possible re-evaluation by an expert pathologist).
- Total Colonic Aganglionosis (TCSA).
- Signature of the informed consent.

#### Exclusion criteria

- Unclear and/or unreliable diagnosis.
- Any form of HSCR other than TCSA (extended forms included).
- Presence of a stoma.
- Pull-through performed less than 6 months before enrollment.
- Refusal or withdrawal of the consent to participate in the study.
- Linguistic barrier.

#### Enrollment and informed consent

A detailed description of the project was delivered to all patients, their families, and their physician. In case of acceptance to participate, patients or their parents were asked to sign a specific Informed Consent. To protect personal data, pseudonymization was applied.

#### Phenotype

We collected the following data from all patients.

- Demographics (gender, date of birth) and familial history.
- Type of surgery and age at surgery.
- HAEC status according to what previously published [55].
- Screening for associated malformations or syndromes.

### Genotype

All patients underwent DNA extraction and amplification from patients’ genomic DNA of each of the 20 RET coding exons and flanking intronic sequences was followed by direct DNA Sanger sequencing of both strands using primers and conditions already reported [56, 57]. RET variants were checked in the parental pairs to assess the either de novo or inherited occurrence.

### Questionnaire

We provided to all patients and caregivers a detailed questionnaire addressing gastrointestinal symptoms, social and nutritional limitations, and drugs requirements. A total of 12 items were addressed (Supplementary Table 1).

## Inflammation screening

We chose Fecal Calprotectine (FC) and Occult Fecal Blood (OFB) to screen for inflammation [58–61]. All patients were asked to sample some stools at home and to perform FC and OFB in order to discriminate the likelihood of intestinal inflammation. In accordance to available literature, a cut-off of 100 µg/g was adopted for FC to screen the presence of activity [58–60]. All patients with either FC, OFB or both testing positive were scheduled for subsequent retrograde ileoscopy.

### Stool sampling for faecal microbiome analysis

Two collection and microbial DNA stabilization kits (Omnigene-Gut, DNA Genotek, 3000 − 500 Palladium Drive, Ottawa, ON, Canada, K2V 1C2) have been sent to all enrolled patients to collect some stools both of patients and caregivers the same day of FC and OFB for faecal microbiome analysis.

### Faecal microbiome analysis

DNA extraction from fecal samples was performed as reported [62] and was used for the 16 S amplification reaction performed with Ion 16 S™ Metagenomics Kit (Thermo-Fisher Scientific). This method allows the PCR-amplification of 7 out of 9 informative 16 S (V2, V4, V8, V3, V6-7, V9) polymorphic regions [63]. Then up to 16 differently bar-coded libraries were automatically loaded onto an Ion-520-chip by the Ion-Chef and sequenced by the GeneStudio-S5-system (Thermo-Fisher Scientific). Data analysis was performed with the Ion-Reporter™ suite (v 5.18.4.0) using the curated-Greengenes (v13.5) and the MicroSEQ ID 16 S-rRNA reference library (v2013.1) databases using standard parameters.

#### Endoscopy and Histology

All patients who tested positive to either FC or OFB or both were scheduled for retrograde ileoscopy that was performed under general anaesthesia (mild sedation) with a pediatric 10.7 mm colonoscope for pediatric patients or in case of behavioral issues, awake with an adult 11.6 mm colonoscope for adult patients (older than 18 years of age). Retrograde ileoscopy was carried up to 70 centimeters from the anal verge. Biopsies were taken in all patients (regardless of endoscopic appearance) during scope removal. We sampled the mucosa with and without macroscopic signs of inflammation.

Endoscopic biopsies, oriented on acetate cellulose filters (Bio-Optica, Milan, Italy), were paraffin embedded and section of 4 μm were cut and stained with Hematoxylin and Eosin. The slides were assessed by two experienced pathologists (LG and SC) and subsequently blindly reviewed by two further expert pathologists (AP and VV). We used the criteria by Patil and Odze to determine severity and activity of inflammation [51]. In particular, eosinophil count was obtained from one high-power (X40 magnification) field, in accordance to what previously published [52, 64, 65]. Based on the above-mentioned criteria patients were arbitrary categorized as follows.

- **Case** (presence of signs of either chronicity or activity of inflammation).
  - **Crohn-like (CD-like)** – this is a combination of neutrophilic and eosinophilic infiltrates in lamina propria and superficial epithelium, various degrees of atrophy, basal plasmocytosis, and granulomas with the mandatory presence of basal plasmocytosis.
  - **Eosinophilic Enteritis (EE)’** – this is a combination of neutrophilic and eosinophilic infiltrates without basal plasmocytosis nor granulomas. We considered EE each patient with an eosinophilic count higher than 50 elements for each X40 magnification field.
- **Control** (absence of signs of either chronicity or activity of inflammation).

#### Disease activity and severity of lesions

To address disease activity, we adopted the Pediatric Crohn Disease Activity Index (PCDAI), specifically developed for CD in children [66]. In order to implement this score, we assessed growth parameters (height and weight) and laboratory tests (full blood count, erythrocyte sedimentation rate, and albumin levels). Given the wide age-range at inclusion in the study we resorted to Z-scores and BMI in prepubertal and postpubertal patients, respectively.

Similarly, to score the severity of endoscopic lesions, we resorted to the Simplified Endoscopic Mucosal Assessment for Crohn’s Disease (SEMA-CD) [67] based on the endoscopic similarities with CD, on literature data, and on the ileal localization of the lesions.

## Drop out patients

Relevant features of patients who were dropped out during the study has been provided in order to address all potential confounders or inclusion bias.

## Statistical and data analysis

Data were analyzed using descriptive statistics and inferential statistics. Regarding the descriptive statistics, the quantitative variables (continuous and discrete) were reported using median and range. Qualitative or categorical variables were reported using frequency with relative percentage. 95% CI was reported when appropriate. The comparison between the two subgroups of our population was made using the Kruskal-Wallis test for quantitative variables and Fisher’s exact test for qualitative/categorical variables. A *p* value *< 0.05* was considered statistically significant.

Microbiome compositional/functional profiling and comparative analysis were performed with MicrobiomeAnalyst 2 web-tools [68, 69]. All the resulting *p-values* have been adjusted to correct for multiple hypotheses, using Benjamini and Hochberg false discovery rate (FDR < 0.05). In Cases vs. Controls comparison, the MD-index was computed as the logarithm of the summation of the abundances of all the microorganisms that increased over controls divided by the total sum of the quantities of microorganisms that decreased it. The weighted correlation network analysis (WGCNA) was used to verify correlations of taxa with the clinical parameters characterizing Cases and Controls [70]. The color code in the WGCNA heatmaps indicate respectively the identified positive (red) or negative (blue) Pearson correlations. The metagenome functional content was predicted using PICRUSt2 [41], to get the KEGG Orthology (KO) terms Table [42] and the inferred MetaCyc pathways [71]. These data were analyzed with the Shotgun-data-profiling module of MicrobiomeAnalyst. The abundance of the pathways between the groups was analyzed with the unpaired Wilcoxon test and the results were integrated *a posteriori* with the results obtained from the Shotgun-data-profiling module of MicrobiomeAnalyst.

### Electronic supplementary material

Below is the link to the electronic supplementary material.

Supplementary Material 1

Supplementary Material 2

Supplementary Material 3

Supplementary Material 4

Supplementary Material 5

Supplementary Material 6

Supplementary Material 7

Supplementary Material 8

Supplementary Material 9

## Data Availability

The dataset supporting the conclusions of this article is openly available in the Short Read Archive (SRA), Sequence Read Archive NCBI-NIH. SRA BioProject ID: PRJNA952921. The records will be accessible after the release date at the following link https://www.ncbi.nlm.nih.gov/sra/PRJNA952921.
