# Supplementary material for: A unicentric cross-sectional observational study on chronic intestinal inflammation in total colonic aganglionosis: beware of an underestimated condition
Source: Orphanet J Rare Dis. 2023 Oct 27;18:339. doi: 10.1186/s13023-023-02958-1 (PMC10612252; doi:10.1186/s13023-023-02958-1)
Supplement: Supplementary file 1 — Supplementary Material 1 [file 13023_2023_2958_MOESM1_ESM.docx]

**Supplementary Table 5: Multivariate clustering of microbiome of TCSA-Cases and clinical variable**

| **last month Anti Inflammatory drug administration** | **syndrome or anomalies** | **Iron administration** | **daily life limitation** | **HSCR familiarity** | **fecal occult blood test** |
| --- | --- | --- | --- | --- | --- |
| **Cluster 2** | **Cluster 5** | **Cluster 1** | **Cluster 5** |  |  |
| Pearson corr. = 0.99,  p-value = 2.66e-05 | Pearson corr. = 1,  p-value = 5.75e-07 | Pearson corr. = 0.74,  p-value = 0.04 | Pearson corr. = -0.80,  p-value = 0.02 |  |  |
| **Pearson positive correlation last month Anti Inflammatory drug administration** | **positive correlation with syndrome or anomalies** | **positive correlation with Iron drug administration** | **negative correlation with daily life limitation** | **correlation with HSCR familiarity** | **correlation with fecal occult blood test** |
| *Bacteroidaceae,* | *Enterococcaceae,* | *Francisellaceae,* | *Enterococcaceae,* |  |  |
| *Bifidobacteriaceae,* | *Erythrobacteraceae,* | *Erysipelotrichaceae,* | *Enterobacteriaceae* |  |  |
| *Burkholderiaceae,* | *Lachnospiraceae,* | *Bifidobacteriaceae,* |  |  |  |
| *Chromatiaceae,* | *Oceanospirillaceae,* | *Pasteurellaceae* |  |  |  |
| *Clostridiaceae,* | *Veillonellaceae* |  |  |  |  |
| *Clostridiales Family XI Incertae Sedis,* |  |  |  |  |  |
| *Clostridiales Family XIII Incertae Sedis,* |  |  |  |  |  |
| *Coriobacteriaceae,* |  |  |  |  |  |
| *Defluviitaleaceae,* |  |  |  |  |  |
| *Enterobacteriaceae,* |  |  |  |  |  |
| *Erysipelotrichaceae,* |  |  |  |  |  |
| *Eubacteriaceae,* |  |  |  |  |  |
| *Francisellaceae,* |  |  |  |  |  |
| *Pasteurellaceae,* |  |  |  |  |  |
| *Pseudomonadaceae,* |  |  |  |  |  |
| *unclassified Clostridiales,* |  |  |  |  |  |
| *Vibrionaceae* |  |  |  |  |  |
|  |  |  |  |  |  |
| **Cluster 2** | **Cluster 7** |  |  | **Cluster 4** | **Cluster 5** |
| Pearsoncorr. = 0.99,  p-value = 2.29e-05 | Pearson corr. = 1,  p-value = 1.87e-06 |  |  | Pearsoncorr. = 0.77,  p-value = 0.03 | Pearsoncorr. = 0.73,  p-value = 0.04 |
| **Pearson positive correlation last month Anti Inflammatory drug administration** | **positive correlation with syndrome or anomalies** | **correlation with Iron drug administration** | **correlation with daily life limitation** | **positive correlation with HSCR familiarity** | **positive correlation with fecal occult blood test** |
| *Allisonella,* | *Bulleidia,* |  |  | *Collinsella,* | *Actinomyces,* |
| *Bifidobacterium,* | *Citrobacter,* |  |  | *Cronobacter,* | *Atopobium* |
| *Butyricicoccus,* | *Dialister,* |  |  | *Howardella,* |  |
| *Clostridium,* | *Eggerthella,* |  |  | *Megasphaera,* |  |
| *Flavonifractor,* | *Enterobacter,* |  |  | *Parabacteroides* |  |
| *Haemophilus,* | *Enterococcus,* |  |  |  |  |
| *Mannheimia,* | *Gibbsiella,* |  |  |  |  |
| *Morganella,* | *Leclercia,* |  |  |  |  |
| *Peptostreptococcus,* | *Mangrovibacter,* |  |  |  |  |
| *Photobacterium,* | *Neisseria,* |  |  |  |  |
| *Proteus,* | *Pluralibacter,* |  |  |  |  |
| *Serratia,* | *Roseburia,* |  |  |  |  |
| *Trabulsiella,* | *Ruminococcus,* |  |  |  |  |
| *Xenorhabdus* | *Salmonella* |  |  |  |  |
|  | *Shigella,* |  |  |  |  |
|  | *Veillonella,* |  |  |  |  |
|  | *Yokenella* |  |  |  |  |
|  |  |  |  |  |  |
| **Cluster 7** | **Cluster 2** | **Cluster 8** |  |  |  |
| Pearsoncorr. = 1,  p-value = 7.36 e-08 | Pearson corr. = 1,  p-value = 7.36 e-08 | Pearson corr. = 0.71,  p-value = 0.050 |  |  |  |
| **Pearson positive correlation last month Anti Inflammatory drug administration** | **positive correlation with syndrome or anomalies** | **positive correlation with Iron drug administration** | **correlation with daily life limitation** | **correlation with HSCR familiarity** | **correlation with fecal occult blood test** |
| *Allisonella histaminiformans,* | *Bulleidia moorei,* | *Actinomyces odontolyticus,* |  |  |  |
| *Bacteroides rodentium,* | *Citrobacter farmeri,* | *Bacteroides caccae,* |  |  |  |
| *Bacteroides uniformis,* | *Citrobacter murliniae,* | *Bacteroides fragilis,* |  |  |  |
| *Bifidobacterium bifidum,* | *Citrobacter werkmanii,* | *Bacteroides ovatus,* |  |  |  |
| *Bifidobacterium longum,* | *Clostridium perfringens,* | *Bifidobacterium pseudocatenulatum,* |  |  |  |
| *Blautia sp.,* | *Dialister pneumosintes,* | *Blautia wexlerae,* |  |  |  |
| *Butyricicoccus pullicaecorum,* | *Eggerthella lenta,* | *Collinsella aerofaciens,* |  |  |  |
| *Clostridium aldenense,* | *Enterobacter asburiae,* | *Coprococcus comes,* |  |  |  |
| *Clostridium asparagiforme,* | *Enterobacter sp.,* | *Cronobacter sakazakii,* |  |  |  |
| *Clostridium bolteae,* | *Escherichia albertii,* | *Cronobacter turicensis,* |  |  |  |
| *Clostridium butyricum,* | *Fusobacterium periodonticum,* | *Enterococcus cecorum,* |  |  |  |
| *Clostridium celatum,* | *Gibbsiella quercinecans,* | *Escherichia/Shigella coli/dysenteriae,* |  |  |  |
| *Clostridium citroniae,* | *Kluyvera georgiana,* | *Megasphaera sp.,* |  |  |  |
| *Clostridium clostridioforme,* | *Lactobacillus mucosae,* | *Parabacteroides merdae,* |  |  |  |
| *Clostridium cocleatum,* | *Lactobacillus rogosae,* | *Salmonella enterica,* |  |  |  |
| *Clostridium disporicum,* | *Leclercia adecarboxylata,* | *Streptococcus alactolyticus,* |  |  |  |
| *Clostridium hathewayi,* | *Mangrovibacter plantisponsor,* | *Trabulsiella odontotermitis,* |  |  |  |
| *Clostridium neonatale,* | *Megasphaera micronuciformis,* | *Veillonella atypica,* |  |  |  |
| *Clostridium nexile,* | *Neisseria elongata,* | *Veillonella denticariosi,* |  |  |  |
| *Clostridium ramosum,* | *Pluralibacter pyrinus,* | *Veillonella dispar* |  |  |  |
| *Clostridium saccharoperbutylacetonicum,* | *Roseburia faecis,* |  |  |  |  |
| *Clostridium symbiosum,* | *Roseburia hominis,* |  |  |  |  |
| *Eubacterium eligens,* | *Ruminococcus faecis,* |  |  |  |  |
| *Eubacterium ramulus,* | *Ruminococcus gnavus,* |  |  |  |  |
| *Flavonifractor plautii,* | *Shigella sonnei,* |  |  |  |  |
| *Fusobacterium nucleatum,* | *Veillonella alcalescens,* |  |  |  |  |
| *Fusobacterium sp.,* | *Veillonella caviae,* |  |  |  |  |
| *Haemophilus parainfluenzae,* | *Veillonella parvula,* |  |  |  |  |
| *Mannheimia varigena,* | *Veillonella rodentium,* |  |  |  |  |
| *Morganella morganii,* | *Veillonella rogosae,* |  |  |  |  |
| *Oribacterium sinus,* | *Veillonella tobetsuensis,* |  |  |  |  |
| *Peptostreptococcus stomatis,* | *Yokenella regensburgei* |  |  |  |  |
| *Proteus hauseri,* |  |  |  |  |  |
| *Proteus penneri,* |  |  |  |  |  |
| *Proteus vulgaris,* |  |  |  |  |  |
| *Sutterella stercoricanis,* |  |  |  |  |  |
| *Xenorhabdus bovienii* |  |  |  |  |  |

The categorical variables analyzed were the presence for: "Inflammatory drug administration", "syndrome or anomalies", "Iron drug administration", "daily life limitation", "HSCR familiarity". In red are indicated the negatively correlated variables. A Pearson positive or negative coefficient p-values were adjusted for multiple comparisons with False Discovery Rate (FDR).
