# Supplementary material for: A unicentric cross-sectional observational study on chronic intestinal inflammation in total colonic aganglionosis: beware of an underestimated condition
Source: Orphanet J Rare Dis. 2023 Oct 27;18:339. doi: 10.1186/s13023-023-02958-1 (PMC10612252; doi:10.1186/s13023-023-02958-1)
Supplement: Supplementary file 2 — Supplementary Material 2 [file 13023_2023_2958_MOESM2_ESM.docx]

**Supplementary Table 6: Multivariate clustering of microbiome of TCSA-Controls and clinical variable**

| **last month Anti Inflammatory drug administration** | **age at recruitment** | **previous HAEC** | **Calprotectin values** | **fecal occult blood test** | **HSCR Familiarity** | **Iron drug administration** | **use antibiotics in the last month** | **Male sex** | **daily life limitation** | **Duhamel pull through method** | **syndrome or anomalies** |
| --- | --- | --- | --- | --- | --- | --- | --- | --- | --- | --- | --- |
| **Cluster 10** | **Cluster 2** |  | **Cluster 4** |  |  | **Cluster 6** | **Cluster 3** | **Cluster 2** | **Cluster 2** | **Cluster 3** |  |
| Pearson corr. = 0.99,  p-value = 1.42e-14 | Pearson corr. = 0.72,  p-value = 5e-04 |  | Pearson corr. = 0.56,  p-value = 0.01 |  |  | Pearson corr. = 0.55,  p-value = 0.01 | Pearson corr. = 0.62,  p-value = 0.004 | Pearson corr. = 0.47,  p-value = 0.04 | Pearson corr. = 0.60,  p-value = 0.007 | Pearson corr. = 0.62,  p-value = 0.03 |  |
| **Pearson positive correlation with last month Anti-inflammatory administration** | **Pearson positive correlation with age at recruitment** | **Pearson correlation with previous HAEC** | **Pearson positive correlation with Calprotectin values** | **Pearson correlation with fecal occult blood test** | **Pearson correlation with HSCR familiarity** | **Pearson positive correlation with Iron drug administration** | **Pearson positive correlation with the use of antibiotics in the last month** | **Pearson positive correlation with Male sex** | **Pearson positive correlation with daily limitation** | **Pearson positive correlation with Duhamel pull through method** | **Pearson correlation with syndrome or anomalies** |
| *Bdellovibrionaceae,* | *Enterococcaceae,* |  | *Enterobacteriaceae,* |  |  | *Chromatiaceae,* | *Bacteroidaceae,* | *Enterococcaceae,* | *Enterococcaceae,* | *Bacteroidaceae,* |  |
| *Caulobacteraceae,* | *Hydrogenophilaceae,* |  | *Shewanellaceae* |  |  | *Defluviitaleaceae,* | *Beijerinckiaceae,* | *Erysipelotrichaceae,* | *Erysipelotrichaceae,* | *Beijerinckiaceae,* |  |
| *Clostridiaceae,* | *Porphyromonadaceae* |  |  |  |  | *Lachnospiraceae,* | *Cytophagaceae,* | *Enterobacteriaceae,* | *Enterobacteriaceae,* | *Cytophagaceae,* |  |
| *Geodermatophilaceae,* |  |  |  |  |  | *Lactobacillaceae,* | *Deinococcaceae,* | *Rhodobacteraceae* | *Rhodobacteraceae* | *Deinococcaceae,* |  |
| *Opitutaceae,* |  |  |  |  |  | *Pseudanabaenaceae,* | *Desulfovibrionaceae,* |  |  | *Desulfovibrionaceae,* |  |
| *Pseudonocardiaceae,* |  |  |  |  |  | *Vibrionaceae* | *Fusobacteriaceae,* |  |  | *Fusobacteriaceae,* |  |
| *Rhodobacteraceae,* |  |  |  |  |  |  | *unclassified Clostridiales* |  |  | *unclassified Clostridiales* |  |
| *Sutterellaceae* |  |  |  |  |  |  |  |  |  |  |  |
|  |  |  |  |  |  |  |  |  |  |  |  |
| **Cluster 16** | **Cluster 4** | **Cluster 1** | **Cluster 7** | **Cluster 8** | **Cluster 9** | **Cluster 9** | **Cluster 3** |  |  |  | **Cluster 14** |
| Pearson corr. = 0.99,  p-value = 3e-17 | Pearson corr. = 0.67,  p-value = 0.002 | Pearson corr. = 0.66,  p-value = 0.002 | Pearson corr. = 0.60,  p-value = 0.007 | Pearson corr. = 0.67,  p-value = 0.002 | Pearson corr. = 0.74,  p-value = 3e-04 | Pearson corr. = 0.65,  p-value = 0.002 | Pearson corr. = 0.65,  p-value = 0.003 |  |  |  | Pearson corr. = 0.54,  p-value = 0.02 |
| **Pearson positive correlation last month Anti Inflammatory drug administration** | **Pearson positive correlation with age at recruitment** | **Pearson positive correlation with previous HAEC** | **Pearson positive correlation with Calprotectin values** | **Pearson positive correlation with fecal occult blood test** | **Pearson positive correlation with HSCR familiarity** | **Pearson positive correlation with Iron drug administration** | **Pearson positive correlation with the use of antibiotics in the last month** | **Pearson correlation with Male sex** | **Pearson correlation with daily limitation** | **Pearson Duhamel pull through method** | **Pearson positive correlation with syndrome or anomalies** |
| *Actinomycetospora,* | *Dialister,* | *Anaerofustis,* | *Cronobacter,* | *Pediococcus,* | *Citrobacter,* | *Citrobacter,* | *Acinetobacter,* |  |  |  | *Mannheimia,* |
| *Blastococcus,* | *Enterococcus,* | *Eggerthella,* | *Escherichia,* | *Veillonella* | *Klebsiella,* | *Klebsiella,* | *Bilophila,* |  |  |  | *Terrisporobacter,* |
| *Clostridium,* | *Parabacteroides* | *Flavonifractor,* | *Trabulsiella* |  | *Megasphaera* | *Megasphaera* | *Butyricimonas,* |  |  |  | *Turicibacter* |
| *Flavobacterium,* |  | *Tyzzerella* |  |  |  |  | *Coprobacillus,* |  |  |  |  |
| *Opitutus,* |  |  |  |  |  |  | *Fusobacterium,* |  |  |  |  |
| *Paracoccus,* |  |  |  |  |  |  | *Holdemania,* |  |  |  |  |
| *Phenylobacterium,* |  |  |  |  |  |  | *Lachnoclostridium,* |  |  |  |  |
| *Shigella,* |  |  |  |  |  |  | *Parasutterella,* |  |  |  |  |
| *Sutterella* |  |  |  |  |  |  | *Subdoligranulum* |  |  |  |  |
|  |  |  |  |  |  |  |  |  |  |  |  |
| **Cluster 16** | **Cluster 13** | **Cluster 11** | **Cluster 6** | **Cluster 1** | **Cluster 10** | **Cluster 2** | **Cluster 14** |  |  |  | **Cluster 17** |
| Pearson corr. = 0.99,  p-value =3e-17 | Pearson corr. = 0.69,  p-value = 0.001 | Pearson corr. = 0.63,  p-value =0.004 | Pearson corr. = 0.58,  p-value = 0.009 | Pearson corr. = 0.7,  p-value = 9e-04 | Pearson corr. = 0.79,  p-value = 7e-05 | Pearson corr. = 0.67,  p-value = 0.002 | Pearson corr. = 0.67,  p-value = 0.002 |  |  |  | Pearson corr. = 0.48,  p-value = 0.04 |
| **Pearson positive correlation last month Anti Inflammatory drug administration** | **Pearson positive correlation with age at recruitment** | **Pearson positive correlation with previous HAEC** | **Pearson positive correlation with Calprotectin values** | **Pearson positive correlation with fecal occult blood test** | **Pearson positive correlation with HSCR familiarity** | **Pearson positive correlation with Iron drug administration** | **Pearson positive correlation with the use of antibiotics in the last month** | **Pearson correlation with Male sex** | **Pearson correlation with daily limitation** | **Pearson Duhamel pull through method** | **Pearson positive correlation with syndrome or anomalies** |
| *Blastococcus endophyticus,* | *Bacteroides ovatus,* | *Bacteroides thetaiotaomicron,* | *Cronobacter turicensis,* | *Bifidobacterium breve,* | *Bacteroides plebeius,* | *Bacteroides plebeius,* | *Anaerostipes caccae,* |  |  |  | *Bacteroides faecis,* |
| *Clostridium butyricum,* | *Clostridium asparagiforme,* | *Clostridium innocuum,* | *Escherichia coli,* | *Bifidobacterium stercoris,* | *Klebsiella variicola,* | *Klebsiella variicola,* | *Bacteroides caccae,* |  |  |  | *Bacteroides vulgatus,* |
| *Clostridium vincentii,* | *Clostridium clostridioforme,* | *Clostridium lactatifermentans,* | *Escherichia vulneris,* | *Lactobacillus fermentum,* | *Megasphaera sp.* | *Megasphaera sp.* | *Bacteroides cellulosilyticus,* |  |  |  | *Citrobacter freundii,* |
| *Flavobacterium myungsuense,* | *Clostridium hathewayi,* | *Clostridium lavalense,* | *Escherichia/Shigella coli/dysenteriae,* | *Lactobacillus johnsonii,* |  |  | *Bacteroides finegoldii,* |  |  |  | *Clostridium glycolicum,* |
| *Flavobacterium nitratireducens,* | *Clostridium scindens,* | *Eggerthella lenta,* | *Shigella flexneri,* | *Lactobacillus kitasatonis,* |  |  | *Bacteroides intestinalis,* |  |  |  | *Eubacterium hallii,* |
| *Shigella sonnei,* | *Clostridium symbiosum,* | *Flavonifractor plautii,* | *Trabulsiella odontotermitis* | *Lactobacillus paracasei,* |  |  | *Bacteroides nordii,* |  |  |  | *Mannheimia varigena,* |
| *Streptococcus pasteurianus,* | *Coprococcus comes,* | *Phascolarctobacterium sp.* |  | *Neisseria subflava,* |  |  | *Bacteroides oleiciplenus,* |  |  |  | *Neisseria perflava,* |
| *Sutterella parvirubra,* | *Dialister pneumosintes,* |  |  | *Pediococcus acidilactici,* |  |  | *Bacteroides salyersiae,* |  |  |  | *Oribacterium sp.,* |
| *Sutterella stercoricanis* | *Dialister propionicifaciens,* |  |  | *Pediococcus lolii,* |  |  | *Bacteroides stercorirosoris,* |  |  |  | *Porphyromonas endodontalis,* |
|  | *Enterococcus avium,* |  |  | *Streptococcus thermophilus,* |  |  | *Bilophila wadsworthia,* |  |  |  | *Ruminococcus torques,* |
|  | *Fusobacterium genomosp.,* |  |  | *Veillonella atypica,* |  |  | *Blautia producta,* |  |  |  | *Streptococcus caballi,* |
|  | *Fusobacterium nucleatum,* |  |  | *Veillonella dispar* |  |  | *Butyricimonas sp.,* |  |  |  | *Turicibacter sanguinis,* |
|  | *Fusobacterium sp.,* |  |  |  |  |  | *Clostridium citroniae,* |  |  |  | *Veillonella ratti* |
|  | *Lachnoclostridium hathewayi,* |  |  |  |  |  | *Clostridium nexile,* |  |  |  |  |
|  | *Parabacteroides distasonis,* |  |  |  |  |  | *Coprobacillus cateniformis,* |  |  |  |  |
|  | *Parabacteroides merdae* |  |  |  |  |  | *Dialister invisus,* |  |  |  |  |
|  |  |  |  |  |  |  | *Eubacterium dolichum,* |  |  |  |  |
|  |  |  |  |  |  |  | *Fusobacterium mortiferum,* |  |  |  |  |
|  |  |  |  |  |  |  | *Fusobacterium varium,* |  |  |  |  |
|  |  |  |  |  |  |  | *Holdemania filiformis,* |  |  |  |  |
|  |  |  |  |  |  |  | *Lachnoclostridium clostridioforme,* |  |  |  |  |
|  |  |  |  |  |  |  | *Parabacteroides johnsonii,* |  |  |  |  |
|  |  |  |  |  |  |  | *Parasutterella excrementihominis,* |  |  |  |  |
|  |  |  |  |  |  |  | *Subdoligranulum sp.* |  |  |  |  |

The categorical variables analyzed were the presence for: "Inflammatory drug administration", "previous reported HAEC", "HSCR familiarity", "Iron drug administration", "use of antibiotics in the previous month", "sex", "daily life limitation", "Duhamel pull through method", "syndrome or anomalies". In red are indicated the negatively correlated variables. A Pearson positive or negative coefficient p-values were adjusted for multiple comparisons with False Discovery Rate (FDR).
