# Supplementary material for: A unicentric cross-sectional observational study on chronic intestinal inflammation in total colonic aganglionosis: beware of an underestimated condition
Source: Orphanet J Rare Dis. 2023 Oct 27;18:339. doi: 10.1186/s13023-023-02958-1 (PMC10612252; doi:10.1186/s13023-023-02958-1)
Supplement: Supplementary file 3 — Supplementary Material 3 [file 13023_2023_2958_MOESM3_ESM.docx]

**Supplementary Table 7a**

| **KO** | **Gene Symbol** | **Gene Name** | **Wilcoxon_group** | **KO Brite group** | **LEfSe_group** | **DESeq2_group** | **EdgeR_group** |
| --- | --- | --- | --- | --- | --- | --- | --- |
| K00135 | gabD | succinate-semialdehyde dehydrogenase / glutarate-semialdehyde dehydrogenase [EC:1.2.1.16 1.2.1.79 1.2.1.20] | TCSA-Cases | ko01000 Enzymes | TCSA-Cases | None | None |
| K07232 | CHAC, chaC | glutathione-specific gamma-glutamylcyclotransferase [EC:4.3.2.7] | TCSA-Cases | ko01000 Enzymes | TCSA-Cases | None | None |
| K07644 | cusS, copS, silS | two-component system, OmpR family, heavy metal sensor histidine kinase CusS [EC:2.7.13.3] | TCSA-Cases | ko01000 Enzymes | TCSA-Cases | None | None |
| K07665 | cusR, copR, silR | two-component system, OmpR family, copper resistance phosphate regulon response regulator CusR | TCSA-Cases | ko02022 Two-component | TCSA-Cases | None | None |
| K10804 | tesA | acyl-CoA thioesterase I [EC:3.1.2.- 3.1.2.2 3.1.1.2 3.1.1.5] | TCSA-Cases | ko01000 Enzymes | TCSA-Cases | None | None |
| K03414 | cheZ | chemotaxis protein CheZ | TCSA-Cases | ko02035 Bacterial | TCSA-Cases | None | None |
| K05520 | pfpI | protease I [EC:3.5.1.124] | TCSA-Cases | ko01000 Enzymes | TCSA-Cases | None | None |
| K05774 | phnN | ribose 1,5-bisphosphokinase [EC:2.7.4.23] | TCSA-Cases | ko01000 Enzymes | TCSA-Cases | None | None |
| K06162 | phnM | alpha-D-ribose 1-methylphosphonate 5-triphosphate diphosphatase [EC:3.6.1.63] | TCSA-Cases | ko01000 Enzymes | TCSA-Cases | None | None |
| K07774 | tctD | two-component system, OmpR family, response regulator TctD | TCSA-Cases | ko02022 Two-component | TCSA-Cases | None | None |
| K09019 | rutE | 3-hydroxypropanoate dehydrogenase [EC:1.1.1.-] | TCSA-Cases | ko01000 Enzymes | TCSA-Cases | None | None |
| K00108 | betA, CHDH | choline dehydrogenase [EC:1.1.99.1] | TCSA-Cases | ko01000 Enzymes | TCSA-Cases | None | None |
| K00285 | dadA | D-amino-acid dehydrogenase [EC:1.4.5.1] | TCSA-Cases | ko01000 Enzymes | TCSA-Cases | None | None |
| K00344 | qor, CRYZ | NADPH:quinone reductase [EC:1.6.5.5] | TCSA-Cases | ko01000 Enzymes | TCSA-Cases | None | None |
| K02773 | gatA, sgcA | galactitol PTS system EIIA component [EC:2.7.1.200] | TCSA-Cases | ko01000 Enzymes | TCSA-Cases | None | None |
| K07799 | mdtA | membrane fusion protein, multidrug efflux system | TCSA-Cases | ko02000 Transporters | TCSA-Cases | None | None |
| K11902 | impA | type VI secretion system protein ImpA | TCSA-Cases | ko02044 Secretion | TCSA-Cases | None | None |
| K12262 | cybB | superoxide oxidase [EC:1.10.3.17] | TCSA-Cases | ko01000 Enzymes | TCSA-Cases | None | None |
| K14261 | alaC | alanine-synthesizing transaminase [EC:2.6.1.-] | TCSA-Cases | ko01000 Enzymes | TCSA-Cases | None | None |
| K00381 | cysI | sulfite reductase (NADPH) hemoprotein beta-component [EC:1.8.1.2] | TCSA-Cases | ko01000 Enzymes | TCSA-Cases | None | None |
| K03098 | APOD | apolipoprotein D and lipocalin family protein | TCSA-Cases | ko04147 Exosome | TCSA-Cases | None | None |
| K03676 | grxC, GLRX, GLRX2 | glutaredoxin 3 | TCSA-Cases | ko03110 Chaperones | TCSA-Cases | None | None |
| K03776 | aer | aerotaxis receptor | TCSA-Cases | ko02035 Bacterial | TCSA-Cases | None | None |
| K07147 | msrP | methionine sulfoxide reductase catalytic subunit [EC:1.8.-.-] | TCSA-Cases | ko01000 Enzymes | TCSA-Cases | None | None |
| K07649 | tctE | two-component system, OmpR family, sensor histidine kinase TctE [EC:2.7.13.3] | TCSA-Cases | ko01000 Enzymes | TCSA-Cases | None | None |
| K10680 | nemA | N-ethylmaleimide reductase [EC:1.-.-.-] | TCSA-Cases | ko01000 Enzymes | TCSA-Cases | None | None |
| K11066 | E3.5.1.28D, amiD | N-acetylmuramoyl-L-alanine amidase [EC:3.5.1.28] | TCSA-Cases | ko01000 Enzymes | TCSA-Cases | None | None |
| K11392 | rsmF | 16S rRNA (cytosine1407-C5)-methyltransferase [EC:2.1.1.178] | TCSA-Cases | ko01000 Enzymes | TCSA-Cases | None | None |
| K11742 | mdtI | spermidine export protein MdtI | TCSA-Cases | ko02000 Transporters | TCSA-Cases | None | None |
| K11743 | mdtJ | spermidine export protein MdtJ | TCSA-Cases | ko02000 Transporters | TCSA-Cases | None | None |
| K15268 | eamA | O-acetylserine/cysteine efflux transporter | TCSA-Cases | ko02000 Transporters | TCSA-Cases | None | None |
| K16137 | nemR | TetR/AcrR family transcriptional regulator, transcriptional repressor for nem operon | TCSA-Cases | ko03000 Transcription | TCSA-Cases | None | None |
| K09945 | K09945 | uncharacterized protein | TCSA-Cases | None | TCSA-Cases | None | None |
| K07168 | K07168 | CBS domain-containing membrane protein | TCSA-Cases | None | TCSA-Cases | None | None |
| K00556 | trmH | tRNA (guanosine-2'-O-)-methyltransferase [EC:2.1.1.34] | TCSA-Cases | ko01000 Enzymes | TCSA-Cases | None | None |
| K00899 | mtnK | 5-methylthioribose kinase [EC:2.7.1.100] | TCSA-Cases | ko01000 Enzymes | TCSA-Cases | None | None |
| K01061 | E3.1.1.45 | carboxymethylenebutenolidase [EC:3.1.1.45] | TCSA-Cases | ko01000 Enzymes | TCSA-Cases | None | None |
| K01087 | otsB | trehalose 6-phosphate phosphatase [EC:3.1.3.12] | TCSA-Cases | ko01000 Enzymes | TCSA-Cases | None | None |
| K01801 | nagL | maleylpyruvate isomerase [EC:5.2.1.4] | TCSA-Cases | ko01000 Enzymes | TCSA-Cases | None | None |
| K02478 | K02478 | two-component system, LytTR family, sensor kinase [EC:2.7.13.3] | TCSA-Cases | ko01000 Enzymes | TCSA-Cases | None | None |
| K03782 | katG | catalase-peroxidase [EC:1.11.1.21] | TCSA-Cases | ko01000 Enzymes | TCSA-Cases | None | None |
| K06169 | miaE | tRNA 2-(methylsulfanyl)-N6-isopentenyladenosine37 hydroxylase [EC:1.14.99.69] | TCSA-Cases | ko01000 Enzymes | TCSA-Cases | None | None |
| K06195 | apaG | ApaG protein | TCSA-Cases | None | TCSA-Cases | None | None |
| K07225 | hmuS | putative hemin transport protein | TCSA-Cases | None | TCSA-Cases | None | None |
| K08224 | ynfM | MFS transporter, YNFM family, putative membrane transport protein | TCSA-Cases | ko02000 Transporters | TCSA-Cases | None | None |
| K12541 | lapB | ATP-binding cassette, subfamily C, bacterial LapB | TCSA-Cases | ko02000 Transporters | TCSA-Cases | None | None |
| K14287 | ybdL | methionine transaminase [EC:2.6.1.88] | TCSA-Cases | ko01000 Enzymes | TCSA-Cases | None | None |
| K14393 | actP | cation/acetate symporter | TCSA-Cases | ko02000 Transporters | TCSA-Cases | None | None |
| K17247 | msrQ | methionine sulfoxide reductase heme-binding subunit | TCSA-Cases | None | TCSA-Cases | None | None |
| K19745 | acuI | acrylyl-CoA reductase (NADPH) [EC:1.3.1.-] | TCSA-Cases | ko01000 Enzymes | TCSA-Cases | None | None |
| K00228 | CPOX, hemF | coproporphyrinogen III oxidase [EC:1.3.3.3] | TCSA-Cases | ko01000 Enzymes | TCSA-Cases | None | None |
| K00242 | sdhD, frdD | succinate dehydrogenase membrane anchor subunit | TCSA-Cases | None | TCSA-Cases | None | None |
| K01630 | garL | 2-dehydro-3-deoxyglucarate aldolase [EC:4.1.2.20] | TCSA-Cases | ko01000 Enzymes | TCSA-Cases | None | None |
| K01669 | phr, PHR1 | deoxyribodipyrimidine photo-lyase [EC:4.1.99.3] | TCSA-Cases | ko01000 Enzymes | TCSA-Cases | None | None |
| K01753 | dsdA | D-serine dehydratase [EC:4.3.1.18] | TCSA-Cases | ko01000 Enzymes | TCSA-Cases | None | None |
| K02613 | paaE | ring-1,2-phenylacetyl-CoA epoxidase subunit PaaE | TCSA-Cases | None | TCSA-Cases | None | None |
| K02844 | waaG, rfaG | UDP-glucose:(heptosyl)LPS alpha-1,3-glucosyltransferase [EC:2.4.1.-] | TCSA-Cases | ko01000 Enzymes | TCSA-Cases | None | None |
| K03579 | hrpB | ATP-dependent helicase HrpB [EC:3.6.4.13] | TCSA-Cases | ko01000 Enzymes | TCSA-Cases | None | None |
| K04058 | yscW, sctW | type III secretion protein W | TCSA-Cases | ko02044 Secretion | TCSA-Cases | None | None |
| K04103 | ipdC | indolepyruvate decarboxylase [EC:4.1.1.74] | TCSA-Cases | ko01000 Enzymes | TCSA-Cases | None | None |
| K05846 | opuBD | osmoprotectant transport system permease protein | TCSA-Cases | ko02000 Transporters | TCSA-Cases | None | None |
| K06938 | K06938 | uncharacterized protein | TCSA-Cases | None | TCSA-Cases | None | None |
| K07165 | fecR | transmembrane sensor | TCSA-Cases | None | TCSA-Cases | None | None |
| K07246 | ttuC, dmlA | tartrate dehydrogenase/decarboxylase / D-malate dehydrogenase [EC:1.1.1.93 4.1.1.73 1.1.1.83] | TCSA-Cases | ko01000 Enzymes | TCSA-Cases | None | None |
| K07803 | zraP | zinc resistance-associated protein | TCSA-Cases | ko03110 Chaperones | TCSA-Cases | None | None |
| K12542 | lapC | membrane fusion protein, adhesin transport system | TCSA-Cases | ko02000 Transporters | TCSA-Cases | None | None |
| K12972 | ghrA | glyoxylate/hydroxypyruvate reductase [EC:1.1.1.79 1.1.1.81] | TCSA-Cases | ko01000 Enzymes | TCSA-Cases | None | None |
| K15034 | yaeJ | ribosome-associated protein | TCSA-Cases | ko03012 Translation | TCSA-Cases | None | None |
| K01457 | atzF | allophanate hydrolase [EC:3.5.1.54] | TCSA-Cases | ko01000 Enzymes | TCSA-Cases | None | None |
| K07506 | K07506 | AraC family transcriptional regulator | TCSA-Cases | ko03000 Transcription | TCSA-Cases | None | None |
| K00362 | nirB | nitrite reductase (NADH) large subunit [EC:1.7.1.15] | TCSA-Cases | ko01000 Enzymes | TCSA-Cases | None | None |
| K00799 | GST, gst | glutathione S-transferase [EC:2.5.1.18] | TCSA-Cases | ko01000 Enzymes | TCSA-Cases | None | None |
| K02167 | betI | TetR/AcrR family transcriptional regulator, transcriptional repressor of bet genes | TCSA-Cases | ko03000 Transcription | TCSA-Cases | None | None |
| K02774 | gatB, sgcB | galactitol PTS system EIIB component [EC:2.7.1.200] | TCSA-Cases | ko01000 Enzymes | TCSA-Cases | None | None |
| K03119 | tauD | taurine dioxygenase [EC:1.14.11.17] | TCSA-Cases | ko01000 Enzymes | TCSA-Cases | None | None |
| K03567 | gcvR | glycine cleavage system transcriptional repressor | TCSA-Cases | ko03000 Transcription | TCSA-Cases | None | None |
| K03926 | cutA | periplasmic divalent cation tolerance protein | TCSA-Cases | None | TCSA-Cases | None | None |
| K04091 | ssuD, msuD | alkanesulfonate monooxygenase [EC:1.14.14.5 1.14.14.34] | TCSA-Cases | ko01000 Enzymes | TCSA-Cases | None | None |
| K05836 | hutC | GntR family transcriptional regulator, histidine utilization repressor | TCSA-Cases | ko03000 Transcription | TCSA-Cases | None | None |
| K06916 | zapE | cell division protein ZapE | TCSA-Cases | ko03036 Chromosome | TCSA-Cases | None | None |
| K07393 | ECM4, yqjG | glutathionyl-hydroquinone reductase [EC:1.8.5.7] | TCSA-Cases | ko01000 Enzymes | TCSA-Cases | None | None |
| K07397 | yhfA | putative redox protein | TCSA-Cases | None | TCSA-Cases | None | None |
| K11890 | impM | type VI secretion system protein ImpM | TCSA-Cases | ko02044 Secretion | TCSA-Cases | None | None |
| K11897 | impF | type VI secretion system protein ImpF | TCSA-Cases | ko02044 Secretion | TCSA-Cases | None | None |
| K11922 | mngR, farR | GntR family transcriptional regulator, mannosyl-D-glycerate transport/metabolism system repressor | TCSA-Cases | ko03000 Transcription | None | None | None |
| K13635 | cbl | LysR family transcriptional regulator, cys regulon transcriptional activator | TCSA-Cases | ko03000 Transcription | TCSA-Cases | None | None |
| K16136 | malI | LacI family transcriptional regulator, maltose regulon regulatory protein | TCSA-Cases | ko03000 Transcription | TCSA-Cases | None | None |
| K19577 | ydhP | MFS transporter, DHA1 family, inner membrane transport protein | TCSA-Cases | ko02000 Transporters | TCSA-Cases | None | None |
| K02525 | kdgR | LacI family transcriptional regulator, kdg operon repressor | TCSA-Cases | ko03000 Transcription | TCSA-Cases | None | None |
| K06151 | E1.1.99.3A | gluconate 2-dehydrogenase alpha chain [EC:1.1.99.3] | TCSA-Cases | ko01000 Enzymes | TCSA-Cases | None | None |
| K06152 | E1.1.99.3G | gluconate 2-dehydrogenase gamma chain [EC:1.1.99.3] | TCSA-Cases | ko01000 Enzymes | TCSA-Cases | None | None |
| K00855 | PRK, prkB | phosphoribulokinase [EC:2.7.1.19] | TCSA-Cases | ko01000 Enzymes | TCSA-Cases | None | None |
| K01250 | rihA | pyrimidine-specific ribonucleoside hydrolase [EC:3.2.2.-] | TCSA-Cases | ko01000 Enzymes | TCSA-Cases | None | None |
| K01479 | hutG | formiminoglutamase [EC:3.5.3.8] | TCSA-Cases | ko01000 Enzymes | TCSA-Cases | None | None |
| K01692 | paaF, echA | enoyl-CoA hydratase [EC:4.2.1.17] | TCSA-Cases | ko01000 Enzymes | TCSA-Cases | None | None |
| K01908 | ACSS3, prpE | propionyl-CoA synthetase [EC:6.2.1.17] | TCSA-Cases | ko01000 Enzymes | TCSA-Cases | None | None |
| K02452 | gspC | general secretion pathway protein C | TCSA-Cases | ko02044 Secretion | TCSA-Cases | None | None |
| K02462 | gspM | general secretion pathway protein M | TCSA-Cases | ko02044 Secretion | TCSA-Cases | None | None |
| K02526 | kdgT | 2-keto-3-deoxygluconate permease | TCSA-Cases | ko02000 Transporters | TCSA-Cases | None | None |
| K03796 | bax | Bax protein | TCSA-Cases | None | TCSA-Cases | None | None |
| K03817 | rimL | ribosomal-protein-serine acetyltransferase [EC:2.3.1.-] | TCSA-Cases | ko01000 Enzymes | TCSA-Cases | None | None |
| K05874 | tsr | methyl-accepting chemotaxis protein I, serine sensor receptor | TCSA-Cases | ko02035 Bacterial | TCSA-Cases | None | None |
| K05921 | hpaG | 5-oxopent-3-ene-1,2,5-tricarboxylate decarboxylase / 2-hydroxyhepta-2,4-diene-1,7-dioate isomerase [EC:4.1.1.68 5.3.3.-] | TCSA-Cases | ko01000 Enzymes | TCSA-Cases | None | None |
| K06044 | treY, glgY | (1->4)-alpha-D-glucan 1-alpha-D-glucosylmutase [EC:5.4.99.15] | TCSA-Cases | ko01000 Enzymes | TCSA-Cases | None | None |
| K06165 | phnH | alpha-D-ribose 1-methylphosphonate 5-triphosphate synthase subunit PhnH [EC:2.7.8.37] | TCSA-Cases | ko01000 Enzymes | TCSA-Cases | None | None |
| K07274 | mipA, ompV | MipA family protein | TCSA-Cases | ko02000 Transporters | TCSA-Cases | None | None |
| K07290 | yhjG | AsmA family protein | TCSA-Cases | None | TCSA-Cases | None | None |
| K07396 | K07396 | putative protein-disulfide isomerase | TCSA-Cases | None | TCSA-Cases | None | None |
| K08344 | scsB | suppressor for copper-sensitivity B | TCSA-Cases | ko02000 Transporters | TCSA-Cases | None | None |
| K09470 | puuA | gamma-glutamylputrescine synthase [EC:6.3.1.11] | TCSA-Cases | ko01000 Enzymes | TCSA-Cases | None | None |
| K10037 | glnP | glutamine transport system permease protein | TCSA-Cases | ko02000 Transporters | TCSA-Cases | None | None |
| K15866 | paaG | 2-(1,2-epoxy-1,2-dihydrophenyl)acetyl-CoA isomerase [EC:5.3.3.18] | TCSA-Cases | ko01000 Enzymes | TCSA-Cases | None | None |
| K16348 | ecnB | entericidin B | TCSA-Cases | ko02000 Transporters | TCSA-Cases | None | None |
| K16841 | hpxA | allantoin racemase [EC:5.1.99.3] | TCSA-Cases | ko01000 Enzymes | TCSA-Cases | None | None |
| K18997 | cbpM | chaperone modulatory protein CbpM | TCSA-Cases | ko03036 Chromosome | TCSA-Cases | None | None |
| K19148 | tisB | small toxic protein TisB | TCSA-Cases | ko02048 Prokaryotic | TCSA-Cases | None | None |
| K19775 | exuR | GntR family transcriptional regulator, hexuronate regulon transcriptional repressor | TCSA-Cases | ko03000 Transcription | TCSA-Cases | None | None |
| K08964 | mtnB | methylthioribulose-1-phosphate dehydratase [EC:4.2.1.109] | TCSA-Cases | ko01000 Enzymes | TCSA-Cases | None | None |
| K09880 | mtnC, ENOPH1 | enolase-phosphatase E1 [EC:3.1.3.77] | TCSA-Cases | ko01000 Enzymes | TCSA-Cases | None | None |
| K05358 | quiA | quinate dehydrogenase (quinone) [EC:1.1.5.8] | TCSA-Cases | ko01000 Enzymes | TCSA-Cases | None | None |
| K09958 | K09958 | uncharacterized protein | TCSA-Cases | None | TCSA-Cases | None | None |
| K00116 | mqo | malate dehydrogenase (quinone) [EC:1.1.5.4] | TCSA-Cases | ko01000 Enzymes | TCSA-Cases | None | None |
| K00130 | betB, gbsA | betaine-aldehyde dehydrogenase [EC:1.2.1.8] | TCSA-Cases | ko01000 Enzymes | TCSA-Cases | None | None |
| K00138 | aldB | aldehyde dehydrogenase [EC:1.2.1.-] | TCSA-Cases | ko01000 Enzymes | TCSA-Cases | None | None |
| K00299 | ssuE, msuE | FMN reductase [EC:1.5.1.38] | TCSA-Cases | ko01000 Enzymes | TCSA-Cases | None | None |
| K00823 | puuE | 4-aminobutyrate aminotransferase [EC:2.6.1.19] | TCSA-Cases | ko01000 Enzymes | TCSA-Cases | None | None |
| K00906 | aceK | isocitrate dehydrogenase kinase/phosphatase [EC:2.7.11.5 3.1.3.-] | TCSA-Cases | ko01000 Enzymes | TCSA-Cases | None | None |
| K01058 | pldA | phospholipase A1/A2 [EC:3.1.1.32 3.1.1.4] | TCSA-Cases | ko01000 Enzymes | TCSA-Cases | None | None |
| K01070 | frmB, ESD, fghA | S-formylglutathione hydrolase [EC:3.1.2.12] | TCSA-Cases | ko01000 Enzymes | TCSA-Cases | None | None |
| K01093 | appA | 4-phytase / acid phosphatase [EC:3.1.3.26 3.1.3.2] | TCSA-Cases | ko01000 Enzymes | TCSA-Cases | None | None |
| K02024 | lamB | maltoporin | TCSA-Cases | ko02000 Transporters | TCSA-Cases | None | None |
| K02043 | phnF | GntR family transcriptional regulator, phosphonate transport system regulatory protein | TCSA-Cases | ko03000 Transcription | TCSA-Cases | None | None |
| K02345 | holE | DNA polymerase III subunit theta [EC:2.7.7.7] | TCSA-Cases | ko01000 Enzymes | TCSA-Cases | None | None |
| K02386 | flgA | flagellar basal body P-ring formation protein FlgA | TCSA-Cases | ko02035 Bacterial | TCSA-Cases | None | None |
| K02394 | flgI | flagellar P-ring protein FlgI | TCSA-Cases | ko02035 Bacterial | TCSA-Cases | None | None |
| K02457 | gspH | general secretion pathway protein H | TCSA-Cases | ko02044 Secretion | TCSA-Cases | None | None |
| K02458 | gspI | general secretion pathway protein I | TCSA-Cases | ko02044 Secretion | TCSA-Cases | None | None |
| K02460 | gspK | general secretion pathway protein K | TCSA-Cases | ko02044 Secretion | TCSA-Cases | None | None |
| K02565 | nagC | N-acetylglucosamine repressor | TCSA-Cases | ko03000 Transcription | TCSA-Cases | None | None |
| K03774 | slpA | FKBP-type peptidyl-prolyl cis-trans isomerase SlpA [EC:5.2.1.8] | TCSA-Cases | ko01000 Enzymes | TCSA-Cases | None | None |
| K04080 | ibpA | molecular chaperone IbpA | TCSA-Cases | ko03110 Chaperones | TCSA-Cases | None | None |
| K05780 | phnL | alpha-D-ribose 1-methylphosphonate 5-triphosphate synthase subunit PhnL [EC:2.7.8.37] | TCSA-Cases | ko01000 Enzymes | TCSA-Cases | None | None |
| K05781 | phnK | putative phosphonate transport system ATP-binding protein | TCSA-Cases | None | TCSA-Cases | None | None |
| K06163 | phnJ | alpha-D-ribose 1-methylphosphonate 5-phosphate C-P lyase [EC:4.7.1.1] | TCSA-Cases | ko01000 Enzymes | TCSA-Cases | None | None |
| K06164 | phnI | alpha-D-ribose 1-methylphosphonate 5-triphosphate synthase subunit PhnI [EC:2.7.8.37] | TCSA-Cases | ko01000 Enzymes | TCSA-Cases | None | None |
| K06166 | phnG | alpha-D-ribose 1-methylphosphonate 5-triphosphate synthase subunit PhnG [EC:2.7.8.37] | TCSA-Cases | ko01000 Enzymes | TCSA-Cases | None | None |
| K07349 | fimG | minor fimbrial subunit | TCSA-Cases | ko02044 Secretion | TCSA-Cases | None | None |
| K07589 | folX | D-erythro-7,8-dihydroneopterin triphosphate epimerase [EC:5.1.99.7] | TCSA-Cases | ko01000 Enzymes | TCSA-Cases | None | None |
| K07788 | mdtB | multidrug efflux pump | TCSA-Cases | ko02000 Transporters | TCSA-Cases | None | None |
| K08173 | ydfJ | MFS transporter, MHS family, metabolite:H+ symporter | TCSA-Cases | ko02000 Transporters | TCSA-Cases | None | None |
| K08178 | JEN | MFS transporter, SHS family, lactate transporter | TCSA-Cases | ko02000 Transporters | TCSA-Cases | None | None |
| K08195 | pcaK | MFS transporter, AAHS family, 4-hydroxybenzoate transporter | TCSA-Cases | ko02000 Transporters | TCSA-Cases | None | None |
| K08368 | yaaU | MFS transporter, putative metabolite transport protein | TCSA-Cases | ko02000 Transporters | TCSA-Cases | None | None |
| K08484 | ptsP | phosphotransferase system, enzyme I, PtsP [EC:2.7.3.9] | TCSA-Cases | ko01000 Enzymes | TCSA-Cases | None | None |
| K09017 | rutR | TetR/AcrR family transcriptional regulator | TCSA-Cases | ko03000 Transcription | TCSA-Cases | None | None |
| K09024 | rutF | flavin reductase [EC:1.5.1.-] | TCSA-Cases | ko01000 Enzymes | TCSA-Cases | None | None |
| K09472 | puuC, aldH | 4-(gamma-glutamylamino)butanal dehydrogenase [EC:1.2.1.99] | TCSA-Cases | ko01000 Enzymes | TCSA-Cases | None | None |
| K09473 | puuD | gamma-glutamyl-gamma-aminobutyrate hydrolase [EC:3.5.1.94] | TCSA-Cases | ko01000 Enzymes | TCSA-Cases | None | None |
| K09954 | K09954 | uncharacterized protein | TCSA-Cases | None | TCSA-Cases | None | None |
| K09972 | aapP, bztD | general L-amino acid transport system ATP-binding protein [EC:7.4.2.1] | TCSA-Cases | ko01000 Enzymes | TCSA-Cases | None | None |
| K09975 | K09975 | uncharacterized protein | TCSA-Cases | None | TCSA-Cases | None | None |
| K11073 | potF, spuD, spuE | putrescine transport system substrate-binding protein | TCSA-Cases | ko02000 Transporters | TCSA-Cases | None | None |
| K13497 | trpGD | anthranilate synthase/phosphoribosyltransferase [EC:4.1.3.27 2.4.2.18] | TCSA-Cases | ko01000 Enzymes | TCSA-Cases | None | None |
| K13896 | yejF | microcin C transport system ATP-binding protein | TCSA-Cases | ko02000 Transporters | TCSA-Cases | None | None |
| K13938 | folM | dihydromonapterin reductase / dihydrofolate reductase [EC:1.5.1.50 1.5.1.3] | TCSA-Cases | ko01000 Enzymes | TCSA-Cases | None | None |
| K14056 | puuR | HTH-type transcriptional regulator, repressor for puuD | TCSA-Cases | ko03000 Transcription | TCSA-Cases | None | None |
| K16074 | zntB | zinc transporter | TCSA-Cases | ko02000 Transporters | TCSA-Cases | None | None |
| K16347 | ecnA | entericidin A | TCSA-Cases | ko02000 Transporters | TCSA-Cases | None | None |
| K17463 | dgaF | 2-dehydro-3-deoxy-phosphogluconate aldolase [EC:4.1.2.14] | TCSA-Cases | ko01000 Enzymes | TCSA-Cases | None | None |
| K17468 | dgaE | D-glucosaminate-6-phosphate ammonia-lyase [EC:4.3.1.29] | TCSA-Cases | ko01000 Enzymes | TCSA-Cases | None | None |
| K19784 | chrR, NQR | chromate reductase, NAD(P)H dehydrogenase (quinone) | TCSA-Cases | None | TCSA-Cases | None | None |
| K00839 | pucG | (S)-ureidoglycine---glyoxylate transaminase [EC:2.6.1.112] | TCSA-Cases | ko01000 Enzymes | TCSA-Cases | None | None |
| K01941 | E6.3.4.6 | urea carboxylase [EC:6.3.4.6] | TCSA-Cases | ko01000 Enzymes | TCSA-Cases | None | None |
| K08967 | mtnD, mtnZ, ADI1 | 1,2-dihydroxy-3-keto-5-methylthiopentene dioxygenase [EC:1.13.11.53 1.13.11.54] | TCSA-Cases | ko01000 Enzymes | TCSA-Cases | None | None |
| K16842 | hpxB | allantoinase [EC:3.5.2.5] | TCSA-Cases | ko01000 Enzymes | TCSA-Cases | None | None |
| K19338 | nac | LysR family transcriptional regulator, nitrogen assimilation regulatory protein | TCSA-Cases | ko03000 Transcription | TCSA-Cases | None | None |
| K05375 | mbtH, nocI | MbtH protein | TCSA-Cases | ko01008 Polyketide | TCSA-Cases | None | None |
| K13408 | cvaA, mchE, raxA | membrane fusion protein | TCSA-Cases | ko02000 Transporters | TCSA-Cases | None | None |
| K13409 | cvaB, mchF, raxB | ATP-binding cassette, subfamily B, bacterial CvaB/MchF/RaxB | TCSA-Cases | ko02000 Transporters | TCSA-Cases | None | None |
| K17850 | ampR | LysR family transcriptional regulator, regulator of gene expression of beta-lactamase | TCSA-Cases | ko03000 Transcription | TCSA-Cases | None | None |
| K05793 | terB | tellurite resistance protein TerB | TCSA-Cases | None | None | None | None |
| K00090 | ghrB | glyoxylate/hydroxypyruvate/2-ketogluconate reductase [EC:1.1.1.79 1.1.1.81 1.1.1.215] | TCSA-Cases | ko01000 Enzymes | TCSA-Cases | None | None |
| K00121 | frmA, ADH5, adhC | S-(hydroxymethyl)glutathione dehydrogenase / alcohol dehydrogenase [EC:1.1.1.284 1.1.1.1] | TCSA-Cases | ko01000 Enzymes | TCSA-Cases | None | None |
| K00151 | hpaE, hpcC | 5-carboxymethyl-2-hydroxymuconic-semialdehyde dehydrogenase [EC:1.2.1.60] | TCSA-Cases | ko01000 Enzymes | TCSA-Cases | None | None |
| K00455 | hpaD, hpcB | 3,4-dihydroxyphenylacetate 2,3-dioxygenase [EC:1.13.11.15] | TCSA-Cases | ko01000 Enzymes | TCSA-Cases | None | None |
| K00573 | E2.1.1.77, pcm | protein-L-isoaspartate(D-aspartate) O-methyltransferase [EC:2.1.1.77] | TCSA-Cases | ko01000 Enzymes | TCSA-Cases | None | None |
| K00598 | tam | trans-aconitate 2-methyltransferase [EC:2.1.1.144] | TCSA-Cases | ko01000 Enzymes | TCSA-Cases | None | None |
| K00697 | otsA | trehalose 6-phosphate synthase [EC:2.4.1.15 2.4.1.347] | TCSA-Cases | ko01000 Enzymes | TCSA-Cases | None | None |
| K00835 | avtA | valine--pyruvate aminotransferase [EC:2.6.1.66] | TCSA-Cases | ko01000 Enzymes | TCSA-Cases | None | None |
| K01066 | aes | acetyl esterase [EC:3.1.1.-] | TCSA-Cases | ko01000 Enzymes | TCSA-Cases | None | None |
| K01478 | arcA | arginine deiminase [EC:3.5.3.6] | TCSA-Cases | ko01000 Enzymes | TCSA-Cases | None | None |
| K01631 | dgoA | 2-dehydro-3-deoxyphosphogalactonate aldolase [EC:4.1.2.21] | TCSA-Cases | ko01000 Enzymes | TCSA-Cases | None | None |
| K01758 | CTH | cystathionine gamma-lyase [EC:4.4.1.1] | TCSA-Cases | ko01000 Enzymes | TCSA-Cases | None | None |
| K01826 | hpaF, hpcD | 5-carboxymethyl-2-hydroxymuconate isomerase [EC:5.3.3.10] | TCSA-Cases | ko01000 Enzymes | TCSA-Cases | None | None |
| K02298 | cyoB | cytochrome o ubiquinol oxidase subunit I [EC:7.1.1.3] | TCSA-Cases | ko01000 Enzymes | TCSA-Cases | None | None |
| K02299 | cyoC | cytochrome o ubiquinol oxidase subunit III | TCSA-Cases | None | TCSA-Cases | None | None |
| K02393 | flgH | flagellar L-ring protein FlgH | TCSA-Cases | ko02035 Bacterial | TCSA-Cases | None | None |
| K02399 | flgN | flagellar biosynthesis protein FlgN | TCSA-Cases | ko02035 Bacterial | TCSA-Cases | None | None |
| K02402 | flhC | flagellar transcriptional activator FlhC | TCSA-Cases | ko02035 Bacterial | TCSA-Cases | None | None |
| K02403 | flhD | flagellar transcriptional activator FlhD | TCSA-Cases | ko02035 Bacterial | TCSA-Cases | None | None |
| K02423 | fliT | flagellar protein FliT | TCSA-Cases | ko02035 Bacterial | TCSA-Cases | None | None |
| K02508 | hpaA | AraC family transcriptional regulator, 4-hydroxyphenylacetate 3-monooxygenase operon regulatory protein | TCSA-Cases | ko03000 Transcription | TCSA-Cases | None | None |
| K02511 | hpaX | MFS transporter, ACS family, 4-hydroxyphenylacetate permease | TCSA-Cases | ko02000 Transporters | TCSA-Cases | None | None |
| K02617 | paaY | phenylacetic acid degradation protein | TCSA-Cases | None | TCSA-Cases | None | None |
| K02805 | wecE, rffA | dTDP-4-amino-4,6-dideoxygalactose transaminase [EC:2.6.1.59] | TCSA-Cases | ko01000 Enzymes | TCSA-Cases | None | None |
| K03087 | rpoS | RNA polymerase nonessential primary-like sigma factor | TCSA-Cases | ko03021 Transcription | TCSA-Cases | None | None |
| K03219 | yscC, sctC, ssaC | type III secretion protein C | TCSA-Cases | ko02044 Secretion | TCSA-Cases | None | None |
| K03224 | yscN, sctN, hrcN, ssaN | ATP synthase in type III secretion protein N [EC:7.4.2.8] | TCSA-Cases | ko01000 Enzymes | TCSA-Cases | None | None |
| K03291 | MFS.SET | MFS transporter, SET family, sugar efflux transporter | TCSA-Cases | ko02000 Transporters | TCSA-Cases | None | None |
| K03577 | acrR, smeT | TetR/AcrR family transcriptional regulator, acrAB operon repressor | TCSA-Cases | ko03000 Transcription | TCSA-Cases | None | None |
| K03672 | trxC | thioredoxin 2 [EC:1.8.1.8] | TCSA-Cases | ko01000 Enzymes | TCSA-Cases | None | None |
| K03717 | nhaR | LysR family transcriptional regulator, transcriptional activator of nhaA | TCSA-Cases | ko03000 Transcription | TCSA-Cases | None | None |
| K03810 | mviM | virulence factor | TCSA-Cases | None | TCSA-Cases | None | None |
| K03812 | rmf | ribosome modulation factor | TCSA-Cases | ko03009 Ribosome | TCSA-Cases | None | None |
| K03824 | yhbS | putative acetyltransferase [EC:2.3.1.-] | TCSA-Cases | ko01000 Enzymes | TCSA-Cases | None | None |
| K03893 | arsB | arsenical pump membrane protein | TCSA-Cases | ko02000 Transporters | TCSA-Cases | None | None |
| K04046 | yegD | hypothetical chaperone protein | TCSA-Cases | ko03110 Chaperones | TCSA-Cases | None | None |
| K05596 | iciA | LysR family transcriptional regulator, chromosome initiation inhibitor | TCSA-Cases | ko03000 Transcription | TCSA-Cases | None | None |
| K05712 | mhpA | 3-(3-hydroxy-phenyl)propionate hydroxylase [EC:1.14.13.127] | TCSA-Cases | ko01000 Enzymes | TCSA-Cases | None | None |
| K05875 | tar | methyl-accepting chemotaxis protein II, aspartate sensor receptor | TCSA-Cases | ko02035 Bacterial | TCSA-Cases | None | None |
| K05982 | E3.1.21.7, nfi | deoxyribonuclease V [EC:3.1.21.7] | TCSA-Cases | ko01000 Enzymes | TCSA-Cases | None | None |
| K06192 | pqiB | paraquat-inducible protein B | TCSA-Cases | None | TCSA-Cases | None | None |
| K06917 | selU, mnmH | tRNA 2-selenouridine synthase [EC:2.9.1.3] | TCSA-Cases | ko01000 Enzymes | TCSA-Cases | None | None |
| K07120 | K07120 | uncharacterized protein | TCSA-Cases | None | TCSA-Cases | None | None |
| K07127 | uraH, pucM, hiuH | 5-hydroxyisourate hydrolase [EC:3.5.2.17] | TCSA-Cases | ko01000 Enzymes | TCSA-Cases | None | None |
| K07289 | asmA | AsmA protein | TCSA-Cases | None | TCSA-Cases | None | None |
| K07661 | rstA | two-component system, OmpR family, response regulator RstA | TCSA-Cases | ko02022 Two-component | TCSA-Cases | None | None |
| K07664 | baeR, smeR | two-component system, OmpR family, response regulator BaeR | TCSA-Cases | ko02022 Two-component | TCSA-Cases | None | None |
| K07679 | evgS, bvgS | two-component system, NarL family, sensor histidine kinase EvgS [EC:2.7.13.3] | TCSA-Cases | ko01000 Enzymes | TCSA-Cases | None | None |
| K07709 | zraS, hydH | two-component system, NtrC family, sensor histidine kinase HydH [EC:2.7.13.3] | TCSA-Cases | ko01000 Enzymes | TCSA-Cases | None | None |
| K07746 | parD1_3_4 | antitoxin ParD1/3/4 | TCSA-Cases | ko02048 Prokaryotic | TCSA-Cases | None | None |
| K07789 | mdtC | multidrug efflux pump | TCSA-Cases | ko02000 Transporters | TCSA-Cases | None | None |
| K09456 | aidB | putative acyl-CoA dehydrogenase | TCSA-Cases | None | TCSA-Cases | None | None |
| K09823 | zur | Fur family transcriptional regulator, zinc uptake regulator | TCSA-Cases | ko03000 Transcription | TCSA-Cases | None | None |
| K09857 | K09857 | uncharacterized protein | TCSA-Cases | None | TCSA-Cases | None | None |
| K09915 | K09915 | uncharacterized protein | TCSA-Cases | None | TCSA-Cases | None | None |
| K09933 | mtfA | MtfA peptidase | TCSA-Cases | ko01002 Peptidases | TCSA-Cases | None | None |
| K09978 | K09978 | uncharacterized protein | TCSA-Cases | None | TCSA-Cases | None | None |
| K10036 | glnH | glutamine transport system substrate-binding protein | TCSA-Cases | ko02000 Transporters | TCSA-Cases | None | None |
| K11074 | potI, spuH | putrescine transport system permease protein | TCSA-Cases | ko02000 Transporters | TCSA-Cases | None | None |
| K11075 | potH, spuG | putrescine transport system permease protein | TCSA-Cases | ko02000 Transporters | TCSA-Cases | None | None |
| K11076 | potG, spuF | putrescine transport system ATP-binding protein [EC:7.6.2.16] | TCSA-Cases | ko01000 Enzymes | TCSA-Cases | None | None |
| K11177 | yagR | xanthine dehydrogenase YagR molybdenum-binding subunit [EC:1.17.1.4] | TCSA-Cases | ko01000 Enzymes | TCSA-Cases | None | None |
| K11178 | yagS | xanthine dehydrogenase YagS FAD-binding subunit [EC:1.17.1.4] | TCSA-Cases | ko01000 Enzymes | TCSA-Cases | None | None |
| K11535 | nupC | nucleoside transport protein | TCSA-Cases | ko02000 Transporters | TCSA-Cases | None | None |
| K11925 | sgrR | SgrR family transcriptional regulator | TCSA-Cases | ko03000 Transcription | TCSA-Cases | None | None |
| K11932 | uspG | universal stress protein G | TCSA-Cases | None | TCSA-Cases | None | None |
| K12266 | norR | anaerobic nitric oxide reductase transcription regulator | TCSA-Cases | ko03000 Transcription | TCSA-Cases | None | None |
| K12339 | cysM | S-sulfo-L-cysteine synthase (O-acetyl-L-serine-dependent) [EC:2.5.1.144] | TCSA-Cases | ko01000 Enzymes | TCSA-Cases | None | None |
| K13483 | yagT | xanthine dehydrogenase YagT iron-sulfur-binding subunit | TCSA-Cases | None | TCSA-Cases | None | None |
| K13795 | citB, tcuB | citrate/tricarballylate utilization protein | TCSA-Cases | None | TCSA-Cases | None | None |
| K13893 | yejA | microcin C transport system substrate-binding protein | TCSA-Cases | ko02000 Transporters | TCSA-Cases | None | None |
| K13894 | yejB | microcin C transport system permease protein | TCSA-Cases | ko02000 Transporters | TCSA-Cases | None | None |
| K13895 | yejE | microcin C transport system permease protein | TCSA-Cases | ko02000 Transporters | TCSA-Cases | None | None |
| K14057 | abgR | LysR family transcriptional regulator, regulator of abg operon | TCSA-Cases | ko03000 Transcription | TCSA-Cases | None | None |
| K14063 | feaR | AraC family transcriptional regulator, positive regulator of tynA and feaB | TCSA-Cases | ko03000 Transcription | TCSA-Cases | None | None |
| K15551 | tauA | taurine transport system substrate-binding protein | TCSA-Cases | ko02000 Transporters | TCSA-Cases | None | None |
| K16692 | etk-wzc | tyrosine-protein kinase Etk/Wzc [EC:2.7.10.3] | TCSA-Cases | ko01000 Enzymes | TCSA-Cases | None | None |
| K19334 | tabA | biofilm protein TabA | TCSA-Cases | ko02048 Prokaryotic | TCSA-Cases | None | None |
| K19337 | hexR | RpiR family transcriptional regulator, carbohydrate utilization regulator | TCSA-Cases | ko03000 Transcription | TCSA-Cases | None | None |
| K03973 | pspC | phage shock protein C | TCSA-Cases | ko03000 Transcription | TCSA-Cases | None | None |
| K10538 | araH | L-arabinose transport system permease protein | TCSA-Cases | ko02000 Transporters | TCSA-Cases | None | None |
| K00117 | gcd | quinoprotein glucose dehydrogenase [EC:1.1.5.2] | TCSA-Cases | ko01000 Enzymes | TCSA-Cases | None | None |
| K00322 | sthA, udhA | NAD(P) transhydrogenase [EC:1.6.1.1] | TCSA-Cases | ko01000 Enzymes | TCSA-Cases | None | None |
| K00363 | nirD | nitrite reductase (NADH) small subunit [EC:1.7.1.15] | TCSA-Cases | ko01000 Enzymes | TCSA-Cases | None | None |
| K00529 | hcaD | 3-phenylpropionate/trans-cinnamate dioxygenase ferredoxin reductase component [EC:1.18.1.3] | TCSA-Cases | ko01000 Enzymes | TCSA-Cases | None | None |
| K00673 | astA | arginine N-succinyltransferase [EC:2.3.1.109] | TCSA-Cases | ko01000 Enzymes | TCSA-Cases | None | None |
| K00840 | astC | succinylornithine aminotransferase [EC:2.6.1.81] | TCSA-Cases | ko01000 Enzymes | TCSA-Cases | None | None |
| K00932 | tdcD | propionate kinase [EC:2.7.2.15] | TCSA-Cases | ko01000 Enzymes | TCSA-Cases | None | None |
| K01194 | TREH, treA, treF | alpha,alpha-trehalase [EC:3.2.1.28] | TCSA-Cases | ko01000 Enzymes | TCSA-Cases | None | None |
| K01521 | cdh | CDP-diacylglycerol pyrophosphatase [EC:3.6.1.26] | TCSA-Cases | ko01000 Enzymes | TCSA-Cases | None | None |
| K01637 | E4.1.3.1, aceA | isocitrate lyase [EC:4.1.3.1] | TCSA-Cases | ko01000 Enzymes | TCSA-Cases | None | None |
| K01825 | fadB | 3-hydroxyacyl-CoA dehydrogenase / enoyl-CoA hydratase / 3-hydroxybutyryl-CoA epimerase / enoyl-CoA isomerase [EC:1.1.1.35 4.2.1.17 5.1.2.3 5.3.3.8] | TCSA-Cases | ko01000 Enzymes | TCSA-Cases | None | None |
| K02297 | cyoA | cytochrome o ubiquinol oxidase subunit II [EC:7.1.1.3] | TCSA-Cases | ko01000 Enzymes | TCSA-Cases | None | None |
| K02300 | cyoD | cytochrome o ubiquinol oxidase subunit IV | TCSA-Cases | None | TCSA-Cases | None | None |
| K02336 | polB | DNA polymerase II [EC:2.7.7.7] | TCSA-Cases | ko01000 Enzymes | TCSA-Cases | None | None |
| K02459 | gspJ | general secretion pathway protein J | TCSA-Cases | ko02044 Secretion | TCSA-Cases | None | None |
| K02461 | gspL | general secretion pathway protein L | TCSA-Cases | ko02044 Secretion | TCSA-Cases | None | None |
| K02855 | rhaS | AraC family transcriptional regulator, L-rhamnose operon regulatory protein RhaS | TCSA-Cases | ko03000 Transcription | TCSA-Cases | None | None |
| K03112 | damX | DamX protein | TCSA-Cases | None | TCSA-Cases | None | None |
| K03208 | wcaI | putative colanic acid biosynthesis glycosyltransferase WcaI | TCSA-Cases | None | TCSA-Cases | None | None |
| K03280 | waaK, rfaK | UDP-N-acetylglucosamine:(glucosyl)LPS alpha-1,2-N-acetylglucosaminyltransferase [EC:2.4.1.56] | TCSA-Cases | ko01000 Enzymes | TCSA-Cases | None | None |
| K03658 | helD | DNA helicase IV [EC:5.6.2.4] | TCSA-Cases | ko01000 Enzymes | TCSA-Cases | None | None |
| K03669 | mdoH | membrane glycosyltransferase [EC:2.4.1.-] | TCSA-Cases | ko01000 Enzymes | TCSA-Cases | None | None |
| K03670 | mdoG | periplasmic glucans biosynthesis protein | TCSA-Cases | None | TCSA-Cases | None | None |
| K03747 | smg | Smg protein | TCSA-Cases | None | TCSA-Cases | None | None |
| K03761 | kgtP | MFS transporter, MHS family, alpha-ketoglutarate permease | TCSA-Cases | ko02000 Transporters | TCSA-Cases | None | None |
| K03791 | K03791 | putative chitinase | TCSA-Cases | None | TCSA-Cases | None | None |
| K03809 | wrbA | NAD(P)H dehydrogenase (quinone) [EC:1.6.5.2] | TCSA-Cases | ko01000 Enzymes | TCSA-Cases | None | None |
| K03840 | fldB | flavodoxin II | TCSA-Cases | None | TCSA-Cases | None | None |
| K03919 | alkB | DNA oxidative demethylase [EC:1.14.11.33] | TCSA-Cases | ko01000 Enzymes | TCSA-Cases | None | None |
| K03970 | pspB | phage shock protein B | TCSA-Cases | ko02048 Prokaryotic | TCSA-Cases | None | None |
| K03974 | pspF | psp operon transcriptional activator | TCSA-Cases | ko03000 Transcription | TCSA-Cases | None | None |
| K04065 | osmY | hyperosmotically inducible periplasmic protein | TCSA-Cases | None | TCSA-Cases | None | None |
| K04775 | ydgD | protease YdgD [EC:3.4.21.-] | TCSA-Cases | ko01000 Enzymes | TCSA-Cases | None | None |
| K05396 | dcyD | D-cysteine desulfhydrase [EC:4.4.1.15] | TCSA-Cases | ko01000 Enzymes | TCSA-Cases | None | None |
| K05517 | tsx | nucleoside-specific channel-forming protein | TCSA-Cases | ko02000 Transporters | TCSA-Cases | None | None |
| K05526 | astE | succinylglutamate desuccinylase [EC:3.5.1.96] | TCSA-Cases | ko01000 Enzymes | TCSA-Cases | None | None |
| K05591 | dbpA | ATP-dependent RNA helicase DbpA [EC:3.6.4.13] | TCSA-Cases | ko01000 Enzymes | TCSA-Cases | None | None |
| K05839 | hha | haemolysin expression modulating protein | TCSA-Cases | ko02048 Prokaryotic | TCSA-Cases | None | None |
| K05877 | tap | methyl-accepting chemotaxis protein IV, peptide sensor receptor | TCSA-Cases | ko02035 Bacterial | TCSA-Cases | None | None |
| K05997 | sufA | Fe-S cluster assembly protein SufA | TCSA-Cases | None | TCSA-Cases | None | None |
| K06078 | lpp | murein lipoprotein | TCSA-Cases | ko01011 Peptidoglycan | TCSA-Cases | None | None |
| K06445 | fadE | acyl-CoA dehydrogenase [EC:1.3.99.-] | TCSA-Cases | ko01000 Enzymes | TCSA-Cases | None | None |
| K06447 | astD | succinylglutamic semialdehyde dehydrogenase [EC:1.2.1.71] | TCSA-Cases | ko01000 Enzymes | TCSA-Cases | None | None |
| K07070 | K07070 | uncharacterized protein | TCSA-Cases | None | TCSA-Cases | None | None |
| K07312 | ynfH | Tat-targeted selenate reductase subunit YnfH | TCSA-Cases | ko02000 Transporters | TCSA-Cases | None | None |
| K07336 | K07336 | PKHD-type hydroxylase [EC:1.14.11.-] | TCSA-Cases | ko01000 Enzymes | TCSA-Cases | None | None |
| K07351 | fimI | fimbrial protein | TCSA-Cases | ko02035 Bacterial | TCSA-Cases | None | None |
| K07592 | tdcA | LysR family transcriptional regulator, tdc operon transcriptional activator | TCSA-Cases | ko03000 Transcription | TCSA-Cases | None | None |
| K07639 | rstB | two-component system, OmpR family, sensor histidine kinase RstB [EC:2.7.13.3] | TCSA-Cases | ko01000 Enzymes | TCSA-Cases | None | None |
| K07687 | rcsB | two-component system, NarL family, captular synthesis response regulator RcsB | TCSA-Cases | ko02022 Two-component | TCSA-Cases | None | None |
| K07700 | dpiB, citA | two-component system, CitB family, cit operon sensor histidine kinase CitA [EC:2.7.13.3] | TCSA-Cases | ko01000 Enzymes | TCSA-Cases | None | None |
| K07701 | dcuS | two-component system, CitB family, sensor histidine kinase DcuS [EC:2.7.13.3] | TCSA-Cases | ko01000 Enzymes | TCSA-Cases | None | None |
| K07702 | dpiA, citB | two-component system, CitB family, response regulator CitB | TCSA-Cases | ko02022 Two-component | TCSA-Cases | None | None |
| K07711 | glrK, qseE | two-component system, NtrC family, sensor histidine kinase GlrK [EC:2.7.13.3] | TCSA-Cases | ko01000 Enzymes | TCSA-Cases | None | None |
| K07715 | glrR, qseF | two-component system, NtrC family, response regulator GlrR | TCSA-Cases | ko02022 Two-component | TCSA-Cases | None | None |
| K07733 | alpA | prophage regulatory protein | TCSA-Cases | ko03000 Transcription | TCSA-Cases | None | None |
| K07740 | rsd | regulator of sigma D | TCSA-Cases | None | TCSA-Cases | None | None |
| K07771 | basR | two-component system, OmpR family, response regulator BasR | TCSA-Cases | ko02022 Two-component | TCSA-Cases | None | None |
| K07782 | sdiA | LuxR family transcriptional regulator, quorum-sensing system regulator SdiA | TCSA-Cases | ko03000 Transcription | TCSA-Cases | None | None |
| K07806 | arnB, pmrH | UDP-4-amino-4-deoxy-L-arabinose-oxoglutarate aminotransferase [EC:2.6.1.87] | TCSA-Cases | ko01000 Enzymes | TCSA-Cases | None | None |
| K08162 | mdtH | MFS transporter, DHA1 family, multidrug resistance protein | TCSA-Cases | ko02000 Transporters | TCSA-Cases | None | None |
| K08163 | mdtL | MFS transporter, DHA1 family, multidrug resistance protein | TCSA-Cases | ko02000 Transporters | TCSA-Cases | None | None |
| K08194 | dgoT | MFS transporter, ACS family, D-galactonate transporter | TCSA-Cases | ko02000 Transporters | TCSA-Cases | None | None |
| K08994 | yneE, BEST | ion channel-forming bestrophin family protein | TCSA-Cases | ko02000 Transporters | TCSA-Cases | None | None |
| K09475 | ompC | outer membrane pore protein C | TCSA-Cases | ko02000 Transporters | TCSA-Cases | None | None |
| K09612 | iap | alkaline phosphatase isozyme conversion protein [EC:3.4.11.-] | TCSA-Cases | ko01000 Enzymes | TCSA-Cases | None | None |
| K09788 | prpF | 2-methylaconitate isomerase [EC:5.3.3.-] | TCSA-Cases | ko01000 Enzymes | TCSA-Cases | None | None |
| K09916 | K09916 | uncharacterized protein | TCSA-Cases | None | TCSA-Cases | None | None |
| K09996 | artJ | arginine transport system substrate-binding protein | TCSA-Cases | ko02000 Transporters | TCSA-Cases | None | None |
| K10013 | argT | lysine/arginine/ornithine transport system substrate-binding protein | TCSA-Cases | ko02000 Transporters | TCSA-Cases | None | None |
| K10014 | hisJ | histidine transport system substrate-binding protein | TCSA-Cases | ko02000 Transporters | TCSA-Cases | None | None |
| K10038 | glnQ | glutamine transport system ATP-binding protein [EC:7.4.2.1] | TCSA-Cases | ko01000 Enzymes | TCSA-Cases | None | None |
| K10544 | xylH | D-xylose transport system permease protein | TCSA-Cases | ko02000 Transporters | TCSA-Cases | None | None |
| K11192 | murP | N-acetylmuramic acid PTS system EIICB component [EC:2.7.1.192] | TCSA-Cases | ko01000 Enzymes | TCSA-Cases | None | None |
| K11685 | stpA | DNA-binding protein StpA | TCSA-Cases | ko03036 Chromosome | TCSA-Cases | None | None |
| K11732 | pheP | phenylalanine-specific permease | TCSA-Cases | ko02000 Transporters | TCSA-Cases | None | None |
| K11911 | vasL | type VI secretion system protein VasL | TCSA-Cases | ko02044 Secretion | TCSA-Cases | None | None |
| K11926 | crl | sigma factor-binding protein Crl | TCSA-Cases | ko03000 Transcription | TCSA-Cases | None | None |
| K11940 | hspQ | heat shock protein HspQ | TCSA-Cases | ko03036 Chromosome | TCSA-Cases | None | None |
| K11941 | mdoC | glucans biosynthesis protein C [EC:2.1.-.-] | TCSA-Cases | ko01000 Enzymes | TCSA-Cases | None | None |
| K12149 | dinI | DNA-damage-inducible protein I | TCSA-Cases | ko03400 DNA | TCSA-Cases | None | None |
| K12962 | arnE | undecaprenyl phosphate-alpha-L-ara4N flippase subunit ArnE | TCSA-Cases | ko02000 Transporters | TCSA-Cases | None | None |
| K12973 | pagP, crcA | lipid IVA palmitoyltransferase [EC:2.3.1.251] | TCSA-Cases | ko01000 Enzymes | TCSA-Cases | None | None |
| K13014 | arnD | undecaprenyl phosphate-alpha-L-ara4FN deformylase [EC:3.5.1.-] | TCSA-Cases | ko01000 Enzymes | TCSA-Cases | None | None |
| K13053 | sulA | cell division inhibitor SulA | TCSA-Cases | ko03036 Chromosome | TCSA-Cases | None | None |
| K13629 | dsdX | D-serine transporter | TCSA-Cases | ko02000 Transporters | TCSA-Cases | None | None |
| K13636 | dsdC | LysR family transcriptional regulator, D-serine deaminase activator | TCSA-Cases | ko03000 Transcription | TCSA-Cases | None | None |
| K13639 | soxR | MerR family transcriptional regulator, redox-sensitive transcriptional activator SoxR | TCSA-Cases | ko03000 Transcription | TCSA-Cases | None | None |
| K13683 | wcaE | putative colanic acid biosynthesis glycosyltransferase WcaE [EC:2.4.-.-] | TCSA-Cases | ko01000 Enzymes | TCSA-Cases | None | None |
| K14051 | gmr, pdeR | c-di-GMP phosphodiesterase Gmr [EC:3.1.4.52] | TCSA-Cases | ko01000 Enzymes | TCSA-Cases | None | None |
| K15836 | fhlA | formate hydrogenlyase transcriptional activator | TCSA-Cases | ko03000 Transcription | TCSA-Cases | None | None |
| K16090 | fiu | catecholate siderophore receptor | TCSA-Cases | ko02000 Transporters | TCSA-Cases | None | None |
| K16291 | erfK | L,D-transpeptidase ErfK/SrfK | TCSA-Cases | ko01002 Peptidases | TCSA-Cases | None | None |
| K18889 | mdlA, smdA | ATP-binding cassette, subfamily B, multidrug efflux pump | TCSA-Cases | ko02000 Transporters | TCSA-Cases | None | None |
| K18890 | mdlB, smdB | ATP-binding cassette, subfamily B, multidrug efflux pump | TCSA-Cases | ko02000 Transporters | TCSA-Cases | None | None |
| K19688 | bssR | biofilm regulator BssR | TCSA-Cases | ko02048 Prokaryotic | TCSA-Cases | None | None |
| K05372 | ybtA | AraC family transcriptional regulator | TCSA-Cases | ko03000 Transcription | None | None | None |
| K05373 | ybtX, irp8 | MFS transporter, putative signal transducer | TCSA-Cases | ko02000 Transporters | None | None | None |
| K15721 | fyuA | pesticin/yersiniabactin receptor | TCSA-Cases | ko02000 Transporters | None | None | None |
| K00137 | prr | aminobutyraldehyde dehydrogenase [EC:1.2.1.19] | TCSA-Cases | ko01000 Enzymes | TCSA-Cases | None | None |
| K00216 | entA | 2,3-dihydro-2,3-dihydroxybenzoate dehydrogenase [EC:1.3.1.28] | TCSA-Cases | ko01000 Enzymes | TCSA-Cases | None | None |
| K00427 | lldP, lctP | L-lactate permease | TCSA-Cases | ko02000 Transporters | TCSA-Cases | None | None |
| K00892 | gsk | inosine kinase [EC:2.7.1.73] | TCSA-Cases | ko01000 Enzymes | TCSA-Cases | None | None |
| K01146 | xni | protein Xni | TCSA-Cases | None | TCSA-Cases | None | None |
| K01169 | rna | ribonuclease I (enterobacter ribonuclease) [EC:4.6.1.21] | TCSA-Cases | ko01000 Enzymes | TCSA-Cases | None | None |
| K01252 | entB, dhbB, vibB, mxcF | bifunctional isochorismate lyase / aryl carrier protein [EC:3.3.2.1 6.3.2.14] | TCSA-Cases | ko01000 Enzymes | TCSA-Cases | None | None |
| K01484 | astB | succinylarginine dihydrolase [EC:3.5.3.23] | TCSA-Cases | ko01000 Enzymes | TCSA-Cases | None | None |
| K01638 | aceB, glcB | malate synthase [EC:2.3.3.9] | TCSA-Cases | ko01000 Enzymes | TCSA-Cases | None | None |
| K01690 | edd | phosphogluconate dehydratase [EC:4.2.1.12] | TCSA-Cases | ko01000 Enzymes | TCSA-Cases | None | None |
| K01920 | gshB | glutathione synthase [EC:6.3.2.3] | TCSA-Cases | ko01000 Enzymes | TCSA-Cases | None | None |
| K02042 | phnE | phosphonate transport system permease protein | TCSA-Cases | ko02000 Transporters | TCSA-Cases | None | None |
| K02079 | agaA | N-acetylgalactosamine-6-phosphate deacetylase [EC:3.5.1.25] | TCSA-Cases | ko01000 Enzymes | TCSA-Cases | None | None |
| K02170 | bioH | pimeloyl-[acyl-carrier protein] methyl ester esterase [EC:3.1.1.85] | TCSA-Cases | ko01000 Enzymes | TCSA-Cases | None | None |
| K02255 | ftnB | ferritin-like protein 2 | TCSA-Cases | None | TCSA-Cases | None | None |
| K02317 | dnaT | DNA replication protein DnaT | TCSA-Cases | ko03400 DNA | TCSA-Cases | None | None |
| K02362 | entD | enterobactin synthetase component D [EC:6.3.2.14 2.7.8.-] | TCSA-Cases | ko01000 Enzymes | TCSA-Cases | None | None |
| K02364 | entF | L-serine---[L-seryl-carrier protein] ligase [EC:6.3.2.14 6.2.1.72] | TCSA-Cases | ko01000 Enzymes | TCSA-Cases | None | None |
| K02425 | fliZ | regulator of sigma S factor FliZ | TCSA-Cases | ko02035 Bacterial | TCSA-Cases | None | None |
| K02455 | gspF | general secretion pathway protein F | TCSA-Cases | ko02044 Secretion | TCSA-Cases | None | None |
| K02485 | rssB, hnr | two-component system, response regulator | TCSA-Cases | ko02022 Two-component | TCSA-Cases | None | None |
| K02609 | paaA | ring-1,2-phenylacetyl-CoA epoxidase subunit PaaA [EC:1.14.13.149] | TCSA-Cases | ko01000 Enzymes | TCSA-Cases | None | None |
| K02610 | paaB | ring-1,2-phenylacetyl-CoA epoxidase subunit PaaB | TCSA-Cases | None | TCSA-Cases | None | None |
| K02611 | paaC | ring-1,2-phenylacetyl-CoA epoxidase subunit PaaC [EC:1.14.13.149] | TCSA-Cases | ko01000 Enzymes | TCSA-Cases | None | None |
| K02612 | paaD | ring-1,2-phenylacetyl-CoA epoxidase subunit PaaD | TCSA-Cases | None | TCSA-Cases | None | None |
| K02616 | paaX | phenylacetic acid degradation operon negative regulatory protein | TCSA-Cases | ko03000 Transcription | TCSA-Cases | None | None |
| K02618 | paaZ | oxepin-CoA hydrolase / 3-oxo-5,6-dehydrosuberyl-CoA semialdehyde dehydrogenase [EC:3.3.2.12 1.2.1.91] | TCSA-Cases | ko01000 Enzymes | TCSA-Cases | None | None |
| K02846 | solA | N-methyl-L-tryptophan oxidase [EC:1.5.3.-] | TCSA-Cases | ko01000 Enzymes | TCSA-Cases | None | None |
| K02853 | wzyE, rffT | enterobacterial common antigen polymerase [EC:2.4.1.-] | TCSA-Cases | ko01000 Enzymes | TCSA-Cases | None | None |
| K02854 | rhaR | AraC family transcriptional regulator, L-rhamnose operon transcriptional activator RhaR | TCSA-Cases | ko03000 Transcription | TCSA-Cases | None | None |
| K02972 | sra | stationary-phase-induced ribosome-associated protein | TCSA-Cases | ko03009 Ribosome | TCSA-Cases | None | None |
| K03228 | yscT, sctT, hrcT, ssaT | type III secretion protein T | TCSA-Cases | ko02044 Secretion | TCSA-Cases | None | None |
| K03279 | waaJ, rfaJ | UDP-glucose:(galactosyl)LPS alpha-1,2-glucosyltransferase [EC:2.4.1.58] | TCSA-Cases | ko01000 Enzymes | TCSA-Cases | None | None |
| K03417 | prpB | methylisocitrate lyase [EC:4.1.3.30] | TCSA-Cases | ko01000 Enzymes | TCSA-Cases | None | None |
| K03425 | tatE | sec-independent protein translocase protein TatE | TCSA-Cases | ko02044 Secretion | TCSA-Cases | None | None |
| K03468 | aaeB | p-hydroxybenzoic acid efflux pump subunit AaeB | TCSA-Cases | ko02000 Transporters | TCSA-Cases | None | None |
| K03472 | epd | D-erythrose 4-phosphate dehydrogenase [EC:1.2.1.72] | TCSA-Cases | ko01000 Enzymes | TCSA-Cases | None | None |
| K03516 | flhE | flagellar protein FlhE | TCSA-Cases | ko02035 Bacterial | TCSA-Cases | None | None |
| K03618 | hyaF | hydrogenase-1 operon protein HyaF | TCSA-Cases | None | TCSA-Cases | None | None |
| K03649 | mug | double-stranded uracil-DNA glycosylase [EC:3.2.2.28] | TCSA-Cases | ko01000 Enzymes | TCSA-Cases | None | None |
| K03668 | hslJ | heat shock protein HslJ | TCSA-Cases | None | TCSA-Cases | None | None |
| K03712 | marR | MarR family transcriptional regulator, multiple antibiotic resistance protein MarR | TCSA-Cases | ko03000 Transcription | TCSA-Cases | None | None |
| K03762 | proP | MFS transporter, MHS family, proline/betaine transporter | TCSA-Cases | ko02000 Transporters | TCSA-Cases | None | None |
| K03805 | dsbG | thiol:disulfide interchange protein DsbG | TCSA-Cases | ko03110 Chaperones | TCSA-Cases | None | None |
| K03807 | ampE | AmpE protein | TCSA-Cases | None | TCSA-Cases | None | None |
| K03819 | wcaB | putative colanic acid biosynthesis acetyltransferase WcaB [EC:2.3.1.-] | TCSA-Cases | ko01000 Enzymes | TCSA-Cases | None | None |
| K03838 | tdcC | threonine transporter | TCSA-Cases | ko02000 Transporters | TCSA-Cases | None | None |
| K03971 | pspD | phage shock protein D | TCSA-Cases | None | TCSA-Cases | None | None |
| K04064 | osmE | osmotically inducible lipoprotein OsmE | TCSA-Cases | None | TCSA-Cases | None | None |
| K04333 | csgD | LuxR family transcriptional regulator, csgAB operon transcriptional regulatory protein | TCSA-Cases | ko03000 Transcription | TCSA-Cases | None | None |
| K04335 | csgB | minor curlin subunit | TCSA-Cases | ko02044 Secretion | TCSA-Cases | None | None |
| K04336 | csgC | curli production protein | TCSA-Cases | ko02044 Secretion | TCSA-Cases | None | None |
| K04337 | csgE | curli production assembly/transport component CsgE | TCSA-Cases | ko02044 Secretion | TCSA-Cases | None | None |
| K05368 | fre, ubiB | NAD(P)H-flavin reductase [EC:1.5.1.41] | TCSA-Cases | ko01000 Enzymes | TCSA-Cases | None | None |
| K05594 | elaB | ElaB protein | TCSA-Cases | None | TCSA-Cases | None | None |
| K05777 | ynjB | putative thiamine transport system substrate-binding protein | TCSA-Cases | ko02000 Transporters | TCSA-Cases | None | None |
| K05778 | ynjC | putative thiamine transport system permease protein | TCSA-Cases | ko02000 Transporters | TCSA-Cases | None | None |
| K05779 | ynjD | putative thiamine transport system ATP-binding protein | TCSA-Cases | ko02000 Transporters | TCSA-Cases | None | None |
| K05782 | benE | benzoate membrane transport protein | TCSA-Cases | ko02000 Transporters | TCSA-Cases | None | None |
| K05785 | rfaH | transcriptional antiterminator RfaH | TCSA-Cases | ko03000 Transcription | TCSA-Cases | None | None |
| K05811 | yfiM | putative lipoprotein | TCSA-Cases | None | TCSA-Cases | None | None |
| K05816 | ugpC | sn-glycerol 3-phosphate transport system ATP-binding protein [EC:7.6.2.10] | TCSA-Cases | ko01000 Enzymes | TCSA-Cases | None | None |
| K05984 | cho | excinuclease Cho [EC:3.1.25.-] | TCSA-Cases | ko01000 Enzymes | TCSA-Cases | None | None |
| K06006 | cpxP, spy | periplasmic protein CpxP/Spy | TCSA-Cases | ko03110 Chaperones | TCSA-Cases | None | None |
| K06073 | btuC | vitamin B12 transport system permease protein | TCSA-Cases | ko02000 Transporters | TCSA-Cases | None | None |
| K06074 | btuD | vitamin B12 transport system ATP-binding protein [EC:7.6.2.8] | TCSA-Cases | ko01000 Enzymes | TCSA-Cases | None | None |
| K06080 | rcsF | RcsF protein | TCSA-Cases | None | TCSA-Cases | None | None |
| K06140 | rnk | regulator of nucleoside diphosphate kinase | TCSA-Cases | ko03000 Transcription | TCSA-Cases | None | None |
| K06141 | tsgA | MFS transporter, TsgA protein | TCSA-Cases | ko02000 Transporters | TCSA-Cases | None | None |
| K06144 | uspB | universal stress protein B | TCSA-Cases | ko02000 Transporters | TCSA-Cases | None | None |
| K06157 | idnT | Gnt-II system L-idonate transporter | TCSA-Cases | ko02000 Transporters | TCSA-Cases | None | None |
| K06159 | yojI | multidrug/microcin transport system ATP-binding/permease protein | TCSA-Cases | ko02000 Transporters | TCSA-Cases | None | None |
| K06197 | chaB | cation transport regulator | TCSA-Cases | None | TCSA-Cases | None | None |
| K06222 | dkgB | 2,5-diketo-D-gluconate reductase B [EC:1.1.1.346] | TCSA-Cases | ko01000 Enzymes | TCSA-Cases | None | None |
| K06858 | btuF | vitamin B12 transport system substrate-binding protein | TCSA-Cases | ko02000 Transporters | TCSA-Cases | None | None |
| K06946 | K06946 | uncharacterized protein | TCSA-Cases | None | TCSA-Cases | None | None |
| K07038 | K07038 | inner membrane protein | TCSA-Cases | None | TCSA-Cases | None | None |
| K07136 | K07136 | uncharacterized protein | TCSA-Cases | None | TCSA-Cases | None | None |
| K07156 | copC, pcoC | copper resistance protein C | TCSA-Cases | ko02000 Transporters | TCSA-Cases | None | None |
| K07229 | yqjH | ferric-chelate reductase (NADPH) [EC:1.16.1.9] | TCSA-Cases | ko01000 Enzymes | TCSA-Cases | None | None |
| K07245 | pcoD | copper resistance protein D | TCSA-Cases | ko02000 Transporters | TCSA-Cases | None | None |
| K07251 | thiK | thiamine kinase [EC:2.7.1.89] | TCSA-Cases | ko01000 Enzymes | TCSA-Cases | None | None |
| K07264 | arnT, pmrK | 4-amino-4-deoxy-L-arabinose transferase [EC:2.4.2.43] | TCSA-Cases | ko01000 Enzymes | TCSA-Cases | None | None |
| K07269 | ytfB | cell division protein YtfB | TCSA-Cases | ko03036 Chromosome | TCSA-Cases | None | None |
| K07283 | ydiY | putative salt-induced outer membrane protein | TCSA-Cases | None | TCSA-Cases | None | None |
| K07345 | fimA | major type 1 subunit fimbrin (pilin) | TCSA-Cases | ko02044 Secretion | TCSA-Cases | None | None |
| K07346 | fimC | fimbrial chaperone protein | TCSA-Cases | ko02044 Secretion | TCSA-Cases | None | None |
| K07470 | sbmC | DNA gyrase inhibitor | TCSA-Cases | None | TCSA-Cases | None | None |
| K07637 | phoQ | two-component system, OmpR family, sensor histidine kinase PhoQ [EC:2.7.13.3] | TCSA-Cases | ko01000 Enzymes | TCSA-Cases | None | None |
| K07643 | basS | two-component system, OmpR family, sensor histidine kinase BasS [EC:2.7.13.3] | TCSA-Cases | ko01000 Enzymes | TCSA-Cases | None | None |
| K07660 | phoP | two-component system, OmpR family, response regulator PhoP | TCSA-Cases | ko02022 Two-component | TCSA-Cases | None | None |
| K07675 | uhpB | two-component system, NarL family, sensor histidine kinase UhpB [EC:2.7.13.3] | TCSA-Cases | ko01000 Enzymes | TCSA-Cases | None | None |
| K07676 | rcsD | two-component system, NarL family, sensor histidine kinase RcsD [EC:2.7.13.3] | TCSA-Cases | ko01000 Enzymes | TCSA-Cases | None | None |
| K07677 | rcsC | two-component system, NarL family, capsular synthesis sensor histidine kinase RcsC [EC:2.7.13.3] | TCSA-Cases | ko01000 Enzymes | TCSA-Cases | None | None |
| K07686 | uhpA | two-component system, NarL family, uhpT operon response regulator UhpA | TCSA-Cases | ko02022 Two-component | TCSA-Cases | None | None |
| K07689 | uvrY, gacA, varA | two-component system, NarL family, invasion response regulator UvrY | TCSA-Cases | ko02022 Two-component | TCSA-Cases | None | None |
| K07703 | dcuR | two-component system, CitB family, response regulator DcuR | TCSA-Cases | ko02022 Two-component | TCSA-Cases | None | None |
| K07724 | ner, nlp, sfsB | Ner family transcriptional regulator | TCSA-Cases | ko03000 Transcription | TCSA-Cases | None | None |
| K07781 | rcsA | LuxR family transcriptional regulator, capsular biosynthesis positive transcription factor | TCSA-Cases | ko03000 Transcription | TCSA-Cases | None | None |
| K07784 | uhpT | MFS transporter, OPA family, hexose phosphate transport protein UhpT | TCSA-Cases | ko02000 Transporters | TCSA-Cases | None | None |
| K07795 | tctC | putative tricarboxylic transport membrane protein | TCSA-Cases | ko02000 Transporters | TCSA-Cases | None | None |
| K07796 | cusC, silC | outer membrane protein, copper/silver efflux system | TCSA-Cases | ko02000 Transporters | TCSA-Cases | None | None |
| K08137 | galP | MFS transporter, SP family, galactose:H+ symporter | TCSA-Cases | ko02000 Transporters | TCSA-Cases | None | None |
| K08154 | emrD | MFS transporter, DHA1 family, 2-module integral membrane pump EmrD | TCSA-Cases | ko02000 Transporters | TCSA-Cases | None | None |
| K08160 | mdfA, cmr | MFS transporter, DHA1 family, multidrug/chloramphenicol efflux transport protein | TCSA-Cases | ko02000 Transporters | TCSA-Cases | None | None |
| K08172 | shiA | MFS transporter, MHS family, shikimate and dehydroshikimate transport protein | TCSA-Cases | ko02000 Transporters | TCSA-Cases | None | None |
| K08219 | UMF2 | MFS transporter, UMF2 family, putative MFS family transporter protein | TCSA-Cases | ko02000 Transporters | TCSA-Cases | None | None |
| K08225 | entS | MFS transporter, ENTS family, enterobactin (siderophore) exporter | TCSA-Cases | ko02000 Transporters | TCSA-Cases | None | None |
| K08227 | lplT | MFS transporter, LPLT family, lysophospholipid transporter | TCSA-Cases | ko02000 Transporters | TCSA-Cases | None | None |
| K08308 | mltE, emtA | membrane-bound lytic murein transglycosylase E [EC:4.2.2.-] | TCSA-Cases | ko01000 Enzymes | TCSA-Cases | None | None |
| K08324 | sad | succinate-semialdehyde dehydrogenase [EC:1.2.1.16 1.2.1.24] | TCSA-Cases | ko01000 Enzymes | TCSA-Cases | None | None |
| K08348 | fdnG | formate dehydrogenase-N, alpha subunit [EC:1.17.5.3] | TCSA-Cases | ko01000 Enzymes | TCSA-Cases | None | None |
| K08349 | fdnH | formate dehydrogenase-N, beta subunit | TCSA-Cases | ko02000 Transporters | TCSA-Cases | None | None |
| K08350 | fdnI | formate dehydrogenase-N, gamma subunit | TCSA-Cases | ko02000 Transporters | TCSA-Cases | None | None |
| K08485 | ptsO, npr | phosphocarrier protein NPr | TCSA-Cases | ko02000 Transporters | TCSA-Cases | None | None |
| K08682 | acpH | acyl carrier protein phosphodiesterase [EC:3.1.4.14] | TCSA-Cases | ko01000 Enzymes | TCSA-Cases | None | None |
| K08723 | yjjG | pyrimidine 5'-nucleotidase [EC:3.1.3.-] | TCSA-Cases | ko01000 Enzymes | TCSA-Cases | None | None |
| K08970 | rcnA | nickel/cobalt transporter (NicO) family protein | TCSA-Cases | ko02000 Transporters | TCSA-Cases | None | None |
| K08997 | SELENOO, selO | protein adenylyltransferase [EC:2.7.7.108] | TCSA-Cases | ko01000 Enzymes | TCSA-Cases | None | None |
| K09161 | K09161 | uncharacterized protein | TCSA-Cases | None | TCSA-Cases | None | None |
| K09251 | patA | putrescine aminotransferase [EC:2.6.1.82] | TCSA-Cases | ko01000 Enzymes | None | None | None |
| K09912 | K09912 | uncharacterized protein | TCSA-Cases | None | None | None | None |
| K09914 | K09914 | putative lipoprotein | TCSA-Cases | None | None | None | None |
| K09918 | K09918 | uncharacterized protein | TCSA-Cases | None | None | None | None |
| K09920 | K09920 | uncharacterized protein | TCSA-Cases | None | None | None | None |
| K10015 | hisM | histidine transport system permease protein | TCSA-Cases | ko02000 Transporters | None | None | None |
| K10016 | hisQ | histidine transport system permease protein | TCSA-Cases | ko02000 Transporters | None | None | None |
| K10017 | hisP | histidine transport system ATP-binding protein [EC:7.4.2.1] | TCSA-Cases | ko01000 Enzymes | None | None | None |
| K10111 | malK, mtlK, thuK | multiple sugar transport system ATP-binding protein [EC:7.5.2.-] | TCSA-Cases | ko02000 Transporters | None | None | None |
| K10748 | tus, tau | DNA replication terminus site-binding protein | TCSA-Cases | ko03032 DNA | None | None | None |
| K10831 | tauB | taurine transport system ATP-binding protein [EC:7.6.2.7] | TCSA-Cases | ko01000 Enzymes | None | None | None |
| K10857 | exoX | exodeoxyribonuclease X [EC:3.1.11.-] | TCSA-Cases | ko01000 Enzymes | None | None | None |
| K11201 | frvA | fructose-like PTS system EIIA component [EC:2.7.1.-] | TCSA-Cases | ko01000 Enzymes | None | None | None |
| K11208 | yncG | GST-like protein | TCSA-Cases | None | None | None | None |
| K11209 | yghU, yfcG | GSH-dependent disulfide-bond oxidoreductase [EC:1.8.4.-] | TCSA-Cases | ko01000 Enzymes | None | None | None |
| K11391 | rlmG | 23S rRNA (guanine1835-N2)-methyltransferase [EC:2.1.1.174] | TCSA-Cases | ko01000 Enzymes | None | None | None |
| K11472 | glcE | glycolate dehydrogenase FAD-binding subunit [EC:1.1.99.14] | TCSA-Cases | ko01000 Enzymes | None | None | None |
| K11531 | lsrR | lsr operon transcriptional repressor | TCSA-Cases | ko03000 Transcription | None | None | None |
| K11735 | gabP | GABA permease | TCSA-Cases | ko02000 Transporters | None | None | None |
| K11736 | proY | proline-specific permease ProY | TCSA-Cases | ko02000 Transporters | None | None | None |
| K11745 | kefC | glutathione-regulated potassium-efflux system ancillary protein KefC | TCSA-Cases | ko02000 Transporters | None | None | None |
| K11746 | kefF | glutathione-regulated potassium-efflux system ancillary protein KefF | TCSA-Cases | ko02000 Transporters | None | None | None |
| K11896 | impG, vasA | type VI secretion system protein ImpG | TCSA-Cases | ko02044 Secretion | None | None | None |
| K11898 | impE | type VI secretion system protein ImpE | TCSA-Cases | ko02044 Secretion | None | None | None |
| K11923 | cueR | MerR family transcriptional regulator, copper efflux regulator | TCSA-Cases | ko03000 Transcription | None | None | None |
| K11924 | mntR | DtxR family transcriptional regulator, manganese transport regulator | TCSA-Cases | ko03000 Transcription | None | None | None |
| K11929 | phoE | outer membrane pore protein E | TCSA-Cases | ko02000 Transporters | None | None | None |
| K11938 | cof | HMP-PP phosphatase [EC:3.6.1.-] | TCSA-Cases | ko01000 Enzymes | None | None | None |
| K12147 | msyB | acidic protein MsyB | TCSA-Cases | None | None | None | None |
| K12148 | bssS | biofilm regulator BssS | TCSA-Cases | ko02048 Prokaryotic | None | None | None |
| K12151 | bhsA | multiple stress resistance protein BhsA | TCSA-Cases | ko02048 Prokaryotic | None | None | None |
| K12288 | hofM | pilus assembly protein HofM | TCSA-Cases | ko02044 Secretion | None | None | None |
| K12289 | hofN | pilus assembly protein HofN | TCSA-Cases | ko02044 Secretion | None | None | None |
| K12290 | hofO | pilus assembly protein HofO | TCSA-Cases | ko02044 Secretion | None | None | None |
| K12291 | hofP | pilus assembly protein HofP | TCSA-Cases | ko02044 Secretion | None | None | None |
| K12500 | tesC | thioesterase III [EC:3.1.2.-] | TCSA-Cases | ko01000 Enzymes | None | None | None |
| K12525 | metL | bifunctional aspartokinase / homoserine dehydrogenase 2 [EC:2.7.2.4 1.1.1.3] | TCSA-Cases | ko01000 Enzymes | None | None | None |
| K12582 | wecF, rffT | dTDP-N-acetylfucosamine:lipid II N-acetylfucosaminyltransferase [EC:2.4.1.325] | TCSA-Cases | ko01000 Enzymes | None | None | None |
| K12686 | apeE, estA, lip-1 | outer membrane lipase/esterase | TCSA-Cases | ko02000 Transporters | None | None | None |
| K12945 | nudK | GDP-mannose pyrophosphatase NudK [EC:3.6.1.-] | TCSA-Cases | ko01000 Enzymes | None | None | None |
| K12957 | ahr | alcohol/geraniol dehydrogenase (NADP+) [EC:1.1.1.2 1.1.1.183] | TCSA-Cases | ko01000 Enzymes | None | None | None |
| K12963 | arnF | undecaprenyl phosphate-alpha-L-ara4N flippase subunit ArnF | TCSA-Cases | None | None | None | None |
| K12974 | lpxP | KDO2-lipid IV(A) palmitoleoyltransferase [EC:2.3.1.242] | TCSA-Cases | ko01000 Enzymes | None | None | None |
| K12975 | eptB | KDO II ethanolaminephosphotransferase [EC:2.7.8.42] | TCSA-Cases | ko01000 Enzymes | None | None | None |
| K13069 | E2.7.7.65 | diguanylate cyclase [EC:2.7.7.65] | TCSA-Cases | ko01000 Enzymes | None | None | None |
| K13255 | fhuF | ferric iron reductase protein FhuF | TCSA-Cases | None | None | None | None |
| K13301 | secM | secretion monitor | TCSA-Cases | ko02044 Secretion | None | None | None |
| K13620 | wcaD | putative colanic acid polymerase | TCSA-Cases | None | None | None | None |
| K13630 | marB | multiple antibiotic resistance protein MarB | TCSA-Cases | None | None | None | None |
| K13631 | soxS | AraC family transcriptional regulator, mar-sox-rob regulon activator | TCSA-Cases | ko03000 Transcription | None | None | None |
| K13632 | marA | AraC family transcriptional regulator, mar-sox-rob regulon activator | TCSA-Cases | ko03000 Transcription | None | None | None |
| K13638 | zntR | MerR family transcriptional regulator, Zn(II)-responsive regulator of zntA | TCSA-Cases | ko03000 Transcription | None | None | None |
| K13650 | mcbA | MqsR-controlled colanic acid and biofilm protein A | TCSA-Cases | ko02048 Prokaryotic | None | None | None |
| K13654 | mcbR | GntR family transcriptional regulator, colanic acid and biofilm gene transcriptional regulator | TCSA-Cases | ko03000 Transcription | None | None | None |
| K13684 | wcaC | putative colanic acid biosynthesis glycosyltransferase WcaC [EC:2.4.-.-] | TCSA-Cases | ko01000 Enzymes | None | None | None |
| K13918 | gudX | glucarate dehydratase-related protein | TCSA-Cases | None | None | None | None |
| K14054 | mpaA | murein peptide amidase A | TCSA-Cases | None | None | None | None |
| K14062 | ompN | outer membrane protein N | TCSA-Cases | ko02000 Transporters | None | None | None |
| K14348 | lldR | GntR family transcriptional regulator, L-lactate dehydrogenase operon regulator | TCSA-Cases | ko03000 Transcription | None | None | None |
| K14588 | cueO | cuproxidase [EC:1.16.3.4] | TCSA-Cases | ko01000 Enzymes | None | None | None |
| K14762 | yibL | ribosome-associated protein | TCSA-Cases | ko03009 Ribosome | None | None | None |
| K15548 | aaeA | p-hydroxybenzoic acid efflux pump subunit AaeA | TCSA-Cases | ko02000 Transporters | None | None | None |
| K15552 | tauC | taurine transport system permease protein | TCSA-Cases | ko02000 Transporters | None | None | None |
| K15722 | cedA | cell division activator | TCSA-Cases | None | None | None | None |
| K15723 | syd | SecY interacting protein Syd | TCSA-Cases | None | None | None | None |
| K15827 | hycB | formate hydrogenlyase subunit 2 | TCSA-Cases | None | None | None | None |
| K15828 | hycC | formate hydrogenlyase subunit 3 | TCSA-Cases | None | None | None | None |
| K15829 | hycD | formate hydrogenlyase subunit 4 | TCSA-Cases | None | None | None | None |
| K15830 | hycE | formate hydrogenlyase subunit 5 | TCSA-Cases | None | None | None | None |
| K15831 | hycF | formate hydrogenlyase subunit 6 | TCSA-Cases | None | None | None | None |
| K15832 | hycG | formate hydrogenlyase subunit 7 | TCSA-Cases | None | None | None | None |
| K15833 | hycA | formate hydrogenlyase regulatory protein HycA | TCSA-Cases | None | None | None | None |
| K15834 | hycH | formate hydrogenlyase maturation protein HycH | TCSA-Cases | None | None | None | None |
| K15974 | emrR, mprA | MarR family transcriptional regulator, negative regulator of the multidrug operon emrRAB | TCSA-Cases | ko03000 Transcription | None | None | None |
| K16693 | wzxE | enterobacterial common antigen flippase | TCSA-Cases | ko02000 Transporters | None | None | None |
| K16695 | wzxC | lipopolysaccharide exporter | TCSA-Cases | ko02000 Transporters | None | None | None |
| K16703 | wcaL, amsK, cpsK | colanic acid/amylovoran/stewartan biosynthesis glycosyltransferase WcaL/AmsK/CpsK [EC:2.4.-.-] | TCSA-Cases | ko01000 Enzymes | None | None | None |
| K16704 | rffC, wecD | dTDP-4-amino-4,6-dideoxy-D-galactose acyltransferase [EC:2.3.1.210] | TCSA-Cases | ko01000 Enzymes | None | None | None |
| K16711 | wcaM | colanic acid biosynthesis protein WcaM | TCSA-Cases | None | None | None | None |
| K17938 | sbmA, bacA | peptide/bleomycin uptake transporter | TCSA-Cases | ko02000 Transporters | None | None | None |
| K18141 | acrE | membrane fusion protein, multidrug efflux system | TCSA-Cases | ko02000 Transporters | None | None | None |
| K18142 | acrF | multidrug efflux pump | TCSA-Cases | ko02000 Transporters | None | None | None |
| K18324 | acrD | multidrug efflux pump | TCSA-Cases | ko02000 Transporters | None | None | None |
| K18326 | mdtD | MFS transporter, DHA2 family, multidrug resistance protein | TCSA-Cases | ko02000 Transporters | None | None | None |
| K18446 | ygiF | triphosphatase [EC:3.6.1.25] | TCSA-Cases | ko01000 Enzymes | None | None | None |
| K18697 | pgpC | phosphatidylglycerophosphatase C [EC:3.1.3.27] | TCSA-Cases | ko01000 Enzymes | None | None | None |
| K18765 | csrD | RNase E specificity factor CsrD | TCSA-Cases | ko03019 Messenger | None | None | None |
| K18968 | adrA | diguanylate cyclase [EC:2.7.7.65] | TCSA-Cases | ko01000 Enzymes | None | None | None |
| K18988 | ampH | serine-type D-Ala-D-Ala carboxypeptidase/endopeptidase [EC:3.4.16.4 3.4.21.-] | TCSA-Cases | ko01000 Enzymes | None | None | None |
| K19000 | rof | Rho-binding antiterminator | TCSA-Cases | ko03021 Transcription | None | None | None |
| K19046 | casB, cse2 | CRISPR system Cascade subunit CasB | TCSA-Cases | ko02048 Prokaryotic | None | None | None |
| K19048 | symE | toxic protein SymE | TCSA-Cases | ko02048 Prokaryotic | None | None | None |
| K19123 | casA, cse1 | CRISPR system Cascade subunit CasA | TCSA-Cases | ko02048 Prokaryotic | None | None | None |
| K19125 | casD, cse5 | CRISPR system Cascade subunit CasD | TCSA-Cases | ko02048 Prokaryotic | None | None | None |
| K19126 | casE, cse3 | CRISPR system Cascade subunit CasE | TCSA-Cases | ko02048 Prokaryotic | None | None | None |
| K19162 | tomB | hha toxicity modulator TomB | TCSA-Cases | ko02048 Prokaryotic | None | None | None |
| K19222 | menI, DHNAT | 1,4-dihydroxy-2-naphthoyl-CoA hydrolase [EC:3.1.2.28] | TCSA-Cases | ko01000 Enzymes | None | None | None |
| K19234 | ynhG | L,D-transpeptidase YnhG | TCSA-Cases | ko01002 Peptidases | None | None | None |
| K19235 | ybiS | L,D-transpeptidase YbiS | TCSA-Cases | ko01002 Peptidases | None | None | None |
| K19236 | ycfS | L,D-transpeptidase YcfS | TCSA-Cases | ko01002 Peptidases | None | None | None |
| K19270 | hxpA | mannitol-1-/sugar-/sorbitol-6-phosphatase [EC:3.1.3.22 3.1.3.23 3.1.3.50] | TCSA-Cases | ko01000 Enzymes | None | None | None |
| K19303 | mepH | murein DD-endopeptidase [EC:3.4.-.-] | TCSA-Cases | ko01000 Enzymes | None | None | None |
| K19354 | waaH | heptose III glucuronosyltransferase [EC:2.4.1.-] | TCSA-Cases | ko01000 Enzymes | None | None | None |
| K19611 | fepA, pfeA, iroN, pirA | ferric enterobactin receptor | TCSA-Cases | ko02000 Transporters | None | None | None |
| K19776 | dgoR | GntR family transcriptional regulator, galactonate operon transcriptional repressor | TCSA-Cases | ko03000 Transcription | None | None | None |
| K12530 | rtxB, fitA | ATP-binding cassette, subfamily B, bacterial RtxB | TCSA-Cases | ko02000 Transporters | None | None | None |
| K12531 | rtxE, fitC | ATP-binding cassette, subfamily B, bacterial RtxE | TCSA-Cases | ko02000 Transporters | None | None | None |
| K12532 | rtxD, fitB | membrane fusion protein, RTX toxin transport system | TCSA-Cases | ko02000 Transporters | None | None | None |
| K04063 | osmC, ohr | lipoyl-dependent peroxiredoxin [EC:1.11.1.28] | TCSA-Cases | ko01000 Enzymes | TCSA-Cases | None | None |
| K09023 | rutD | aminoacrylate hydrolase [EC:3.5.1.-] | TCSA-Cases | ko01000 Enzymes | TCSA-Cases | None | None |
| K07119 | PTGR3, ZADH2 | prostaglandin reductase 3 [EC:1.3.1.48] | TCSA-Cases | ko01000 Enzymes | TCSA-Cases | None | None |
| K00098 | idnD | L-idonate 5-dehydrogenase [EC:1.1.1.264] | TCSA-Cases | ko01000 Enzymes | None | None | None |
| K00140 | mmsA, iolA, ALDH6A1 | malonate-semialdehyde dehydrogenase (acetylating) / methylmalonate-semialdehyde dehydrogenase [EC:1.2.1.18 1.2.1.27] | TCSA-Cases | ko01000 Enzymes | None | None | None |
| K00480 | E1.14.13.1 | salicylate hydroxylase [EC:1.14.13.1] | TCSA-Cases | ko01000 Enzymes | None | None | None |
| K00483 | hpaB | 4-hydroxyphenylacetate 3-monooxygenase [EC:1.14.14.9] | TCSA-Cases | ko01000 Enzymes | None | None | None |
| K00632 | fadA, fadI | acetyl-CoA acyltransferase [EC:2.3.1.16] | TCSA-Cases | ko01000 Enzymes | None | None | None |
| K00758 | deoA, TYMP | thymidine phosphorylase [EC:2.4.2.4] | TCSA-Cases | ko01000 Enzymes | None | None | None |
| K01684 | dgoD | galactonate dehydratase [EC:4.2.1.6] | TCSA-Cases | ko01000 Enzymes | None | None | None |
| K01725 | cynS | cyanate lyase [EC:4.2.1.104] | TCSA-Cases | ko01000 Enzymes | None | None | None |
| K01823 | idi, IDI | isopentenyl-diphosphate Delta-isomerase [EC:5.3.3.2] | TCSA-Cases | ko01000 Enzymes | None | None | None |
| K01974 | RTCA, rtcA | RNA 3'-terminal phosphate cyclase (ATP) [EC:6.5.1.4] | TCSA-Cases | ko01000 Enzymes | None | None | None |
| K02041 | phnC | phosphonate transport system ATP-binding protein [EC:7.3.2.2] | TCSA-Cases | ko01000 Enzymes | None | None | None |
| K02100 | araE | MFS transporter, SP family, arabinose:H+ symporter | TCSA-Cases | ko02000 Transporters | None | None | None |
| K02363 | entE, dhbE, vibE, mxcE | 2,3-dihydroxybenzoate---[aryl-carrier protein] ligase [EC:6.3.2.14 6.2.1.71] | TCSA-Cases | ko01000 Enzymes | None | None | None |
| K02391 | flgF | flagellar basal-body rod protein FlgF | TCSA-Cases | ko02035 Bacterial | None | None | None |
| K02466 | gutM | glucitol operon activator protein | TCSA-Cases | None | None | None | None |
| K02467 | gutQ | arabinose 5-phosphate isomerase [EC:5.3.1.13] | TCSA-Cases | ko01000 Enzymes | None | None | None |
| K02468 | srlR, gutR | DeoR family transcriptional regulator, glucitol operon repressor | TCSA-Cases | ko03000 Transcription | None | None | None |
| K02562 | mtlR | mannitol operon repressor | TCSA-Cases | ko03000 Transcription | None | None | None |
| K02753 | ascF | beta-glucoside (arbutin/salicin/cellobiose) PTS system EIICB component [EC:2.7.1.-] | TCSA-Cases | ko01000 Enzymes | None | None | None |
| K02850 | waaY, rfaY | heptose II phosphotransferase [EC:2.7.1.-] | TCSA-Cases | ko01000 Enzymes | None | None | None |
| K03207 | gmm, nudD, wcaH | GDP-mannose mannosyl hydrolase [EC:3.6.1.-] | TCSA-Cases | ko01000 Enzymes | None | None | None |
| K03230 | yscV, sctV, hrcV, ssaV, invA | type III secretion protein V | TCSA-Cases | ko02044 Secretion | None | None | None |
| K03288 | citA, tcuC | MFS transporter, MHS family, citrate/tricarballylate:H+ symporter | TCSA-Cases | ko02000 Transporters | None | None | None |
| K03485 | treR | LacI family transcriptional regulator, trehalose operon repressor | TCSA-Cases | ko03000 Transcription | None | None | None |
| K03490 | chbR, celD | AraC family transcriptional regulator, dual regulator of chb operon | TCSA-Cases | ko03000 Transcription | None | None | None |
| K03535 | gudP | MFS transporter, ACS family, glucarate transporter | TCSA-Cases | ko02000 Transporters | None | None | None |
| K03623 | yhcO | ribonuclease inhibitor | TCSA-Cases | None | None | None | None |
| K03825 | aaaT | L-phenylalanine/L-methionine N-acetyltransferase [EC:2.3.1.53 2.3.1.-] | TCSA-Cases | ko01000 Enzymes | None | None | None |
| K04081 | ibpB | molecular chaperone IbpB | TCSA-Cases | ko03110 Chaperones | None | None | None |
| K04338 | csgF | curli production assembly/transport component CsgF | TCSA-Cases | ko02044 Secretion | None | None | None |
| K04750 | phnB | PhnB protein | TCSA-Cases | None | None | None | None |
| K05775 | malM | maltose operon periplasmic protein | TCSA-Cases | None | None | None | None |
| K05798 | leuO | LysR family transcriptional regulator, transcriptional activator for leuABCD operon | TCSA-Cases | ko03000 Transcription | None | None | None |
| K05804 | rob | AraC family transcriptional regulator, mar-sox-rob regulon activator | TCSA-Cases | ko03000 Transcription | None | None | None |
| K05812 | DTWD2, tapT | tRNA-uridine aminocarboxypropyltransferase [EC:2.5.1.25] | TCSA-Cases | ko01000 Enzymes | None | None | None |
| K05835 | rhtC | threonine efflux protein | TCSA-Cases | ko02000 Transporters | None | None | None |
| K05876 | trg | methyl-accepting chemotaxis protein III, ribose and galactose sensor receptor | TCSA-Cases | ko02035 Bacterial | None | None | None |
| K05880 | dhaR | transcriptional activator for dhaKLM operon | TCSA-Cases | ko03000 Transcription | None | None | None |
| K06156 | gntU | Gnt-I system low-affinity gluconate transporter | TCSA-Cases | ko02000 Transporters | None | None | None |
| K06214 | csgG | curli production assembly/transport component CsgG | TCSA-Cases | ko02044 Secretion | None | None | None |
| K06884 | K06884 | uncharacterized protein | TCSA-Cases | None | None | None | None |
| K06887 | K06887 | uncharacterized protein | TCSA-Cases | None | None | None | None |
| K06970 | rlmF | 23S rRNA (adenine1618-N6)-methyltransferase [EC:2.1.1.181] | TCSA-Cases | ko01000 Enzymes | None | None | None |
| K07026 | E3.1.3.70 | mannosyl-3-phosphoglycerate phosphatase [EC:3.1.3.70] | TCSA-Cases | ko01000 Enzymes | None | None | None |
| K07146 | K07146 | UPF0176 protein | TCSA-Cases | None | None | None | None |
| K07337 | K07337 | penicillin-binding protein activator | TCSA-Cases | None | None | None | None |
| K07347 | fimD, fimC, mrkC, htrE, cssD | outer membrane usher protein | TCSA-Cases | ko02000 Transporters | None | None | None |
| K07348 | fimF | minor fimbrial subunit | TCSA-Cases | ko02044 Secretion | None | None | None |
| K07490 | feoC | ferrous iron transport protein C | TCSA-Cases | ko02000 Transporters | None | None | None |
| K07642 | baeS, smeS | two-component system, OmpR family, sensor histidine kinase BaeS [EC:2.7.13.3] | TCSA-Cases | ko01000 Enzymes | None | None | None |
| K08276 | eco | ecotin | TCSA-Cases | ko01002 Peptidases | None | None | None |
| K08313 | fsaA, mipB | fructose-6-phosphate aldolase 1 [EC:4.1.2.-] | TCSA-Cases | ko01000 Enzymes | None | None | None |
| K08318 | yihU | 4-hydroxybutyrate dehydrogenase / sulfolactaldehyde 3-reductase [EC:1.1.1.61 1.1.1.373] | TCSA-Cases | ko01000 Enzymes | None | None | None |
| K08320 | nudG | (d)CTP diphosphatase [EC:3.6.1.65] | TCSA-Cases | ko01000 Enzymes | None | None | None |
| K08322 | rspB | L-gulonate 5-dehydrogenase [EC:1.1.1.380] | TCSA-Cases | ko01000 Enzymes | None | None | None |
| K08326 | ypdF | aminopeptidase [EC:3.4.11.-] | TCSA-Cases | ko01000 Enzymes | None | None | None |
| K09982 | K09982 | uncharacterized protein | TCSA-Cases | None | None | None | None |
| K10011 | arnA, pmrI | UDP-4-amino-4-deoxy-L-arabinose formyltransferase / UDP-glucuronic acid dehydrogenase (UDP-4-keto-hexauronic acid decarboxylating) [EC:2.1.2.13 1.1.1.305] | TCSA-Cases | ko01000 Enzymes | None | None | None |
| K10537 | araF | L-arabinose transport system substrate-binding protein | TCSA-Cases | ko02000 Transporters | None | None | None |
| K10555 | lsrB | AI-2 transport system substrate-binding protein | TCSA-Cases | ko02000 Transporters | None | None | None |
| K10558 | lsrA, ego | AI-2 transport system ATP-binding protein | TCSA-Cases | ko02000 Transporters | None | None | None |
| K11102 | gltP, gltT | proton glutamate symport protein | TCSA-Cases | ko02000 Transporters | None | None | None |
| K11530 | lsrG | (4S)-4-hydroxy-5-phosphonooxypentane-2,3-dione isomerase [EC:5.3.1.32] | TCSA-Cases | ko01000 Enzymes | None | None | None |
| K11734 | aroP | aromatic amino acid transport protein AroP | TCSA-Cases | ko02000 Transporters | None | None | None |
| K11744 | tqsA | AI-2 transport protein TqsA | TCSA-Cases | None | None | None | None |
| K11894 | impI, vasC | type VI secretion system protein ImpI | TCSA-Cases | ko02044 Secretion | None | None | None |
| K11895 | impH, vasB | type VI secretion system protein ImpH | TCSA-Cases | ko02044 Secretion | None | None | None |
| K11909 | vasI | type VI secretion system protein VasI | TCSA-Cases | ko02044 Secretion | None | None | None |
| K12265 | norW | nitric oxide reductase FlRd-NAD(+) reductase [EC:1.18.1.-] | TCSA-Cases | ko01000 Enzymes | None | None | None |
| K12687 | flu | antigen 43 | TCSA-Cases | ko02000 Transporters | None | None | None |
| K12943 | ygeR | lipoprotein YgeR | TCSA-Cases | None | None | None | None |
| K12944 | nudI | nucleoside triphosphatase [EC:3.6.1.-] | TCSA-Cases | ko01000 Enzymes | None | None | None |
| K14052 | puuP | putrescine importer | TCSA-Cases | ko02000 Transporters | None | None | None |
| K14053 | ompG | outer membrane protein G | TCSA-Cases | ko02000 Transporters | None | None | None |
| K14059 | int | integrase | TCSA-Cases | None | None | None | None |
| K14061 | uspF | universal stress protein F | TCSA-Cases | None | None | None | None |
| K14064 | uspC | universal stress protein C | TCSA-Cases | None | None | None | None |
| K14414 | rtcR | transcriptional regulatory protein RtcR | TCSA-Cases | ko03000 Transcription | None | None | None |
| K15540 | ecpD | chaperone protein EcpD | TCSA-Cases | None | None | None | None |
| K15922 | yihQ | sulfoquinovosidase [EC:3.2.1.199] | TCSA-Cases | ko01000 Enzymes | None | None | None |
| K16263 | yjeH | amino acid efflux transporter | TCSA-Cases | ko02000 Transporters | None | None | None |
| K18140 | envR, acrS | TetR/AcrR family transcriptional regulator, acrEF/envCD operon repressor | TCSA-Cases | ko03000 Transcription | None | None | None |
| K18325 | ramA | AraC family of transcriptional regulator, multidrug resistance transcriptional activator | TCSA-Cases | ko03000 Transcription | None | None | None |
| K18478 | yihV | sulfofructose kinase [EC:2.7.1.184] | TCSA-Cases | ko01000 Enzymes | None | None | None |
| K19168 | cptA | toxin CptA | TCSA-Cases | ko02048 Prokaryotic | None | None | None |
| K19304 | mepM | murein DD-endopeptidase [EC:3.4.24.-] | TCSA-Cases | ko01000 Enzymes | None | None | None |
| K01406 | prtC | serralysin [EC:3.4.24.40] | TCSA-Cases | ko01000 Enzymes | TCSA-Cases | None | None |
| K09016 | rutG | putative pyrimidine permease RutG | TCSA-Cases | ko02000 Transporters | TCSA-Cases | None | None |
| K09018 | rutA | pyrimidine oxygenase [EC:1.14.99.46] | TCSA-Cases | ko01000 Enzymes | TCSA-Cases | None | None |
| K09020 | rutB | ureidoacrylate peracid hydrolase [EC:3.5.1.110] | TCSA-Cases | ko01000 Enzymes | TCSA-Cases | None | None |
| K09021 | rutC | aminoacrylate peracid reductase | TCSA-Cases | None | TCSA-Cases | None | None |
| K09932 | K09932 | uncharacterized protein | TCSA-Cases | None | TCSA-Cases | None | None |
| K10539 | araG | L-arabinose transport system ATP-binding protein [EC:7.5.2.12] | TCSA-Cases | ko01000 Enzymes | TCSA-Cases | None | None |
| K12536 | hasD, prtD, aprD, rsaD, prsD, eexD | ATP-binding cassette, subfamily C, type I secretion system permease/ATPase | TCSA-Cases | ko02000 Transporters | TCSA-Cases | None | None |
| K12537 | hasE, prtE, rsaE, prsE, eexE | membrane fusion protein, type I secretion system | TCSA-Cases | ko02000 Transporters | TCSA-Cases | None | None |
| K12538 | hasF, prtF | outer membrane protein, type I secretion system | TCSA-Cases | ko02000 Transporters | TCSA-Cases | None | None |
| K02464 | gspO | general secretion pathway protein O [EC:3.4.23.43 2.1.1.-] | TCSA-Cases | ko01000 Enzymes | None | None | None |
| K02465 | gspS | general secretion pathway protein S | TCSA-Cases | ko02044 Secretion | TCSA-Cases | None | None |
| K12206 | icmB, dotO | intracellular multiplication protein IcmB [EC:7.2.4.8] | TCSA-Cases | ko02044 Secretion | TCSA-Cases | None | None |
| K16234 | hutT | histidine transporter | TCSA-Cases | ko02000 Transporters | TCSA-Cases | None | None |
| K00311 | ETFDH | electron-transferring-flavoprotein dehydrogenase [EC:1.5.5.1] | TCSA-Cases | ko01000 Enzymes | None | None | None |
| K00675 | nhoA | N-hydroxyarylamine O-acetyltransferase [EC:2.3.1.118] | TCSA-Cases | ko01000 Enzymes | None | None | None |
| K01531 | mgtA, mgtB | P-type Mg2+ transporter [EC:7.2.2.14] | TCSA-Cases | ko01000 Enzymes | None | None | None |
| K01671 | yihT | sulfofructosephosphate aldolase [EC:4.1.2.57] | TCSA-Cases | ko01000 Enzymes | None | None | None |
| K01708 | garD | galactarate dehydratase [EC:4.2.1.42] | TCSA-Cases | ko01000 Enzymes | None | None | None |
| K01816 | hyi, gip | hydroxypyruvate isomerase [EC:5.3.1.22] | TCSA-Cases | ko01000 Enzymes | None | None | None |
| K01910 | citC | [citrate (pro-3S)-lyase] ligase [EC:6.2.1.22] | TCSA-Cases | ko01000 Enzymes | None | None | None |
| K02048 | cysP | sulfate/thiosulfate transport system substrate-binding protein | TCSA-Cases | ko02000 Transporters | None | None | None |
| K02791 | malX | maltose/glucose PTS system EIICB component [EC:2.7.1.199 2.7.1.208] | TCSA-Cases | ko01000 Enzymes | None | None | None |
| K02848 | waaP, rfaP | lipopolysaccharide core heptose(I) kinase [EC:2.7.1.235] | TCSA-Cases | ko01000 Enzymes | None | None | None |
| K03181 | ubiC | chorismate lyase [EC:4.1.3.40] | TCSA-Cases | ko01000 Enzymes | None | None | None |
| K03220 | yscD, sctD, ssaD | type III secretion protein D | TCSA-Cases | ko02044 Secretion | None | None | None |
| K03222 | yscJ, sctJ, hrcJ, ssaJ | type III secretion protein J | TCSA-Cases | ko02044 Secretion | None | None | None |
| K03225 | yscQ, sctQ, hrcQ, ssaQ, spaO | type III secretion protein Q | TCSA-Cases | ko02044 Secretion | None | None | None |
| K03226 | yscR, sctR, hrcR, ssaR | type III secretion protein R | TCSA-Cases | ko02044 Secretion | None | None | None |
| K03227 | yscS, sctS, hrcS, ssaS | type III secretion protein S | TCSA-Cases | ko02044 Secretion | None | None | None |
| K03229 | yscU, sctU, hrcU, ssaU | type III secretion protein U | TCSA-Cases | ko02044 Secretion | None | None | None |
| K03305 | TC.POT | proton-dependent oligopeptide transporter, POT family | TCSA-Cases | None | None | None | None |
| K03445 | nepI | MFS transporter, DHA1 family, purine ribonucleoside efflux pump | TCSA-Cases | ko02000 Transporters | None | None | None |
| K03482 | yidP | GntR family transcriptional regulator, glv operon transcriptional regulator | TCSA-Cases | ko03000 Transcription | None | None | None |
| K03755 | adiY | AraC family transcriptional regulator, transcriptional activator of adiA | TCSA-Cases | ko03000 Transcription | None | None | None |
| K03759 | adiC | arginine:agmatine antiporter | TCSA-Cases | ko02000 Transporters | None | None | None |
| K04334 | csgA | major curlin subunit | TCSA-Cases | ko02044 Secretion | None | None | None |
| K05522 | nei | endonuclease VIII [EC:3.2.2.- 4.2.99.18] | TCSA-Cases | ko01000 Enzymes | None | None | None |
| K05800 | ybaO | Lrp/AsnC family transcriptional regulator | TCSA-Cases | ko03000 Transcription | None | None | None |
| K06048 | gshA, ybdK | glutamate---cysteine ligase / carboxylate-amine ligase [EC:6.3.2.2 6.3.-.-] | TCSA-Cases | ko01000 Enzymes | None | None | None |
| K06075 | slyA | MarR family transcriptional regulator, transcriptional regulator for hemolysin | TCSA-Cases | ko03000 Transcription | None | None | None |
| K06132 | clsC | cardiolipin synthase C [EC:2.7.8.-] | TCSA-Cases | ko01000 Enzymes | None | None | None |
| K07224 | efeO | iron uptake system component EfeO | TCSA-Cases | ko02000 Transporters | None | None | None |
| K07350 | fimH | minor fimbrial subunit | TCSA-Cases | ko02044 Secretion | None | None | None |
| K07688 | fimZ | two-component system, NarL family, response regulator, fimbrial Z protein, FimZ | TCSA-Cases | ko02022 Two-component | None | None | None |
| K07757 | ybiV | sugar-phosphatase [EC:3.1.3.23] | TCSA-Cases | ko01000 Enzymes | None | None | None |
| K09927 | K09927 | uncharacterized protein | TCSA-Cases | None | None | None | None |
| K10012 | arnC, pmrF | undecaprenyl-phosphate 4-deoxy-4-formamido-L-arabinose transferase [EC:2.4.2.53] | TCSA-Cases | ko01000 Enzymes | None | None | None |
| K10545 | xylG | D-xylose transport system ATP-binding protein [EC:7.5.2.10] | TCSA-Cases | ko01000 Enzymes | None | None | None |
| K10556 | lsrC | AI-2 transport system permease protein | TCSA-Cases | ko02000 Transporters | None | None | None |
| K10557 | lsrD | AI-2 transport system permease protein | TCSA-Cases | ko02000 Transporters | None | None | None |
| K10972 | allS | LysR family transcriptional regulator, transcriptional activator of the allD operon | TCSA-Cases | ko03000 Transcription | None | None | None |
| K11738 | ansP | L-asparagine permease | TCSA-Cases | ko02000 Transporters | None | None | None |
| K11891 | impL, vasK, icmF | type VI secretion system protein ImpL | TCSA-Cases | ko02044 Secretion | None | None | None |
| K11893 | impJ, vasE | type VI secretion system protein ImpJ | TCSA-Cases | ko02044 Secretion | None | None | None |
| K11935 | pgaA | biofilm PGA synthesis protein PgaA | TCSA-Cases | None | None | None | None |
| K12264 | norV | anaerobic nitric oxide reductase flavorubredoxin | TCSA-Cases | None | None | None | None |
| K12299 | garP | MFS transporter, ACS family, probable galactarate transporter | TCSA-Cases | ko02000 Transporters | None | None | None |
| K12528 | xdhD | putative selenate reductase molybdopterin-binding subunit | TCSA-Cases | None | None | None | None |
| K12661 | LRA3, rhmD | L-rhamnonate dehydratase [EC:4.2.1.90] | TCSA-Cases | ko01000 Enzymes | None | None | None |
| K12979 | lpxO | beta-hydroxylase [EC:1.14.11.-] | TCSA-Cases | ko01000 Enzymes | None | None | None |
| K13637 | uxuR | GntR family transcriptional regulator, uxu operon transcriptional repressor | TCSA-Cases | ko03000 Transcription | None | None | None |
| K13641 | iclR | IclR family transcriptional regulator, acetate operon repressor | TCSA-Cases | ko03000 Transcription | None | None | None |
| K13890 | gsiC | glutathione transport system permease protein | TCSA-Cases | ko02000 Transporters | None | None | None |
| K16301 | efeB | deferrochelatase/peroxidase EfeB [EC:1.11.1.-] | TCSA-Cases | ko01000 Enzymes | None | None | None |
| K16321 | gntP | high-affinity gluconate transporter | TCSA-Cases | ko02000 Transporters | None | None | None |
| K16370 | pfkB | 6-phosphofructokinase 2 [EC:2.7.1.11] | TCSA-Cases | ko01000 Enzymes | None | None | None |
| K18837 | cbtA | cytoskeleton-binding toxin CbtA and related proteins | TCSA-Cases | ko02048 Prokaryotic | None | None | None |
| K18920 | hokA | protein HokA | TCSA-Cases | ko02048 Prokaryotic | None | None | None |
| K07178 | RIOK1 | RIO kinase 1 [EC:2.7.11.1] | TCSA-Cases | ko01000 Enzymes | None | None | None |
| K12203 | dotB, traJ | defect in organelle trafficking protein DotB [EC:7.2.4.8] | TCSA-Cases | ko02044 Secretion | None | None | None |
| K12213 | icmK, traN, dotH | intracellular multiplication protein IcmK | TCSA-Cases | ko02044 Secretion | None | None | None |
| K12214 | icmL, traM, dotI | intracellular multiplication protein IcmL | TCSA-Cases | ko02044 Secretion | None | None | None |
| K12217 | icmO, trbC, dotL | intracellular multiplication protein IcmO [EC:7.2.4.8] | TCSA-Cases | ko02044 Secretion | None | None | None |
| K13060 | lasI | acyl homoserine lactone synthase [EC:2.3.1.184] | TCSA-Cases | ko01000 Enzymes | None | None | None |
| K15547 | mdtO | multidrug resistance protein MdtO | TCSA-Cases | ko02000 Transporters | None | None | None |
| K15549 | mdtN | membrane fusion protein, multidrug efflux system | TCSA-Cases | ko02000 Transporters | None | None | None |
| K15550 | mdtP | outer membrane protein, multidrug efflux system | TCSA-Cases | ko02000 Transporters | None | None | None |
| K18029 | nicA | nicotinate dehydrogenase subunit A [EC:1.17.2.1] | TCSA-Cases | ko01000 Enzymes | None | None | None |
| K18555 | qnr, mcbG | fluoroquinolone resistance protein | TCSA-Cases | ko01504 Antimicrobial | None | None | None |
| K18900 | bpeT | LysR family transcriptional regulator, regulator for bpeEF and oprC | TCSA-Cases | ko03000 Transcription | None | None | None |
| K19734 | expR | LuxR family transcriptional regulator, quorum-sensing system regulator ExpR | TCSA-Cases | ko03000 Transcription | None | None | None |
| K03318 | tutB | tyrosine permease | TCSA-Cases | ko02000 Transporters | None | None | None |
| K17207 | xltA | putative xylitol transport system ATP-binding protein | TCSA-Cases | ko02000 Transporters | None | None | None |
| K19100 | blaDHA | beta-lactamase class C DHA [EC:3.5.2.6] | TCSA-Cases | ko01000 Enzymes | None | None | None |
| K11021 | tccC | insecticidal toxin complex protein TccC | TCSA-Cases | ko02042 Bacterial | None | None | None |
| K19063 | sat2 | streptothricin acetyltransferase [EC:2.3.-.-] | TCSA-Cases | ko01000 Enzymes | None | None | None |
| K00094 | E1.1.1.251, gatD | galactitol-1-phosphate 5-dehydrogenase [EC:1.1.1.251] | TCSA-Cases | ko01000 Enzymes | None | None | None |
| K00146 | feaB, tynC | phenylacetaldehyde dehydrogenase [EC:1.2.1.39] | TCSA-Cases | ko01000 Enzymes | None | None | None |
| K00249 | ACADM, acd | acyl-CoA dehydrogenase [EC:1.3.8.7] | TCSA-Cases | ko01000 Enzymes | None | None | None |
| K01160 | rusA | crossover junction endodeoxyribonuclease RusA [EC:3.1.21.10] | TCSA-Cases | ko01000 Enzymes | None | None | None |
| K01561 | dehH | haloacetate dehalogenase [EC:3.8.1.3] | TCSA-Cases | ko01000 Enzymes | None | None | None |
| K01584 | adiA | arginine decarboxylase [EC:4.1.1.19] | TCSA-Cases | ko01000 Enzymes | None | None | None |
| K01706 | gudD | glucarate dehydratase [EC:4.2.1.40] | TCSA-Cases | ko01000 Enzymes | None | None | None |
| K01857 | pcaB | 3-carboxy-cis,cis-muconate cycloisomerase [EC:5.5.1.2] | TCSA-Cases | ko01000 Enzymes | None | None | None |
| K02044 | phnD | phosphonate transport system substrate-binding protein | TCSA-Cases | ko02000 Transporters | None | None | None |
| K02046 | cysU | sulfate/thiosulfate transport system permease protein | TCSA-Cases | ko02000 Transporters | None | None | None |
| K02047 | cysW | sulfate/thiosulfate transport system permease protein | TCSA-Cases | ko02000 Transporters | None | None | None |
| K02381 | fdrA | FdrA protein | TCSA-Cases | None | None | None | None |
| K02509 | hpaH | 2-oxo-hept-3-ene-1,7-dioate hydratase [EC:4.2.1.-] | TCSA-Cases | ko01000 Enzymes | None | None | None |
| K03221 | yscF, sctF, ssaG, prgI | type III secretion protein F | TCSA-Cases | ko02044 Secretion | None | None | None |
| K03276 | waaR, waaT, rfaJ | UDP-glucose/galactose:(glucosyl)LPS alpha-1,2-glucosyl/galactosyltransferase [EC:2.4.1.-] | TCSA-Cases | ko01000 Enzymes | None | None | None |
| K03329 | yahN | amino acid exporter | TCSA-Cases | ko02000 Transporters | None | None | None |
| K04020 | eutD | phosphotransacetylase | TCSA-Cases | None | None | None | None |
| K04021 | eutE | aldehyde dehydrogenase | TCSA-Cases | None | None | None | None |
| K04022 | eutG | alcohol dehydrogenase | TCSA-Cases | None | None | None | None |
| K04025 | eutK | ethanolamine utilization protein EutK | TCSA-Cases | None | None | None | None |
| K04033 | eutR | AraC family transcriptional regulator, ethanolamine operon transcriptional activator | TCSA-Cases | ko03000 Transcription | None | None | None |
| K04093 | pheA1 | chorismate mutase [EC:5.4.99.5] | TCSA-Cases | ko01000 Enzymes | None | None | None |
| K05245 | caiT | L-carnitine/gamma-butyrobetaine antiporter | TCSA-Cases | ko02000 Transporters | None | None | None |
| K05847 | opuA | osmoprotectant transport system ATP-binding protein [EC:7.6.2.9] | TCSA-Cases | ko01000 Enzymes | None | None | None |
| K06146 | idnR, gntH | LacI family transcriptional regulator, gluconate utilization system Gnt-II transcriptional activator | TCSA-Cases | ko03000 Transcription | None | None | None |
| K06606 | iolI | 2-keto-myo-inositol isomerase [EC:5.3.99.11] | TCSA-Cases | ko01000 Enzymes | None | None | None |
| K06995 | K06995 | uncharacterized protein | TCSA-Cases | None | None | None | None |
| K07140 | K07140 | uncharacterized protein | TCSA-Cases | None | None | None | None |
| K07250 | gabT | 4-aminobutyrate aminotransferase / (S)-3-amino-2-methylpropionate transaminase / 5-aminovalerate transaminase [EC:2.6.1.19 2.6.1.22 2.6.1.48] | TCSA-Cases | ko01000 Enzymes | None | None | None |
| K07310 | ynfF | Tat-targeted selenate reductase subunit YnfF [EC:1.97.1.9] | TCSA-Cases | ko01000 Enzymes | None | None | None |
| K07314 | pphB | serine/threonine protein phosphatase 2 [EC:3.1.3.16] | TCSA-Cases | ko01000 Enzymes | None | None | None |
| K07356 | sfmH | fimbrial protein | TCSA-Cases | ko02035 Bacterial | None | None | None |
| K07647 | torS | two-component system, OmpR family, sensor histidine kinase TorS [EC:2.7.13.3] | TCSA-Cases | ko01000 Enzymes | None | None | None |
| K07690 | evgA, bvgA | two-component system, NarL family, response regulator EvgA | TCSA-Cases | ko02022 Two-component | None | None | None |
| K07772 | torR | two-component system, OmpR family, torCAD operon response regulator TorR | TCSA-Cases | ko02022 Two-component | None | None | None |
| K08167 | smvA, qacA, lfrA | MFS transporter, DHA2 family, multidrug resistance protein | TCSA-Cases | ko02000 Transporters | None | None | None |
| K08277 | caiF | transcriptional activator CaiF | TCSA-Cases | ko03000 Transcription | None | None | None |
| K08279 | caiE | carnitine operon protein CaiE | TCSA-Cases | None | None | None | None |
| K08299 | caiD | crotonobetainyl-CoA hydratase [EC:4.2.1.149] | TCSA-Cases | ko01000 Enzymes | None | None | None |
| K08317 | hcxA | hydroxycarboxylate dehydrogenase A [EC:1.1.1.-] | TCSA-Cases | ko01000 Enzymes | None | None | None |
| K08319 | ltnD | L-threonate 2-dehydrogenase [EC:1.1.1.411] | TCSA-Cases | ko01000 Enzymes | None | None | None |
| K08354 | phsC | thiosulfate reductase cytochrome b subunit | TCSA-Cases | ko02000 Transporters | None | None | None |
| K09712 | K09712 | uncharacterized protein | TCSA-Cases | None | None | None | None |
| K10973 | allR | IclR family transcriptional regulator, negative regulator of allantoin and glyoxylate utilization operons | TCSA-Cases | ko03000 Transcription | None | None | None |
| K11139 | hlyE, clyA, sheA | hemolysin E | TCSA-Cases | ko02042 Bacterial | None | None | None |
| K11216 | lsrK | autoinducer-2 kinase [EC:2.7.1.189] | TCSA-Cases | ko01000 Enzymes | None | None | None |
| K11892 | impK, ompA, vasF, dotU | type VI secretion system protein ImpK | TCSA-Cases | ko02044 Secretion | None | None | None |
| K11931 | pgaB | poly-beta-1,6-N-acetyl-D-glucosamine N-deacetylase [EC:3.5.1.-] | TCSA-Cases | ko01000 Enzymes | None | None | None |
| K12700 | rihC | non-specific riboncleoside hydrolase [EC:3.2.2.-] | TCSA-Cases | ko01000 Enzymes | None | None | None |
| K13891 | gsiD | glutathione transport system permease protein | TCSA-Cases | ko02000 Transporters | None | None | None |
| K13892 | gsiA | glutathione transport system ATP-binding protein | TCSA-Cases | ko02000 Transporters | None | None | None |
| K13953 | adhP | alcohol dehydrogenase, propanol-preferring [EC:1.1.1.1] | TCSA-Cases | ko01000 Enzymes | None | None | None |
| K14347 | SLC10A7, P7 | solute carrier family 10 (sodium/bile acid cotransporter), member 7 | TCSA-Cases | ko02000 Transporters | None | None | None |
| K15735 | csiR | GntR family transcriptional regulator, carbon starvation induced regulator | TCSA-Cases | ko03000 Transcription | None | None | None |
| K15736 | lhgO | (S)-2-hydroxyglutarate dehydrogenase [EC:1.1.5.13] | TCSA-Cases | ko01000 Enzymes | None | None | None |
| K15737 | csiD | glutarate dioxygenase [EC:1.14.11.64] | TCSA-Cases | ko01000 Enzymes | None | None | None |
| K15773 | hipB | HTH-type transcriptional regulator / antitoxin HipB | TCSA-Cases | ko03000 Transcription | None | None | None |
| K15777 | DODA | 4,5-DOPA dioxygenase extradiol [EC:1.13.11.-] | TCSA-Cases | ko01000 Enzymes | None | None | None |
| K16076 | nmpC, ompD | outer membrane porin protein LC | TCSA-Cases | ko02000 Transporters | None | None | None |
| K18838 | cbeA | cytoskeleton bundling-enhancing protein CbeA and related proteins | TCSA-Cases | ko02048 Prokaryotic | None | None | None |
| K18919 | hokC_D | protein HokC/D | TCSA-Cases | ko02048 Prokaryotic | None | None | None |
| K19124 | casC, cse4 | CRISPR system Cascade subunit CasC | TCSA-Cases | ko02048 Prokaryotic | None | None | None |
| K19238 | pmrD | signal transduction protein PmrD | TCSA-Cases | None | None | None | None |
| K19540 | frlA | fructoselysine transporter | TCSA-Cases | ko02000 Transporters | None | None | None |
| K19778 | hdeB | acid stress chaperone HdeB | TCSA-Cases | ko03110 Chaperones | None | None | None |

**Supplementary Table 6a**: The column KO indicates the KO item number based on the KEGG database, followed by the Gene Symbol and the complete Gene Name. Wilcoxon_group shows the sample that has been statistically associated with that KO. KO Brite group is the functional grouping of the KO. The LEfSE, DESeq2, and EdgeR indicate the sample statistically associated with that KO with the different algorithms. All the results were considered statistically valid with FDR<0.05, "none" no statistical association was found.
