# Supplementary material for: A unicentric cross-sectional observational study on chronic intestinal inflammation in total colonic aganglionosis: beware of an underestimated condition
Source: Orphanet J Rare Dis. 2023 Oct 27;18:339. doi: 10.1186/s13023-023-02958-1 (PMC10612252; doi:10.1186/s13023-023-02958-1)
Supplement: Supplementary file 4 — Supplementary Material 4 [file 13023_2023_2958_MOESM4_ESM.docx]

**Supplementary Table 7b**

| **KO** | **Gene Symbol** | **Gene Name** | **Wilcoxon_group** | **KO Brite group** | **LEfSe_group** | **DESeq2_group** | **EdgeR_group** |
| --- | --- | --- | --- | --- | --- | --- | --- |
| K13730 | inlA | internalin A | TCSA-Controls | None | None | None | None |
| K01777 | prdF | proline racemase [EC:5.1.1.4] | TCSA-Controls | ko01000 Enzymes | TCSA-Controls | TCSA-Controls | TCSA-Controls |
| K10793 | prdA | D-proline reductase (dithiol) PrdA [EC:1.21.4.1] | TCSA-Controls | ko01000 Enzymes | TCSA-Controls | TCSA-Controls | TCSA-Controls |
| K06896 | mapP | maltose 6'-phosphate phosphatase [EC:3.1.3.90] | TCSA-Controls | ko01000 Enzymes | None | TCSA-Controls | TCSA-Controls |
| K18887 | efrA, efrE | ATP-binding cassette, subfamily B, multidrug efflux pump | TCSA-Controls | ko02000 Transporters | None | TCSA-Controls | TCSA-Controls |
| K19431 | epsO | pyruvyl transferase EpsO [EC:2.-.-.-] | TCSA-Controls | ko01000 Enzymes | TCSA-Controls | TCSA-Controls | TCSA-Controls |
| K00131 | gapN | glyceraldehyde-3-phosphate dehydrogenase (NADP+) [EC:1.2.1.9] | TCSA-Controls | ko01000 Enzymes | None | None | None |
| K18888 | efrB, efrF | ATP-binding cassette, subfamily B, multidrug efflux pump | TCSA-Controls | ko02000 Transporters | None | TCSA-Controls | TCSA-Controls |
| K07768 | senX3 | two-component system, OmpR family, sensor histidine kinase SenX3 [EC:2.7.13.3] | TCSA-Controls | ko01000 Enzymes | None | TCSA-Controls | TCSA-Controls |
| K10795 | prdD | D-proline reductase (dithiol)-stabilizing protein PrdD | TCSA-Controls | None | None | None | None |
| K10796 | prdE | D-proline reductase (dithiol)-stabilizing protein PrdE | TCSA-Controls | None | None | None | None |

**Supplementary Table 6b**: The column KO indicates the KO item number based on the KEGG database, followed by the Gene Symbol and the complete Gene Name. Wilcoxon_group shows the sample that has been statistically associated with that KO. KO Brite group is the functional grouping of the KO. The LEfSE, DESeq2, and EdgeR indicate the sample statistically associated with that KO with the different algorithms. All the results were considered statistically valid with FDR<0.05, "none" no statistical association was found.
