# Supplementary material for: A unicentric cross-sectional observational study on chronic intestinal inflammation in total colonic aganglionosis: beware of an underestimated condition
Source: Orphanet J Rare Dis. 2023 Oct 27;18:339. doi: 10.1186/s13023-023-02958-1 (PMC10612252; doi:10.1186/s13023-023-02958-1)
Supplement: Supplementary file 5 — Supplementary Material 5 [file 13023_2023_2958_MOESM5_ESM.docx]

**Supplementary Table 1** – Questionnaire submitted to all enrolled patients (n = 38) to address clinical behaviour and identify possible risk factors or biomarkers.

| **Question** | **Possible answers** |
| --- | --- |
| Abdominal Pain | Never |
|  | Not invalidating abdominal pain |
|  | Invalidating abdominal pain |
| Abdominal distension | Yes |
|  | No |
| Rectal Bleeding | Never |
|  | Less than 50% of bowel movements |
|  | More than 50% of bowel movements |
| Stools consistence | Partially formed |
|  | Liquid |
| Number of bowel movements daily | 3 to 5 |
|  | More than 5 |
| Night-time bowel movements | Yes |
|  | No |
| Limitation of daily activities | No limitation |
|  | Occasionally |
|  | Often |
| Dietary limitation | Yes (specify) |
|  | No |
| Iron supplementation | Yes |
|  | No |
| Blood transfusion (in any occasion but surgery) | Yes |
|  | No |
| Antibiotic therapy (last month) | Yes (specify) |
|  | No |
| Anti-inflammatory medications (last month) | Yes (specify) |
|  | No |
