# Supplementary material for: A unicentric cross-sectional observational study on chronic intestinal inflammation in total colonic aganglionosis: beware of an underestimated condition
Source: Orphanet J Rare Dis. 2023 Oct 27;18:339. doi: 10.1186/s13023-023-02958-1 (PMC10612252; doi:10.1186/s13023-023-02958-1)
Supplement: Supplementary file 6 — Supplementary Material 6 [file 13023_2023_2958_MOESM6_ESM.docx]

**Supplemental Table 2.** Microbiome relative abundance analysis on the TCSA-Cases (Cases) compared to TCSA-Controls (Controls) samples.

| **TCSA-Cases [8] *vs* TCSA-Controls [19]** | **zero-inflated Gaussian fit** | **EdgeR** | | **DESeq2** | | **LDA** | |
| --- | --- | --- | --- | --- | --- | --- | --- |
| Taxonomy | FDR | log2FC | FDR | log2FC | FDR | LDA-SCORE | FDR |
| **Higher abundance in TCSA-Cases samples** | | | | | |  | |
| *p_Actinobacteria; c*_*Actinobacteria;* ***o_Actinomycetales*** |  | 2.7565 | 0.0082 |  |  |  |  |
| *p_Actinobacteria; c*_*Actinobacteria; o_Actinomycetales;* ***f_Micrococcaceae*** |  | 3.6332 | 0.0020 |  |  |  |  |
|  |  |  |  |  |  |  |  |
| *p_*[*Bacteroidetes*](https://www.ncbi.nlm.nih.gov/Taxonomy/Browser/wwwtax.cgi?mode=Undef&id=976&lvl=3&lin=f&keep=1&srchmode=1&unlock)*; c_*[*Bacteroidia*](https://www.ncbi.nlm.nih.gov/Taxonomy/Browser/wwwtax.cgi?mode=Undef&id=200643&lvl=3&lin=f&keep=1&srchmode=1&unlock)*; o_*[*Bacteroidales*](https://www.ncbi.nlm.nih.gov/Taxonomy/Browser/wwwtax.cgi?mode=Undef&id=171549&lvl=3&lin=f&keep=1&srchmode=1&unlock)*; f_Bacteroidaceae;* ***g_Bacteroides; s_dorei*** | 0.0024 |  |  |  |  |  |  |
|  |  |  |  |  |  |  |  |
| *p_*[*Firmicutes*](https://www.ncbi.nlm.nih.gov/Taxonomy/Browser/wwwtax.cgi?mode=Undef&id=1239&lvl=3&lin=f&keep=1&srchmode=1&unlock)*;****c_***[***Bacilli***](https://www.ncbi.nlm.nih.gov/Taxonomy/Browser/wwwtax.cgi?mode=Undef&id=91061&lvl=3&lin=f&keep=1&srchmode=1&unlock) |  | 4.5461 | 2.68E-4 |  |  |  |  |
| *p_*[*Firmicutes*](https://www.ncbi.nlm.nih.gov/Taxonomy/Browser/wwwtax.cgi?mode=Undef&id=1239&lvl=3&lin=f&keep=1&srchmode=1&unlock)*; c_*[*Bacilli*](https://www.ncbi.nlm.nih.gov/Taxonomy/Browser/wwwtax.cgi?mode=Undef&id=91061&lvl=3&lin=f&keep=1&srchmode=1&unlock)*;****o_***[***Bacillales***](https://www.ncbi.nlm.nih.gov/Taxonomy/Browser/wwwtax.cgi?mode=Undef&id=186826&lvl=3&lin=f&keep=1&srchmode=1&unlock) |  | 4.0074 | 3.37E-4 |  |  |  |  |
| *p_*[*Firmicutes*](https://www.ncbi.nlm.nih.gov/Taxonomy/Browser/wwwtax.cgi?mode=Undef&id=1239&lvl=3&lin=f&keep=1&srchmode=1&unlock)*; c_*[*Bacilli*](https://www.ncbi.nlm.nih.gov/Taxonomy/Browser/wwwtax.cgi?mode=Undef&id=91061&lvl=3&lin=f&keep=1&srchmode=1&unlock)*; o_*[*Bacillales*](https://www.ncbi.nlm.nih.gov/Taxonomy/Browser/wwwtax.cgi?mode=Undef&id=186826&lvl=3&lin=f&keep=1&srchmode=1&unlock)*;* ***f_Bacillaceae*** |  | 4.4687 | 1.18E-4 |  |  |  |  |
| *p_*[*Firmicutes*](https://www.ncbi.nlm.nih.gov/Taxonomy/Browser/wwwtax.cgi?mode=Undef&id=1239&lvl=3&lin=f&keep=1&srchmode=1&unlock)*; c_*[*Bacilli*](https://www.ncbi.nlm.nih.gov/Taxonomy/Browser/wwwtax.cgi?mode=Undef&id=91061&lvl=3&lin=f&keep=1&srchmode=1&unlock)*;****o_***[***Lactobacillales***](https://www.ncbi.nlm.nih.gov/Taxonomy/Browser/wwwtax.cgi?mode=Undef&id=186826&lvl=3&lin=f&keep=1&srchmode=1&unlock) |  | 4.3591 | 0.0010 |  |  |  |  |
| *p_*[*Firmicutes*](https://www.ncbi.nlm.nih.gov/Taxonomy/Browser/wwwtax.cgi?mode=Undef&id=1239&lvl=3&lin=f&keep=1&srchmode=1&unlock)*; c_*[*Bacilli*](https://www.ncbi.nlm.nih.gov/Taxonomy/Browser/wwwtax.cgi?mode=Undef&id=91061&lvl=3&lin=f&keep=1&srchmode=1&unlock)*; o_*[*Lactobacillales*](https://www.ncbi.nlm.nih.gov/Taxonomy/Browser/wwwtax.cgi?mode=Undef&id=186826&lvl=3&lin=f&keep=1&srchmode=1&unlock); ***f_Lactobacillaceae*** | 0.0274 |  |  | 6.6126 | 2.50E-4 |  |  |
| *p_*[*Firmicutes*](https://www.ncbi.nlm.nih.gov/Taxonomy/Browser/wwwtax.cgi?mode=Undef&id=1239&lvl=3&lin=f&keep=1&srchmode=1&unlock)*; c_*[*Bacilli*](https://www.ncbi.nlm.nih.gov/Taxonomy/Browser/wwwtax.cgi?mode=Undef&id=91061&lvl=3&lin=f&keep=1&srchmode=1&unlock)*; o_*[*Lactobacillales*](https://www.ncbi.nlm.nih.gov/Taxonomy/Browser/wwwtax.cgi?mode=Undef&id=186826&lvl=3&lin=f&keep=1&srchmode=1&unlock)*; f_Lactobacillaceae;* ***g_Lactobacillus*** |  | 7.0999 | 4.60E-4 |  |  |  |  |
| *p_*[*Firmicutes*](https://www.ncbi.nlm.nih.gov/Taxonomy/Browser/wwwtax.cgi?mode=Undef&id=1239&lvl=3&lin=f&keep=1&srchmode=1&unlock)*; c_Negativicutes; o_Selenomonadales; f_Veillonellaceae;* ***g_Veillonella; s_alcalescens*** | 0.0090 | 5.1759 | 0.0039 |  |  |  |  |
| *p_*[*Firmicutes*](https://www.ncbi.nlm.nih.gov/Taxonomy/Browser/wwwtax.cgi?mode=Undef&id=1239&lvl=3&lin=f&keep=1&srchmode=1&unlock)*; c_Negativicutes; o_Selenomonadales; f_Veillonellaceae;* ***g_Veillonella; s_parvula*** | 6.33E-4 | 6.0704 | 8.72E-4 |  |  |  |  |
|  |  |  |  |  |  |  |  |
| ***p_Fusobacteria*** |  | 4.7225 | 0.0273 | 4.9555 | 0.0301 |  |  |
| *p_Fusobacteria;* ***c_Fusobacteriia*** | 0.0145 |  |  |  |  |  |  |
| *p_Fusobacteria;* *c_Fusobacteriia;****o_Fusobacteriales*** | 0.0186 |  |  | 5.6368 | 0.0190 |  |  |
| *p_Fusobacteria;* *c_Fusobacteriia;o_Fusobacteriales;* ***f_Fusobacteriaceae*** | 0.0145 |  |  |  |  |  |  |
|  |  |  |  |  |  |  |  |
| ***p_***[***Proteobacteria***](https://www.ncbi.nlm.nih.gov/Taxonomy/Browser/wwwtax.cgi?mode=Undef&id=1224&lvl=3&lin=f&keep=1&srchmode=1&unlock) |  | 4.9753 | 1.01E-10 | 1.286 | 0.0127 |  |  |
| *p_*[*Proteobacteria*](https://www.ncbi.nlm.nih.gov/Taxonomy/Browser/wwwtax.cgi?mode=Undef&id=1224&lvl=3&lin=f&keep=1&srchmode=1&unlock)*;****c_Gammaproteobacteria*** |  | 4.3578 | 4.75E-5 | 2.0565 | 0.0177 |  |  |
| *p_*[*Proteobacteria*](https://www.ncbi.nlm.nih.gov/Taxonomy/Browser/wwwtax.cgi?mode=Undef&id=1224&lvl=3&lin=f&keep=1&srchmode=1&unlock)*; c_Gammaproteobacteria;* ***o_Enterobacteriales*** |  | 2.9045 | 0.0085 |  |  |  |  |
| *p_*[*Proteobacteria*](https://www.ncbi.nlm.nih.gov/Taxonomy/Browser/wwwtax.cgi?mode=Undef&id=1224&lvl=3&lin=f&keep=1&srchmode=1&unlock)*; c_Gammaproteobacteria;* *o_Enterobacteriales; f_Enterobacteriaceae;* ***g_Enterobacter*** |  | 4.2143 | 6.05E-4 |  |  |  |  |
| *p_*[*Proteobacteria*](https://www.ncbi.nlm.nih.gov/Taxonomy/Browser/wwwtax.cgi?mode=Undef&id=1224&lvl=3&lin=f&keep=1&srchmode=1&unlock)*; c_Gammaproteobacteria;* *o_Enterobacteriales; f_Enterobacteriaceae;* ***g_Escherichia*** |  | 3.0915 | 0.0321 |  |  |  |  |
| *p_*[*Proteobacteria*](https://www.ncbi.nlm.nih.gov/Taxonomy/Browser/wwwtax.cgi?mode=Undef&id=1224&lvl=3&lin=f&keep=1&srchmode=1&unlock)*; c_Gammaproteobacteria;* *o_Enterobacteriales; f_Enterobacteriaceae;* ***g_Escherichia; sp_coli*** |  | 3.0108 | 0.0330 |  |  |  |  |
| **Higher abundance in TCSA-Controls samples** | | | | | |  | |
| ***p_Actinobacteria*** | 7.05E-4 | -5.4148 | 5.05E-4 | -4.5844 | 1.24E-4 | 3.61 | 0.0404 |
| *p_Actinobacteria;* ***c*_*Actinobacteria*** | 0.0067 | -5.1588 | 0.0077 | -3.2638 | 0.0177 | 3.61 | 0.0370 |
| *p_Actinobacteria; c*_*Actinobacteria;* ***o_Bifidobacteriales*** | 0.0186 | -7.5256 | 0.0026 | -5.1232 | 0.0190 |  |  |
| *p_Actinobacteria; c*_*Actinobacteria; o_Bifidobacteriales;* ***f_Bifidobacteriaceae*** | 0.0041 | -7.1085 | 0.0054 |  |  |  |  |
| *p_Actinobacteria; c*_*Actinobacteria; o_Bifidobacteriales; f_Bifidobacteriaceae;* ***g_Bifidobacterium*** | 0.0114 | -8.1601 | 0.0038 | -5.3347 | 0.0372 |  |  |
| *p_Actinobacteria; c*_*Actinobacteria; o_Bifidobacteriales; f_Bifidobacteriaceae;* ***g_Bifidobacterium; s_adolescentis*** | 5.14E-5 | -10.002 | 0.0039 | -25.124 | 3.18E-16 |  |  |
| *p_Actinobacteria; c*_*Actinobacteria; o_Bifidobacteriales; f_Bifidobacteriaceae;* ***g_Bifidobacterium; s_longum*** | 0.0138 | -6.3081 | 0.0229 |  |  |  |  |
|  |  |  |  |  |  |  |  |
| *p_*[*Bacteroidetes*](https://www.ncbi.nlm.nih.gov/Taxonomy/Browser/wwwtax.cgi?mode=Undef&id=976&lvl=3&lin=f&keep=1&srchmode=1&unlock)*; c_*[*Bacteroidia*](https://www.ncbi.nlm.nih.gov/Taxonomy/Browser/wwwtax.cgi?mode=Undef&id=200643&lvl=3&lin=f&keep=1&srchmode=1&unlock)*; o_*[*Bacteroidales*](https://www.ncbi.nlm.nih.gov/Taxonomy/Browser/wwwtax.cgi?mode=Undef&id=171549&lvl=3&lin=f&keep=1&srchmode=1&unlock)*; f_Bacteroidaceae;* ***g_Bacteroides; s_fragilis*** | 0.0485 |  |  |  |  |  |  |
| *p_*[*Bacteroidetes*](https://www.ncbi.nlm.nih.gov/Taxonomy/Browser/wwwtax.cgi?mode=Undef&id=976&lvl=3&lin=f&keep=1&srchmode=1&unlock)*; c_*[*Bacteroidia*](https://www.ncbi.nlm.nih.gov/Taxonomy/Browser/wwwtax.cgi?mode=Undef&id=200643&lvl=3&lin=f&keep=1&srchmode=1&unlock)*; o_*[*Bacteroidales*](https://www.ncbi.nlm.nih.gov/Taxonomy/Browser/wwwtax.cgi?mode=Undef&id=171549&lvl=3&lin=f&keep=1&srchmode=1&unlock)*; f_Bacteroidaceae;* ***g_Bacteroides; s_sp.*** | 0.0020 |  |  |  |  |  |  |
|  |  |  |  |  |  |  |  |
| *p_*[*Firmicutes*](https://www.ncbi.nlm.nih.gov/Taxonomy/Browser/wwwtax.cgi?mode=Undef&id=1239&lvl=3&lin=f&keep=1&srchmode=1&unlock)*; c_Clostridia; o_Clostridiales; f_Clostridiaceae;* ***g_Clostridium; s_asparagiforme*** | 0.0024 |  |  |  |  |  |  |
| *p_*[*Firmicutes*](https://www.ncbi.nlm.nih.gov/Taxonomy/Browser/wwwtax.cgi?mode=Undef&id=1239&lvl=3&lin=f&keep=1&srchmode=1&unlock)*; c_Clostridia; o_Clostridiales; f_Clostridiaceae;* ***g_Clostridium; s_disporicum*** | 0.0054 | -5.2194 | 0.0258 |  |  |  |  |
| *p_*[*Firmicutes*](https://www.ncbi.nlm.nih.gov/Taxonomy/Browser/wwwtax.cgi?mode=Undef&id=1239&lvl=3&lin=f&keep=1&srchmode=1&unlock)*; c_Clostridia; o_Clostridiales; f_Clostridiaceae;* ***g_Clostridium; s_hiranonis*** | 6.33E-4 | -5.5629 | 0.0330 | -8.1082 | 0.0440 |  |  |
| *p_*[*Firmicutes*](https://www.ncbi.nlm.nih.gov/Taxonomy/Browser/wwwtax.cgi?mode=Undef&id=1239&lvl=3&lin=f&keep=1&srchmode=1&unlock)*; c_Clostridia; o_Clostridiales; f_Clostridiaceae;* ***g_Clostridium; s_mayombei*** | 6.33E-4 | -4.0056 | 0.0330 | -22.408 | 2.31E-15 |  |  |
| *p_*[*Firmicutes*](https://www.ncbi.nlm.nih.gov/Taxonomy/Browser/wwwtax.cgi?mode=Undef&id=1239&lvl=3&lin=f&keep=1&srchmode=1&unlock)*; c_Clostridia; o_Clostridiales; f_Lachnospiraceae;* ***g_Blautia; s_wexlerae*** | 0.0192 |  |  |  |  |  |  |
| *p_*[*Firmicutes*](https://www.ncbi.nlm.nih.gov/Taxonomy/Browser/wwwtax.cgi?mode=Undef&id=1239&lvl=3&lin=f&keep=1&srchmode=1&unlock)*; c_Clostridia; o_Clostridiales; f_Lachnospiraceae;* ***g_Dorea*** |  |  |  | -7.617 | 0.0338 |  |  |
| *p_*[*Firmicutes*](https://www.ncbi.nlm.nih.gov/Taxonomy/Browser/wwwtax.cgi?mode=Undef&id=1239&lvl=3&lin=f&keep=1&srchmode=1&unlock)*;****c_Erysipelotrichia*** | 0.0145 | -5.9307 | 0.0022 | -3.7465 | 0.0301 | 3.21 | 0.0370 |
| *p_*[*Firmicutes*](https://www.ncbi.nlm.nih.gov/Taxonomy/Browser/wwwtax.cgi?mode=Undef&id=1239&lvl=3&lin=f&keep=1&srchmode=1&unlock)*; c_Erysipelotrichia;* ***o_Erysipelotrichales*** | 0.0257 | -5.5425 | 0.0026 | -4.0221 | 0.0190 |  |  |
| *p_*[*Firmicutes*](https://www.ncbi.nlm.nih.gov/Taxonomy/Browser/wwwtax.cgi?mode=Undef&id=1239&lvl=3&lin=f&keep=1&srchmode=1&unlock)*; c_Erysipelotrichia;* ***o_****Erysipelotrichales;* ***f_Erysipelotrichaceae*** | 0.0439 | -5.2228 | 0.0088 |  |  |  |  |
| *p_*[*Firmicutes*](https://www.ncbi.nlm.nih.gov/Taxonomy/Browser/wwwtax.cgi?mode=Undef&id=1239&lvl=3&lin=f&keep=1&srchmode=1&unlock)*; c_Erysipelotrichia;* ***o_****Erysipelotrichales; f_Erysipelotrichaceae;* ***g_Clostridium; s_ramosum*** | 6.33E-4 | -4.0156 | 0.0330 |  |  |  |  |
| *p_*[*Firmicutes*](https://www.ncbi.nlm.nih.gov/Taxonomy/Browser/wwwtax.cgi?mode=Undef&id=1239&lvl=3&lin=f&keep=1&srchmode=1&unlock)*; c_*[*Clostridia*](https://www.ncbi.nlm.nih.gov/Taxonomy/Browser/wwwtax.cgi?mode=Undef&id=186801&lvl=3&lin=f&keep=1&srchmode=1&unlock)*; o_Clostridiales; f_*[*Ruminococcaceae*](https://www.ncbi.nlm.nih.gov/Taxonomy/Browser/wwwtax.cgi?mode=Undef&id=216572&lvl=3&lin=f&keep=1&srchmode=1&unlock)*;* ***g_Faecalibacterium*** |  |  |  | -7.5315 | 0.0338 |  |  |
| *p_*[*Firmicutes*](https://www.ncbi.nlm.nih.gov/Taxonomy/Browser/wwwtax.cgi?mode=Undef&id=1239&lvl=3&lin=f&keep=1&srchmode=1&unlock)*; c_*[*Clostridia*](https://www.ncbi.nlm.nih.gov/Taxonomy/Browser/wwwtax.cgi?mode=Undef&id=186801&lvl=3&lin=f&keep=1&srchmode=1&unlock)*; o_Clostridiales; f_*[*Ruminococcaceae*](https://www.ncbi.nlm.nih.gov/Taxonomy/Browser/wwwtax.cgi?mode=Undef&id=216572&lvl=3&lin=f&keep=1&srchmode=1&unlock)*;* ***g_Faecalibacterium; s_prausnitzii*** |  |  |  | -7.8724 | 0.0440 |  |  |
|  |  |  |  |  |  |  |  |
| *p_Fusobacteria;* *c_Fusobacteriia;o_Fusobacteriales; f_Fusobacteriaceae;* ***g_Fusobacterium; s_nucleatum*** | 0.0291 |  |  |  |  |  |  |
|  |  |  |  |  |  |  |  |
| *p_*[*Proteobacteria*](https://www.ncbi.nlm.nih.gov/Taxonomy/Browser/wwwtax.cgi?mode=Undef&id=1224&lvl=3&lin=f&keep=1&srchmode=1&unlock)*; c_Gammaproteobacteria;* ***o_Chromatiales*** | 0.0186 |  |  |  |  |  |  |
| *p_*[*Proteobacteria*](https://www.ncbi.nlm.nih.gov/Taxonomy/Browser/wwwtax.cgi?mode=Undef&id=1224&lvl=3&lin=f&keep=1&srchmode=1&unlock)*; c_Gammaproteobacteria;* *o_Chromatiales;* ***f_Chromatiaceae*** | 0.0145 |  |  |  |  |  |  |
| *p_*[*Proteobacteria*](https://www.ncbi.nlm.nih.gov/Taxonomy/Browser/wwwtax.cgi?mode=Undef&id=1224&lvl=3&lin=f&keep=1&srchmode=1&unlock)*; c_*[*Gammaproteobacteria*](https://www.ncbi.nlm.nih.gov/Taxonomy/Browser/wwwtax.cgi?mode=Undef&id=28216&lvl=3&lin=f&keep=1&srchmode=1&unlock)*;****o*_*Pasteurellales*** | 0.0214 | -4.2387 | 0.0312 | -4.6517 | 0.0190 |  |  |
| *p_*[*Proteobacteria*](https://www.ncbi.nlm.nih.gov/Taxonomy/Browser/wwwtax.cgi?mode=Undef&id=1224&lvl=3&lin=f&keep=1&srchmode=1&unlock)*; c_*[*Gammaproteobacteria*](https://www.ncbi.nlm.nih.gov/Taxonomy/Browser/wwwtax.cgi?mode=Undef&id=28216&lvl=3&lin=f&keep=1&srchmode=1&unlock)*; o*_*Pasteurellales;* ***f_Pasteurellaceae*** | 0.0145 |  |  |  |  |  |  |
| *p_*[*Proteobacteria*](https://www.ncbi.nlm.nih.gov/Taxonomy/Browser/wwwtax.cgi?mode=Undef&id=1224&lvl=3&lin=f&keep=1&srchmode=1&unlock)*; c_*[*Gammaproteobacteria*](https://www.ncbi.nlm.nih.gov/Taxonomy/Browser/wwwtax.cgi?mode=Undef&id=28216&lvl=3&lin=f&keep=1&srchmode=1&unlock)*; o*_*Pasteurellales; f_Pasteurellaceae;* ***g_Haemophylus*** | 0.0114 | -5.9461 | 0.0074 |  |  |  |  |
| *p_*[*Proteobacteria*](https://www.ncbi.nlm.nih.gov/Taxonomy/Browser/wwwtax.cgi?mode=Undef&id=1224&lvl=3&lin=f&keep=1&srchmode=1&unlock)*; c_*[*Gammaproteobacteria*](https://www.ncbi.nlm.nih.gov/Taxonomy/Browser/wwwtax.cgi?mode=Undef&id=28216&lvl=3&lin=f&keep=1&srchmode=1&unlock)*; o*_*Pasteurellales; f_Pasteurellaceae;* ***g_Haemophylus; s_parainfluenzae*** | 0.0032 |  |  |  |  |  |  |
| *p_*[*Proteobacteria*](https://www.ncbi.nlm.nih.gov/Taxonomy/Browser/wwwtax.cgi?mode=Undef&id=1224&lvl=3&lin=f&keep=1&srchmode=1&unlock)*; c_*[*Gammaproteobacteria*](https://www.ncbi.nlm.nih.gov/Taxonomy/Browser/wwwtax.cgi?mode=Undef&id=28216&lvl=3&lin=f&keep=1&srchmode=1&unlock)*; o*_*Pasteurellales; f_Pasteurellaceae;* ***g_Haemophylus; s_pittmaniae*** | 3.42E-4 | -6.9377 | 0.0045 | -22.161 | 3.30E-18 |  |  |
| *p_*[*Proteobacteria*](https://www.ncbi.nlm.nih.gov/Taxonomy/Browser/wwwtax.cgi?mode=Undef&id=1224&lvl=3&lin=f&keep=1&srchmode=1&unlock)*; c_*[*Gammaproteobacteria*](https://www.ncbi.nlm.nih.gov/Taxonomy/Browser/wwwtax.cgi?mode=Undef&id=28216&lvl=3&lin=f&keep=1&srchmode=1&unlock)*; o*_*Pasteurellales; f_Pasteurellaceae;* ***g_Haemophylus; s_sputorum*** | 0.0138 | -7.7475 | 0.0039 | -24.066 | 2.13E-23 |  |  |
| *p_*[*Proteobacteria*](https://www.ncbi.nlm.nih.gov/Taxonomy/Browser/wwwtax.cgi?mode=Undef&id=1224&lvl=3&lin=f&keep=1&srchmode=1&unlock)*; c_*[*Gammaproteobacteria*](https://www.ncbi.nlm.nih.gov/Taxonomy/Browser/wwwtax.cgi?mode=Undef&id=28216&lvl=3&lin=f&keep=1&srchmode=1&unlock)*; o*_*Pasteurellales; f_Pasteurellaceae;* ***g_Mannheimia*** | 0.0327 | -5.16 | 0.0074 |  |  |  |  |
| *p_*[*Proteobacteria*](https://www.ncbi.nlm.nih.gov/Taxonomy/Browser/wwwtax.cgi?mode=Undef&id=1224&lvl=3&lin=f&keep=1&srchmode=1&unlock)*; c_*[*Gammaproteobacteria*](https://www.ncbi.nlm.nih.gov/Taxonomy/Browser/wwwtax.cgi?mode=Undef&id=28216&lvl=3&lin=f&keep=1&srchmode=1&unlock)*; o*_*Pasteurellales; f_Pasteurellaceae;* ***g_Mannheimia; s_varigena*** | 0.0155 |  |  |  |  |  |  |

The number in square brackets indicates the number of patients in the groups compared in the analysis. The differential abundance analysis for microbial marker-gene used methods like “metagenomeSeq” and RNASeq. The “metagenomeSeq” use the zero-inflated Gaussian Fit algorithm. RNASeq is a differential abundance analysis method following EdgeR or DESeq2 algorithms. All statistical analyses adjust the data for imbalanced class distribution (under-sampling) and dataset sparsity. The taxa have been organized as *p_Phylum; c_Class; o_Order; f_Family; g_Genus; s_Specie*. FDR (False Discovery Rate) indicates the statistical significance p-value after adjustment for multiple comparisons. The base two logarithmic value of fold changes (log2FC) represents how much an increase (+) or decrease (-) in the abundance of a particular taxon in the comparisons between the indicated group of samples. A positive number indicates a higher wealth in the TCSA-Cases group of specimens, while negative values indicate the preferential presence in TCSA-Controls samples. FDR equal to or less than 0.05 was considered statistically significant.
