# Supplementary material for: A unicentric cross-sectional observational study on chronic intestinal inflammation in total colonic aganglionosis: beware of an underestimated condition
Source: Orphanet J Rare Dis. 2023 Oct 27;18:339. doi: 10.1186/s13023-023-02958-1 (PMC10612252; doi:10.1186/s13023-023-02958-1)
Supplement: Supplementary file 7 — Supplementary Material 7 [file 13023_2023_2958_MOESM7_ESM.docx]

**Supplemental Table 3. Supervised analysis with Random Forest learning algorithm**

**a**

| **TCSA-cases *versus* TCSA-controls** | | | | | |
| --- | --- | --- | --- | --- | --- |
| ***Genus*** | **Phylum** | **Mean Decrease Accuracy** | **Higher in** | **Sensitivity** | **Specificity** |
| *Escherichia* | **P** | 0,0188 | TCSA-cases | 0.78 | 0.75 |
| *Bifidobacterium* | **A** | 0,0089 | TCSA-controls | 0.78 | 0.75 |
| *Haemophilus* | **P** | 0,0076 | TCSA-controls | 0.78 | 0.75 |
| *Clostridium* | **F** | 0,0061 | TCSA-controls | 0.78 | 0.75 |
| *Lactobacillus* | **F** | 0,0053 | TCSA-cases | 0.78 | 0.75 |
| *Trabulsiella* | **P** | 0,0050 | TCSA-cases | 0.78 | 0.75 |
| *Streptococcus* | **F** | 0,0043 | TCSA-controls | 0.78 | 0.75 |
| ***Species*** |  |  |  |  |  |
| *Escherichia coli* | **P** | 0,0352 | TCSA-cases | 0.78 | 0.75 |
| *Trabulsiella odontotermitis* | **P** | 0,0088 | TCSA-cases | 0.78 | 0.75 |

**b**

| **CG-cases versus CG-controls** | | | | | |
| --- | --- | --- | --- | --- | --- |
| ***Genus*** | **Phylum** | **Mean Decrease Accuracy** | **Categorization group** | **Sensitivity** | **Specificity** |
| *Bifidobacterium* | **A** | 0.0120 | **CG-Cases** | 0.82 | 0.86 |
| *Eubacterium* | **F** | 0.0058 | **CG-Controls** | 0.82 | 0.86 |
| *Bilophila* | **P** | 0.0050 | **CG-Cases** | 0.82 | 0.86 |
| *Dialister* | **F** | 0.0040 | **CG-Cases** | 0.82 | 0.86 |
| *Sutterella* | **P** | 0.0039 | **CG-Controls** | 0.82 | 0.86 |
| ***Species*** |  |  |  |  |  |
| *Bacteroides sp.* | **B** | 0.0247 | **CG-Controls** | 0.85 | 0.78 |
| *Bacteroides merdae* | **B** | 0.0094 | **CG-Controls** | 0.85 | 0.78 |
| *Alistipes onderdonkii* | **B** | 0.0078 | **CG-Cases** | 0.85 | 0.78 |

Supervised Random Forest analysis of TCSA-Cases *versus* TCSA-Controls (**a**) or CG-Cases *versus* CG-Controls (**b**). The phylum column indicated to which the taxa belong: (A: *Actinobacteria*, B: *Bacteroidetes*, F: *Firmicutes*, P: *Proteobacteria*). Higher the value of Mean Decrease Accuracy, the more prominent the role of the relative taxa in the categorization group at which it was associated. The sensitivity and specificity (0-1) of the test's higher values are better.
