# Supplementary material for: A unicentric cross-sectional observational study on chronic intestinal inflammation in total colonic aganglionosis: beware of an underestimated condition
Source: Orphanet J Rare Dis. 2023 Oct 27;18:339. doi: 10.1186/s13023-023-02958-1 (PMC10612252; doi:10.1186/s13023-023-02958-1)
Supplement: Supplementary file 8 — Supplementary Material 8 [file 13023_2023_2958_MOESM8_ESM.docx]

**Supplemental Table 4.** Microbiome relative abundance analysis on the Care Givers-Cases (CG-Cases) compared to Care Givers-Controls (CG-Controls) samples.

| **CG-Cases [7] *vs* CG-Controls [15]** | **zero-inflated Gaussian fit** | **EdgeR** | | **DESeq2** | | **LDA** | |
| --- | --- | --- | --- | --- | --- | --- | --- |
| Taxonomy | FDR | log2FC | FDR | log2FC | FDR | LDA-SCORE | FDR |
| **Higher abundance in CG-Case samples** | | | | | |  | |
| *p_*[*Bacteroidetes*](https://www.ncbi.nlm.nih.gov/Taxonomy/Browser/wwwtax.cgi?mode=Undef&id=976&lvl=3&lin=f&keep=1&srchmode=1&unlock)*; c_*[*Bacteroidia*](https://www.ncbi.nlm.nih.gov/Taxonomy/Browser/wwwtax.cgi?mode=Undef&id=200643&lvl=3&lin=f&keep=1&srchmode=1&unlock)*; o_*[*Bacteroidales*](https://www.ncbi.nlm.nih.gov/Taxonomy/Browser/wwwtax.cgi?mode=Undef&id=171549&lvl=3&lin=f&keep=1&srchmode=1&unlock)*; f_Bacteroidaceae;* ***g_Bacteroides; s_fragilis*** | 0.0322 |  |  |  |  |  |  |
| *p_*[*Bacteroidetes*](https://www.ncbi.nlm.nih.gov/Taxonomy/Browser/wwwtax.cgi?mode=Undef&id=976&lvl=3&lin=f&keep=1&srchmode=1&unlock)*; c_*[*Bacteroidia*](https://www.ncbi.nlm.nih.gov/Taxonomy/Browser/wwwtax.cgi?mode=Undef&id=200643&lvl=3&lin=f&keep=1&srchmode=1&unlock)*; o_*[*Bacteroidales*](https://www.ncbi.nlm.nih.gov/Taxonomy/Browser/wwwtax.cgi?mode=Undef&id=171549&lvl=3&lin=f&keep=1&srchmode=1&unlock)*; f_Porphyromonadaceae;* ***g_Butyricimonas; s_virosa*** | 0.0076 |  |  |  |  |  |  |
| *p_*[*Bacteroidetes*](https://www.ncbi.nlm.nih.gov/Taxonomy/Browser/wwwtax.cgi?mode=Undef&id=976&lvl=3&lin=f&keep=1&srchmode=1&unlock)*; c_*[*Bacteroidia*](https://www.ncbi.nlm.nih.gov/Taxonomy/Browser/wwwtax.cgi?mode=Undef&id=200643&lvl=3&lin=f&keep=1&srchmode=1&unlock)*; o_*[*Bacteroidales*](https://www.ncbi.nlm.nih.gov/Taxonomy/Browser/wwwtax.cgi?mode=Undef&id=171549&lvl=3&lin=f&keep=1&srchmode=1&unlock)*; f_Rikenellaceae;* ***g_Alistipes; s_onderdonkii*** | 0.0449 |  |  |  |  |  |  |
|  |  |  |  |  |  |  |  |
| *p_*[*Firmicutes*](https://www.ncbi.nlm.nih.gov/Taxonomy/Browser/wwwtax.cgi?mode=Undef&id=1239&lvl=3&lin=f&keep=1&srchmode=1&unlock)*; c_Negativicutes; o_Selenomonadales; f_Acidaminococcaceae;* ***g_Phascolarctobacterium; s_*** ***faecium*** | 0.0449 |  |  |  |  |  |  |
|  |  |  |  |  |  |  |  |
| *p_*[*Proteobacteria*](https://www.ncbi.nlm.nih.gov/Taxonomy/Browser/wwwtax.cgi?mode=Undef&id=1224&lvl=3&lin=f&keep=1&srchmode=1&unlock)*;****c_Alphaproteobacteria*** |  | 2.1276 | 0.0226 |  |  |  |  |
| *p_*[*Proteobacteria*](https://www.ncbi.nlm.nih.gov/Taxonomy/Browser/wwwtax.cgi?mode=Undef&id=1224&lvl=3&lin=f&keep=1&srchmode=1&unlock)*; c_Alphaproteobacteria;* ***o_Magnetococcales*** |  | 3.9943 | 0.0242 |  |  |  |  |
| *p_*[*Proteobacteria*](https://www.ncbi.nlm.nih.gov/Taxonomy/Browser/wwwtax.cgi?mode=Undef&id=1224&lvl=3&lin=f&keep=1&srchmode=1&unlock)*; c_Alphaproteobacteria;* ***o_Rhizobiales*** |  | 3.997 | 0.0242 |  |  |  |  |
| **Higher abundance in CG-Control samples** | | | | | |  | |
| *p_*[*Bacteroidetes*](https://www.ncbi.nlm.nih.gov/Taxonomy/Browser/wwwtax.cgi?mode=Undef&id=976&lvl=3&lin=f&keep=1&srchmode=1&unlock)*; c_*[*Bacteroidia*](https://www.ncbi.nlm.nih.gov/Taxonomy/Browser/wwwtax.cgi?mode=Undef&id=200643&lvl=3&lin=f&keep=1&srchmode=1&unlock)*; o_*[*Bacteroidales*](https://www.ncbi.nlm.nih.gov/Taxonomy/Browser/wwwtax.cgi?mode=Undef&id=171549&lvl=3&lin=f&keep=1&srchmode=1&unlock)*; f_Bacteroidaceae;* ***g_Bacteroides; s_plebeius*** | 0.0025 | -9.3582 | 0.0049 | -8.1272 | 0.0041 |  |  |
| *p_*[*Bacteroidetes*](https://www.ncbi.nlm.nih.gov/Taxonomy/Browser/wwwtax.cgi?mode=Undef&id=976&lvl=3&lin=f&keep=1&srchmode=1&unlock)*; c_*[*Bacteroidia*](https://www.ncbi.nlm.nih.gov/Taxonomy/Browser/wwwtax.cgi?mode=Undef&id=200643&lvl=3&lin=f&keep=1&srchmode=1&unlock)*; o_*[*Bacteroidales*](https://www.ncbi.nlm.nih.gov/Taxonomy/Browser/wwwtax.cgi?mode=Undef&id=171549&lvl=3&lin=f&keep=1&srchmode=1&unlock)*; f_Bacteroidaceae;* ***g_Bacteroides; s_coprocola*** | 5.46E-7 | -11.617 | 0.0012 | -30.0 | 9.00E-32 |  |  |
| *p_*[*Bacteroidetes*](https://www.ncbi.nlm.nih.gov/Taxonomy/Browser/wwwtax.cgi?mode=Undef&id=976&lvl=3&lin=f&keep=1&srchmode=1&unlock)*; c_*[*Bacteroidia*](https://www.ncbi.nlm.nih.gov/Taxonomy/Browser/wwwtax.cgi?mode=Undef&id=200643&lvl=3&lin=f&keep=1&srchmode=1&unlock)*; o_*[*Bacteroidales*](https://www.ncbi.nlm.nih.gov/Taxonomy/Browser/wwwtax.cgi?mode=Undef&id=171549&lvl=3&lin=f&keep=1&srchmode=1&unlock)*; f_Bacteroidaceae;* ***g_Bacteroides; s_eggerthii*** |  | -6.3342 | 0.0243 | -25.123 | 6.93E-28 |  |  |
| *p_*[*Bacteroidetes*](https://www.ncbi.nlm.nih.gov/Taxonomy/Browser/wwwtax.cgi?mode=Undef&id=976&lvl=3&lin=f&keep=1&srchmode=1&unlock)*; c_*[*Bacteroidia*](https://www.ncbi.nlm.nih.gov/Taxonomy/Browser/wwwtax.cgi?mode=Undef&id=200643&lvl=3&lin=f&keep=1&srchmode=1&unlock)*; o_*[*Bacteroidales*](https://www.ncbi.nlm.nih.gov/Taxonomy/Browser/wwwtax.cgi?mode=Undef&id=171549&lvl=3&lin=f&keep=1&srchmode=1&unlock)*; f_Bacteroidaceae;* ***g_Bacteroides; s_sp.*** |  |  |  | -4.8168 | 0.0202 |  |  |
| *p_*[*Firmicutes*](https://www.ncbi.nlm.nih.gov/Taxonomy/Browser/wwwtax.cgi?mode=Undef&id=1239&lvl=3&lin=f&keep=1&srchmode=1&unlock)*; c_Clostridia; o_Clostridiales; f_Lachnospiraceae;* ***g_Roseburia; s_intestinalis*** | 0.0449 |  |  |  |  |  |  |
| *p_*[*Firmicutes*](https://www.ncbi.nlm.nih.gov/Taxonomy/Browser/wwwtax.cgi?mode=Undef&id=1239&lvl=3&lin=f&keep=1&srchmode=1&unlock)*; c_*[*Clostridia*](https://www.ncbi.nlm.nih.gov/Taxonomy/Browser/wwwtax.cgi?mode=Undef&id=186801&lvl=3&lin=f&keep=1&srchmode=1&unlock)*; o_Clostridiales; f_*[*Ruminococcaceae*](https://www.ncbi.nlm.nih.gov/Taxonomy/Browser/wwwtax.cgi?mode=Undef&id=216572&lvl=3&lin=f&keep=1&srchmode=1&unlock)*;* ***g_Ruminococcus; s_lactaris*** | 0.0076 |  |  |  |  |  |  |

The number in square brackets indicates the number of patients in the groups compared in the analysis. The differential abundance analysis for microbial marker-gene used methods like “metagenomeSeq” and RNASeq. The “metagenomeSeq” use the zero-inflated Gaussian Fit algorithm. RNASeq is a differential abundance analysis method following EdgeR or DESeq2 algorithms. All statistical analyses adjust the data for imbalanced class distribution (under-sampling) and dataset sparsity. The taxa have been organized as *p_Phylum; c_Class; o_Order; f_Family; g_Genus; s_Specie*. FDR (False Discovery Rate) indicates the statistical significance p-value after adjustment for multiple comparisons. The base two logarithmic value of fold changes (log2FC) represents how much an increase (+) or decrease (-) in the abundance of a particular taxon in the comparisons between the indicated group of samples. A positive number indicates a higher wealth in the TCSA-Cases group of specimens, while negative values indicate the preferential presence in TCSA-Controls samples. FDR equal to or less than 0.05 was considered statistically significant.
