# Supplementary figures and images for: A unicentric cross-sectional observational study on chronic intestinal inflammation in total colonic aganglionosis: beware of an underestimated condition
Source: Orphanet J Rare Dis. 2023 Oct 27;18:339. doi: 10.1186/s13023-023-02958-1 (PMC10612252; doi:10.1186/s13023-023-02958-1)

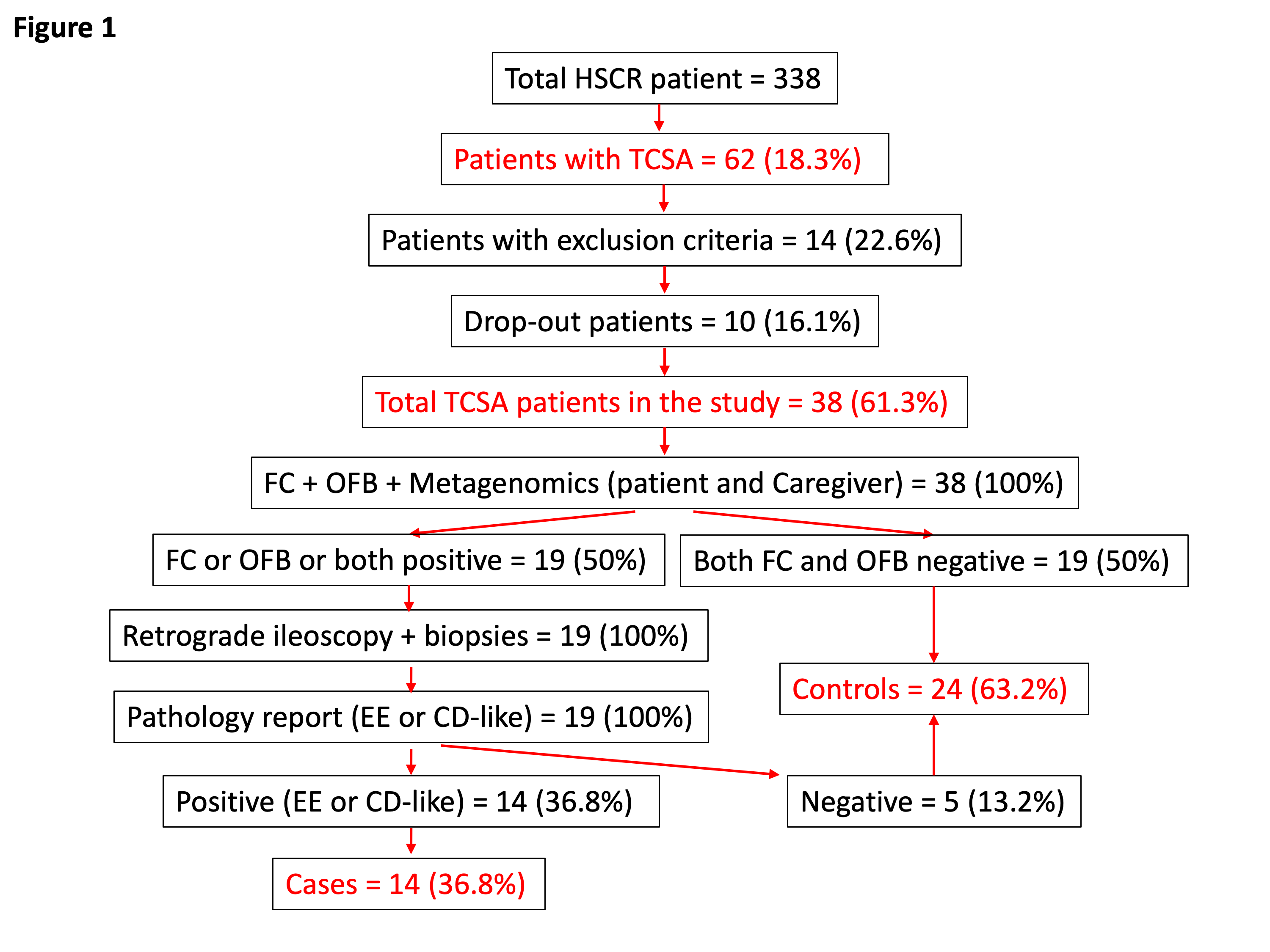

Supplement: Supplementary file 9 — Supplementary Material 9 [file 13023_2023_2958_MOESM9_ESM.tiff]
